# Drawing Links from Transcriptome to Metabolites: The Evolution of Aroma in the Ripening Berry of Moscato Bianco (*Vitis vinifera* L.)

**DOI:** 10.3389/fpls.2017.00780

**Published:** 2017-05-16

**Authors:** Laura Costantini, Christian D. Kappel, Massimiliano Trenti, Juri Battilana, Francesco Emanuelli, Maddalena Sordo, Marco Moretto, Céline Camps, Roberto Larcher, Serge Delrot, Maria S. Grando

**Affiliations:** ^1^Grapevine Genetics and Breeding Unit, Genomics and Biology of Fruit Crop Department, Research and Innovation Centre, Fondazione Edmund MachSan Michele all'Adige, Italy; ^2^UMR Ecophysiology and Grape Functional Genomics, Institut des Sciences de la Vigne et du Vin, University of BordeauxVillenave d'Ornon, France; ^3^Computational Biology Platform, Research and Innovation Centre, Fondazione Edmund MachSan Michele all'Adige, Italy; ^4^Experiment and Technological Services Department, Technology Transfer Centre, Fondazione Edmund MachSan Michele all'Adige, Italy; ^5^Center Agriculture Food Environment, University of TrentoSan Michele all'Adige, Italy

**Keywords:** grapevine, Muscat, monoterpene, development, berry skin, metabolic and transcript profiling, integration, candidate gene

## Abstract

Monoterpenes confer typical floral notes to “Muscat” grapevine varieties and, to a lesser extent, to other aromatic non-Muscat varieties. Previous studies have led to the identification and functional characterization of some enzymes and genes in this pathway. However, the underlying genetic map is still far from being complete. For example, the specific steps of monoterpene metabolism and its regulation are largely unknown. With the aim of identifying new candidates for the missing links, we applied an integrative functional genomics approach based on the targeted metabolic and genome-wide transcript profiling of Moscato Bianco ripening berries. In particular, gas chromatography-mass spectrometry analysis of free and bound terpenoid compounds was combined with microarray analysis in the skins of berries collected at five developmental stages from pre-*veraison* to over-ripening. Differentially expressed metabolites and probes were identified in the pairwise comparison between time points by using the early stage as a reference. Metabolic and transcriptomic data were integrated through pairwise correlation and clustering approaches to discover genes linked with particular metabolites or groups of metabolites. These candidate transcripts were further checked for co-localization with quantitative trait loci (QTLs) affecting aromatic compounds. Our findings provide insights into the biological networks of grapevine secondary metabolism, both at the catalytic and regulatory levels. Examples include a nudix hydrolase as component of a terpene synthase-independent pathway for monoterpene biosynthesis, genes potentially involved in monoterpene metabolism (cytochrome P450 hydroxylases, epoxide hydrolases, glucosyltransferases), transport (vesicle-associated proteins, ABCG transporters, glutathione S-transferases, amino acid permeases), and transcriptional control (transcription factors of the ERF, MYB and NAC families, intermediates in light- and circadian cycle-mediated regulation with supporting evidence from the literature and additional regulatory genes with a previously unreported association to monoterpene accumulation).

## Introduction

A great deal of the consumer interest in wine derives from its aroma characteristics. The major aroma-impact compounds in grape and wine are terpenoids (monoterpenes, sesquiterpenes, and in a wider acception also C_13_-norisoprenoids), phenylpropanoids/benzenoids, fatty acid derivatives, sulfur compounds, and methoxypyrazines (Dunlevy et al., 2009; Ebeler and Thorngate, 2009; Panighel and Flamini, 2014; Robinson et al., 2014; Black et al., 2015). The typical floral and citrus attributes of Muscat varieties are primarily determined by a combination of linalool, geraniol and nerol (Ribéreau-Gayon et al., 2000). The same monoterpenes contribute to the varietal aroma of Riesling in association with the linalool oxides, hydroxy-linalool, α-terpineol, citronellol, terpendiol I and hydroxy-trienol (Rapp, 1998). Likewise rose oxide, which is highly correlated with Muscat score in grapes (Ruiz-García et al., 2014), is also a potent odorant in Scheurebe and Gewürztraminer (Guth, 1997; Ong and Acree, 1999; Luan et al., 2005).

The terpene biosynthetic pathway is generally well known (Dudareva et al., 2013), even though a number of alternative non-canonical reactions may occur (Sun et al., 2016). Of the two systems responsible for the production of plant isopentenyl diphosphate (IPP) and dimethylallyl diphosphate (DMAPP), the primarily cytosolic mevalonic acid (MVA) and the plastidial methylerythritol phosphate (MEP) pathway, the latter has been suggested as the dominant route for monoterpene biosynthesis in grape berries (Luan and Wüst, 2002). Several lines of evidence (Battilana et al., 2009, 2011; Duchêne et al., 2009; Emanuelli et al., 2010; Martin et al., 2012; Wen et al., 2015) support the existence of at least two rate-limiting enzymes in the grapevine MEP pathway, namely the first (1-deoxy-D-xylulose 5-phosphate synthase, VvDXS1) and the last (4-hydroxy-3-methylbut-2-enyl diphosphate reductase, VvHDR). Both IPP and DMAPP are substrates for short-chain prenyltransferases, which produce prenyl diphosphate precursors for the large family of terpene synthases (TPSs). To date around 40 full-length VvTPSs out of 53–89 predicted functional enzymes have been biochemically characterized (Martin et al., 2010) and some major players in grape Muscat aroma have been identified, like the α-terpineol synthase *VvTer*, the linalool synthase *Lis*, the linalool/nerolidol synthase *VvPNLinNer1* and the geraniol synthase *VvPNGer* (Ebang-Oke et al., 2003; Martin and Bohlmann, 2004; Martin et al., 2012; Matarese et al., 2013; Zhu et al., 2014; Wen et al., 2015). Once a terpenoid alcohol skeleton has been produced, extensive modifications determine the final monoterpene composition of grapes and wines (Ribéreau-Gayon et al., 1975; Williams et al., 1989; Luan et al., 2004, 2005, 2006a,b; Mathieu et al., 2009). These secondary transformations are (at least in part) catalyzed by enzymes (Luan et al., 2006a; D'Onofrio et al., 2016) that in most cases have not been identified. The only exceptions are the three grape monoterpenol β-D-glucosyltransferases VvGT7, VvGT14 and VvGT15 and the cytochrome P450 CYP76F14 (Bönisch et al., 2014a,b; Ilc et al., 2017). The main reason for this gap is that such enzymes belong to large families with broad substrate tolerance and overlapping activities (Schwab, 2003; Nelson et al., 2008; Schwab and Wüst, 2015). A better knowledge of the missing enzymes might allow us to manipulate the formation of grape aroma compounds. For example, limiting the reactions responsible for the depletion of key odorants (e.g., through the selection of genotypes with low monoterpene glycosyltransferase or oxygenase activities in breeding programs) could be an alternative approach for the improvement of grape/wine flavor (Bönisch et al., 2014a; Hjelmeland and Ebeler, 2015).

The grapevine terpenoid pathway is intricately regulated by endogenous and environmental factors that enable spatially and temporally controlled metabolite production (Ebeler and Thorngate, 2009; Robinson et al., 2014). In other plant species a network of transcription factors (TFs) is involved in the regulation of this pathway, including members of the AP2, AP2/ERF, bZIP, MYB, MYC, NAC, WRKY, and YABBY families (De Geyter et al., 2012; Patra et al., 2013; Nieuwenhuizen et al., 2015; Wang et al., 2016). A tight regulation of terpene biosynthesis is additionally exerted at the post-transcriptional level involving both structural enzymes and transcription factors (Vom Endt et al., 2002; Hemmerlin, 2013; Rodríguez-Concepción and Boronat, 2015), as observed also in *Vitis vinifera* (Bönisch et al., 2014a; Matarese et al., 2014). A number of transcription factors that might control terpene synthesis have been recently predicted in grapevine through gene co-expression network analysis (Wen et al., 2015), though none of them has been yet demonstrated to regulate the expression of relevant terpene pathway genes. Similarly, the reasons of the differential accumulation of the main monoterpenes in grape berry tissues across development (Günata et al., 1985; Wilson et al., 1986; Park et al., 1991; Luan and Wüst, 2002), which is reflected in the identification of specific QTLs for linalool and geraniol/nerol (Doligez et al., 2006; Battilana et al., 2009), are still unknown.

This work aims at a better understanding of aroma determination in grapevine and at the identification of candidate genes for further functional analysis. To this purpose, we integrated gas chromatography/mass spectrometry-based quantitative analysis of selected metabolites with microarray-based transcriptomic analysis in Moscato Bianco (*Vitis vinifera* L.) ripening berries. According to the observed associations between metabolite and transcript profiles, we report several genes that may control the accumulation of free and glycosidically bound monoterpenes and additional aroma-related compounds.

## Materials and methods

### Plant material for metabolic and microarray analyses

**Figure 1:**
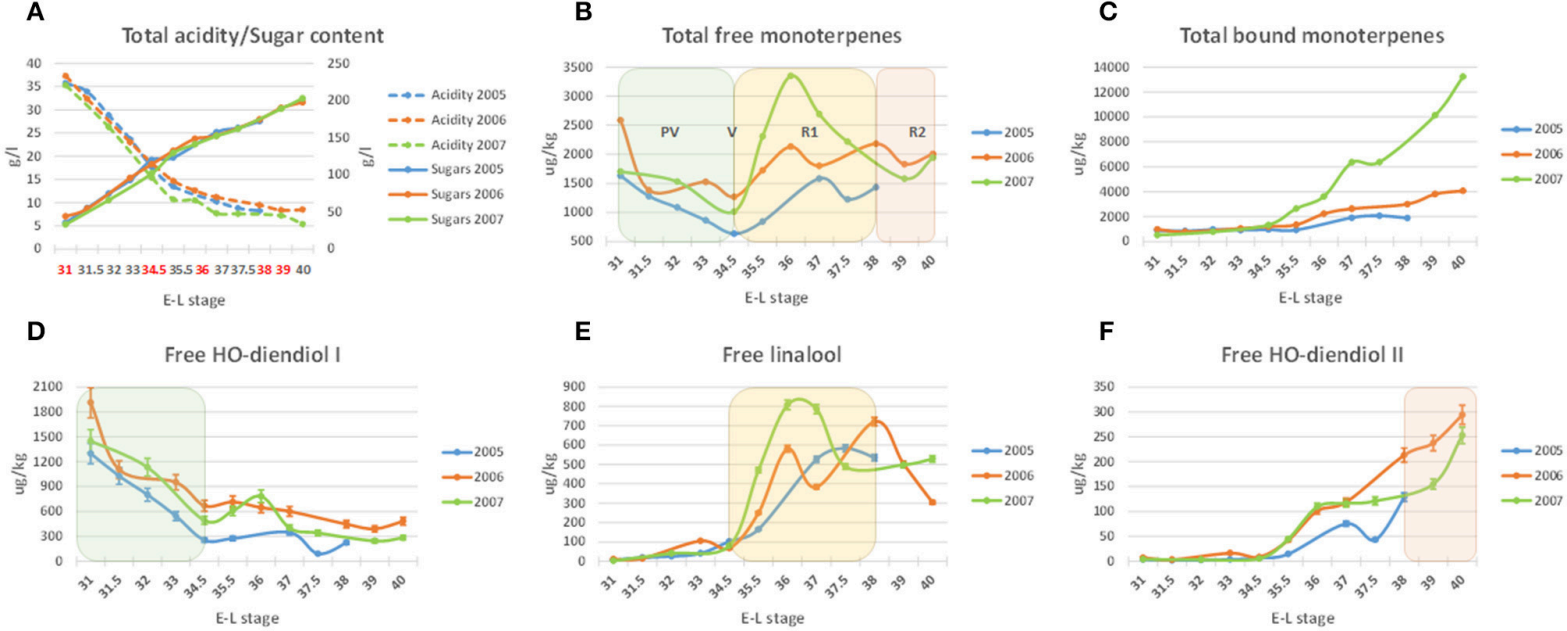
**Acidity, sugars (A)** and monoterpene content **(B–F)** of the Moscato Bianco samples collected during berry development in 2005, 2006, and 2007. The five stages assayed by microarray analysis in 2006 are highlighted with red in **(A)**. Exemplar monoterpenes with a major contribution to the total free monoterpene profile **(B)** during PV, R1, and R2 are shown in **(D–F)**, respectively. A single biological replicate was considered at each stage in each season; bars in **(D–F)** correspond to the standard error calculated from six technical replicates, as described in Supplementary Data1: Method S1. The metabolites were quantified by using solid SPE-HRGC-MS and referring to the internal standard 1-heptanol. The lines connecting data points were smoothed through the specific option provided by Excel. E-L stage, growth stage according to the modified Eichhorn-Lorenz scheme (Coombe, 1995); PV, pre-*veraison*; V, *veraison*; R1, ripening (till technological maturity or stage E-L 38); R2, over-ripening (after technological maturity). The decimal E-L stages were arbitrarily assigned by the authors of the present study to facilitate the alignment of the sampling dates from the three different seasons.

Berries of the cultivar Moscato Bianco (*Vitis vinifera* L.) were collected from pre-*veraison* to over-ripening in 2005, 2006, and 2007 (Figure 1A and Table 1). At each sampling date, ten bunches were taken from ten adult plants out of the ~ 250 grown on Kober 5BB rootstocks in the experimental fields of FEM (Fondazione Edmund Mach, San Michele all'Adige, Italy). Care was taken to sample from different vines and positions within each vine. In the lab, berries were pooled in order to minimize environmental effects and then divided into two (2005 and 2007) or three (2006) batches. Berries from the first batch were homogenized to juice (80 mL) and analyzed for titratable acidity and soluble solids content by FT-IR (Fourier Transform InfraRed) spectroscopy with a FOSS instrument (FOSS NIRSystems, Oatley, Australia). Berries from the second batch were stored at −80°C till metabolic analysis. Berries from the third batch were hand-peeled, the skins were immediately frozen in liquid nitrogen and stored at −80°C pending RNA extraction.

**Table 1:** **Acidity and sugar content of the Moscato Bianco samples collected during berry development in 2006**.

**E-L stage**	**Sample number in microarray experiment**	**Date**	**Weeks from *veraison***	**Malic acid (g l^−1^)**	**Tartaric acid (g l^−1^)**	**pH**	**Total acidity (g l^−1^)**	**Sugar content (from °Brix)**	**Sugar content (from relative density)**
**31**	**1**	**27/07/06**	−**3**	**26.8**	**11.3**	**2.4**	**37.3**	**44.1**	**52.4**
31.5		31/07/06		24.1	10.7	2.4	33.4	54.0	63.0
31.5		02/08/06	−2	21.9	10.9	2.4	31.3	54.7	63.3
33		08/08/06		16.1	8.9	2.6	23.5	96.4	105.5
33		10/08/06	−1	15.6	9.0	2.7	22.5	95.6	105.0
**34.5**	**2**	**17/08/06**	**0**	**12.3**	**7.9**	**2.7**	**18.5**	**114.3**	**122.6**
35.5		24/08/06	+1	8.7	8.1	2.9	14.7	132.4	140.0
**36**	**3**	**30/08/06**	+**2**	**6.7**	**8.0**	**3.0**	**12.6**	**148.5**	**156.5**
37		06/09/06	+3	5.9	7.9	3.1	11.2	152.8	160.9
**38**	**4**	**13/09/06**	+**4**	**5.3**	**8.1**	**3.2**	**10.4**	**172.7**	**182.6**
38		21/09/06	+5	3.7	7.4	3.3	8.6	176.7	185.9
**39**	**5**	**27/09/06**	+**6**	**3.8**	**7.9**	**3.3**	**8.4**	**190.0**	**200.1**
40		10/10/06	+8	3.3	8.2	3.4	8.5	197.7	206.6

*The five stages used for microarray analysis are in boldface. E-L stage, growth stage according to the modified Eichhorn-Lorenz scheme (Coombe, 1995). The decimal E-L stages were arbitrarily assigned by the authors of the present study to facilitate the alignment of sampling dates from three different seasons (Figure 1). The weeks from veraison were established with a maximum tolerance of 2 days from the exact date*.

### Metabolic analysis

Thirty-two aroma-active compounds were quantified in their free and glycosidically bound form by using solid phase extraction (SPE) and high-resolution gas chromatography-mass spectrometry (HRGC-MS; Supplementary Data1: Method S1 and Supplementary Table S1) in the growing seasons 2005, 2006, and 2007.

Network analysis for 2006 metabolic data included pairwise correlation, hierarchical clustering with bootstrapping (Pvclust with 10,000 resamplings, see Suzuki and Shimodaira, 2006) and principal component analysis (PCA) and was applied to different data sets (free and glycosidically bound metabolites, absolute quantities and differentials, 5 and 13 time points).

### Microarray analysis

**Figure 2:**
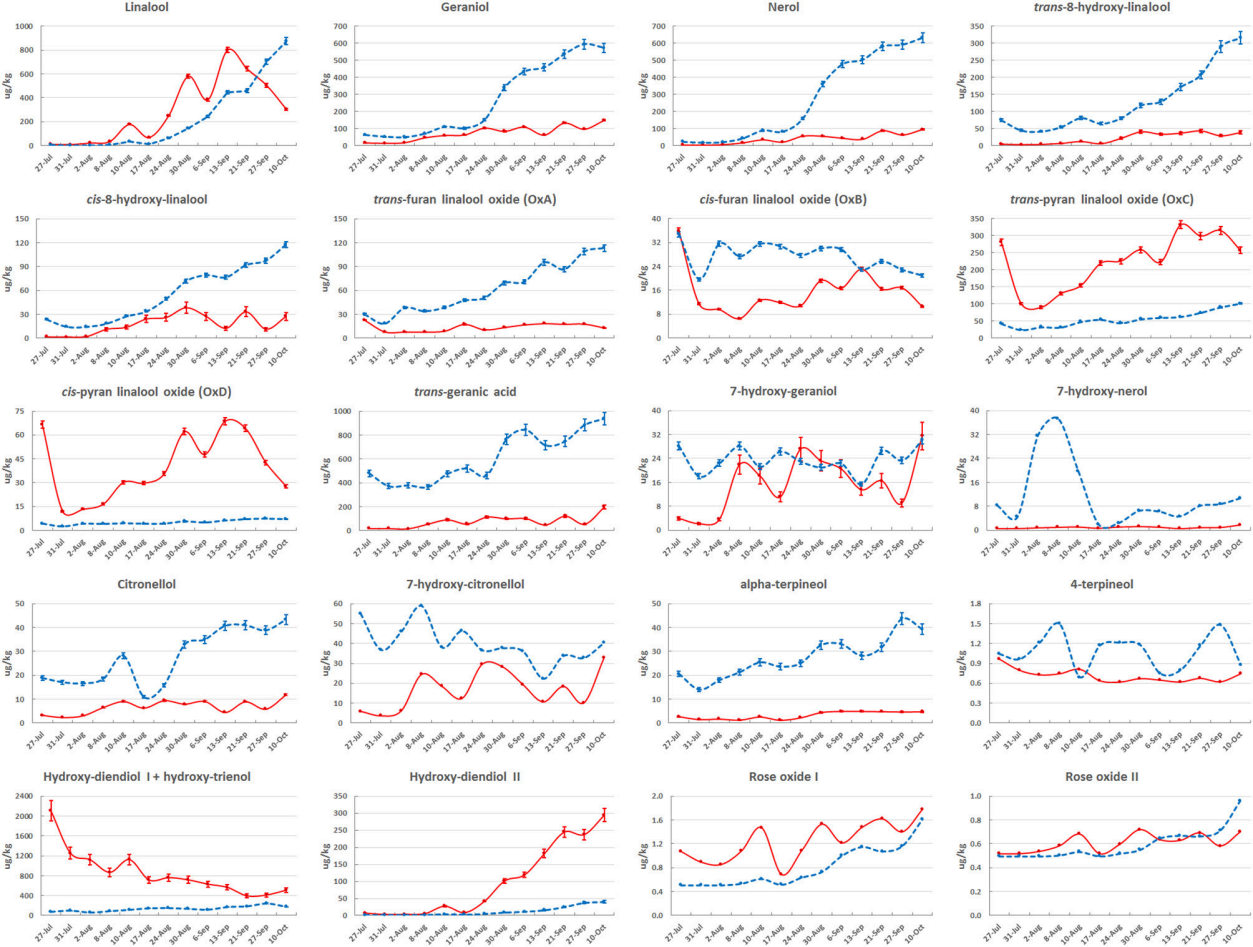
**Evolution of monoterpenoids in their free (solid red line) and glycosidically bound (dashed blue line) form during Moscato Bianco berry ripening in 2006**. A single biological replicate was considered at each stage; bars correspond to the standard error calculated from six technical replicates, as described in Supplementary Data1: Method S1 (technical replication is not available for 7-hydroxy-nerol, 7-hydroxy-citronellol, 4-terpineol, rose oxide I and II). The metabolites were quantified by using solid SPE-HRGC-MS and referring to the internal standard 1-heptanol. The lines connecting data points were smoothed through the specific option provided by Excel.

Based on monoterpene accumulation during berry development in 2006 (Figure 2), five time points were chosen along this season (Figure 1 and Table 1). Total RNA was extracted from grape skins using the Spectrum™ Plant Total RNA Kit (Sigma-Aldrich, St. Louis, Missouri, USA). RNA quantity and quality were evaluated with a NanoDrop ND-8000 spectrophotometer (NanoDrop Technologies, Wilmington, Delaware, USA) and an Agilent 2100 Bioanalyzer (Agilent Technologies, Mississauga, Ontario, Canada).

Microarray experiments were carried out with a 70-mer oligoarray containing all 14,562 probes from the Array-Ready Oligo Set™ (AROS) for the Grape (*Vitis vinifera*) Genome version 1.0 (Operon Biotechnologies, Huntsville, Alabama, USA). At the time this platform represented a good compromise between genome coverage, cost and computational effort required for data analysis. RNA from points 2 to 5 was hybridized competitively with RNA from point 1 (pre-*veraison*), following the dye-swap experiment design (Churchill, 2002). A total of sixteen slides were used (four comparisons: 2 vs. 1, 3 vs. 1, 4 vs. 1, 5 vs. 1; two biological and two technical replicates). The biological and technical replicates corresponded to two subgroups from the unique pool of berries (third batch) and to the dye swaps, respectively. Details for probe synthesis, hybridization and scanning are described in Supplementary Data1: Method S2.

Spot intensities were quantified with the software MAIA 2.75 (Novikov and Barillot, 2007). After excluding poor quality spots due to bad spotting (e.g., spots with irregular shapes or highly unequal intensity distributions), median intensity gene expression data without background subtraction were normalized by a global lowess method followed by a print-tip median method with a modified version of the Goulphar script version 1.1.2 (Lemoine et al., 2006). Differentially expressed probes (DEPs) with a false discovery rate (FDR) < 1% and a cut-off of 2-fold change (FC) were identified with the R/Bioconductor Limma package using linear models (Smyth, 2004) and taking into account biological and technical replicates by doing a two-factor analysis. The earliest sample was used as the reference to whom all the other samples were compared. A multiple testing correction (Benjamini and Hochberg, 1995) was applied to adjust the FDR. The full raw expression dataset is available at the Gene Expression Omnibus (GEO, http://www.ncbi.nlm.nih.gov/geo/) under the accession number GSE76834.

### Probe functional annotation

The 70-mer probes spotted on the Grape AROS V1.0 array represent 14,562 transcripts from The Institute for Genomic Research (TIGR) Grape Gene Index (VvGI), release 3 (August 13, 2003). The corresponding annotation is based upon a match between each oligo and the gene set of the 12X version of the grape genome at CRIBI (http://genomes.cribi.unipd.it/grape/) and is publicly available at GEO (http://www.ncbi.nlm.nih.gov/geo/) under the accession number GPL15453. Since this annotation provides every oligo with a text description but doesn't associate it to any gene prediction identifier, we independently achieved this information by blastN alignment against the grape gene sets at CRIBI (http://genomes.cribi.unipd.it/grape/, 12X version of the genome, V1 gene prediction, annotation from Grimplet et al., 2012) and IASMA (Velasco et al., 2007), as fully detailed in Supplementary Table S2. For the alignment against the CRIBI gene set, the following parameters were used: sequence identity ≥90%, minimum alignment length of 95%, maximum number of mismatches of 5 and maximum number of gaps of 5. The aligned 70-mers were found to correspond to 7,162 and 8,260 unique gene predictions at CRIBI and IASMA, respectively. The 14,562 probes were also grouped into main functional categories according to the Mapman BIN structure (Rotter et al., 2009; Supplementary Table S2).

### Microarray validation via real-time PCR

Real-time quantitative reverse transcription-PCR (qRT-PCR) was used to validate the microarray results. Since RNA from berries collected in 2006 was no more available, new samples (with three biological replicates from pooled berries) as closest as possible to those analyzed in 2006 were obtained in 2016 by adopting the same sampling procedure and the same protocol for RNA extraction.

Primers for the amplification of unique PCR products from 70 to 250 bp were designed on 15 *Vitis vinifera* gene predictions perfectly matching with the microarray 70-mers by using Primer3 (Untergasser et al., 2012), as reported in Supplementary Table S3. Details for the amplification reaction and expression analysis are described in Supplementary Data1: Method S3. The relationship between microarray and qRT-PCR data was established through Pearson correlation.

### Discovery of a link between transcriptome and metabolome in aroma development

#### Integration of 2006 transcriptomic and metabolic data

Different approaches were tested in order to discover transcripts linked with the accumulation of one or more metabolites. In a first identification step of candidates, the most stable expression changes were preferred to the biggest ones, hence the microarray probes with adjusted *p*-values < 0.05 in all comparisons were considered, irrespective of their fold change (4,450). Working at probe level instead of gene level was chosen for two main reasons: (1) different probes supposedly matching to the same gene (especially long genes) often show different expression values, which might be an indicator of alternative transcription and (2) the sequence specificity to CRIBI 12X gene predictions is not optimal for a number of probes spotted on the AROS array (this is especially true for probes related to secondary metabolism); for a detailed assessment of probe specificity, Moscato Bianco genome and transcriptome assembly would be required, which is out of the scope of this work.

##### Pairwise correlation

Pearson pairwise correlation was calculated between transcripts and metabolites across all the time points (log_2_-transformed differentials in the pairwise comparisons 2 vs. 1, 3 vs. 1, 4 vs. 1, and 5 vs. 1). With the goal of identifying aroma regulatory genes we also tested a two-step strategy, which was based on (1) search for candidate metabolism and transport genes by direct correlation with metabolites and (2) expression pairwise correlations between these enzyme/transporter-coding genes and any regulatory gene within the microarray. For this aim, Pearson correlations were computed both between differential gene expression ratios (*n* = 4) and microarray channel intensities (*n* = 32, when considering technical replicates separately).

##### Correlation biclustering

Based on the assumption that a gene might regulate the accumulation of a metabolite only at specific stages during ripening, correlation biclustering between transcripts and metabolites was achieved with QUBIC (Li et al., 2009) (log_2_-transformed data, Pearson correlation). Compared to the traditional clustering methods, biclustering algorithms discover local co-expression patterns (groups of genes/metabolites that show similar patterns under a specific subset of the experimental conditions) (Madeira and Oliveira, 2004). We manually inspected our biclustering data only in a few exemplar cases.

##### Soft clustering

Soft clustering of the metabolite and transcript differentials was performed by using the R/Bioconductor Mfuzz package (Kumar and Futschik, 2007) with the default value 1.25 for the fuzzy parameter m. A membership value in the range of 0–1 was assigned to each metabolite and probe. Soft clustering offers several advantages with respect to hard clustering; in particular, it has been suggested to be more suitable for time course microarray data in which expression patterns are often not well separated (Futschik and Carlisle, 2005; Kumar and Futschik, 2007). The biological significance of the clusters was analyzed by enrichment analysis of the MapMan functional categories assigned to the probes in each cluster. Specifically, Chi square and Fisher statistical tests were employed to search for significant differences (*p*-value < 0.05) between the observed number of probes within each MapMan functional category per cluster and the expected number of probes in that category based on the overall AROS genome array expression distribution.

##### Selection of candidate genes

From the whole set of transcripts with a potential association to monoterpenes (based on their correlation with metabolites and membership to soft clusters/biclusters harboring metabolites) we selected a subset of genes with significant expression changes and/or supporting evidence from the literature, like a relevant function in other plant species, co-localization with QTLs for monoterpene content and coexpression with genes involved in the terpene pathway. In particular, the QTL co-localization was stated when the V1 gene predictions fell into the 1-LOD confidence intervals of the QTLs for linalool, geraniol and nerol reported by Doligez et al. (2006) and Battilana et al. (2009) based on the analysis of different segregating progenies in 2–3 seasons (depending on the progeny). The genomic region corresponding to each QTL confidence interval was determined from the physical position of the two neighboring markers, while the V1 gene prediction physical position was retrieved from Grimplet et al. (2012).

#### Integration of transcriptomic and metabolic data over multiple seasons to verify a subset of candidate genes

For the candidate genes assessed by both microarray and real-time PCR analyses (in 2006 and 2016, respectively), the association between expression and metabolic profiles was further tested by employing a general monoterpene quantification that considers the three seasons (2005, 2006, and 2007) as replicates. To this purpose, the average concentration among these seasons was computed for each metabolite at each developmental stage. Pearson and Spearman correlations were calculated between the transcriptional and metabolic data expressed as log_2_ fold changes at the stages 2-5 (E-L 34.5, 36, 38, and 39) with respect to the first stage (E-L 31).

## Results and discussion

Our study gives an example of the systems biology approach. Systems biology has been successfully applied to the discovery of regulatory and biosynthetic genes involved in the control of metabolite production (Yuan et al., 2008; Liberman et al., 2012), including examples from grape (Zamboni et al., 2010; Fortes et al., 2011; Agudelo-Romero et al., 2013; Costantini et al., 2015; Malacarne et al., 2015; Suzuki et al., 2015; Wen et al., 2015; Savoi et al., 2016).

### Metabolic analysis

The present work provides a temporal profiling of aromatic compounds in the Moscato Bianco ripening berry. The protocol used for the chemical analysis was optimized for molecules belonging to the monoterpenoid class, however it allowed the simultaneous quantification of additional metabolites. In particular, the content of 21 monoterpenoids, 3 C_13_-norisoprenoids, 5 phenylpropanoids/benzenoids and 3 C_6_ aliphatic compounds was quantified from pre-*veraison* to over-ripening in 2005, 2006, and 2007. For several compounds a coherent accumulation trend was observed in the different years (Figure 1 and Supplementary Figure S1A). The most significant correlation between seasons was observed for free linalool, nerol, α-terpineol, hydroxy-diendiol I + hydroxy-trienol, hydroxy-diendiol II, hexanol, *cis*-3-hexen-1-ol, bound linalool, geraniol, nerol, *trans*-furan linalool oxide and benzyl alcohol. Other metabolites appeared instead to be more sensitive to seasonal effects, like rainfall and temperature (Supplementary Figure S1B). Hereafter, we will refer to 2006, which is the year assayed by microarray analysis.

The most abundant metabolites were monoterpenes (hydroxy-diendiol I, *trans*-geranic acid, linalool, geraniol and nerol), with concentrations higher than 600 μg/kg of berries (Figure 2 and Supplementary Figure S2). The majority of monoterpenoids reached the highest amount in their glycosidically bound form. The main exceptions are represented by high oxidation state monoterpenes, like the two pyran linalool oxides, the two diendiols and rose oxide I. A clear prevalence of the free form was also observed for the C_6_ aliphatic compounds, while the most abundant C_13_-norisoprenoids and phenylpropanoids/benzenoids were glycosidically bound, in agreement with previous analyses (Sánchez Palomo et al., 2006; D'Onofrio et al., 2016).

The quantity of many metabolites was significantly (at least 2-fold) altered during ripening. The compounds that changed most with respect to pre-*veraison* were linalool, geraniol, nerol, cis/*trans*-8-hydroxy-linalool, hydroxy-diendiol I and II in both forms; *cis*-furan linalool oxide, *trans*-geranic acid, 7-hydroxy-geraniol, 7-hydroxy-citronellol, hydroxy-trienol in their free form; *trans-*furan linalool oxide, 3-oxo-α-ionol, methyl salicylate, hexanol and *cis*-3-hexen-1-ol in their bound form (Supplementary Figures S3A,D, Supplementary Table S4).

The pattern of accumulation along berry development varied with the metabolite (Figure 2 and Supplementary Figure S2). The concentration of the three compounds mainly contributing to Muscat aroma (linalool, geraniol, and nerol) was from low to moderate before *veraison* (August 17 or stage E-L 34.5 in this work) and then increased during ripening. Free linalool reached its maximum on September 13 (technological maturity or stage E-L 38) and decreased during over-ripening. A similar behavior was observed in 2005 and 2007, even though the peak corresponded to slightly earlier stages (Figure 1E), proving that technological and aroma ripening might not occur at the same time (Vilanova et al., 2012). Otherwise, free geraniol and nerol as well as the three bound forms showed a steady increase in their content during the sampling period. These results confirm previous findings (Günata et al., 1985; Ebang-Oke et al., 2003; Piazzolla et al., 2016). Several additional patterns were observed. For example, the four linalool oxides could be detected at berry onset; their concentration reached a minimum between July 31 (stage E-L 31.5 in this work) and August 8 (stage E-L 33) and then increased in at least one of the two forms to peak on September 13 (stage E-L 38) in their free form. While the glycosidically bound forms of the two diendiols showed a similar pattern of accumulation, free hydroxy-diendiol I and hydroxy-trienol were highly concentrated before *veraison* and decreased over the course of berry ripening, with a trend opposite to that of free hydroxy-diendiol II (Figures 1, 2). The high content of free hydroxy-diendiol I and hydroxy-trienol at berry onset, when free linalool was not yet produced, may indicate that their accumulation is regulated independently from that of their precursor.

**Figure 3:**
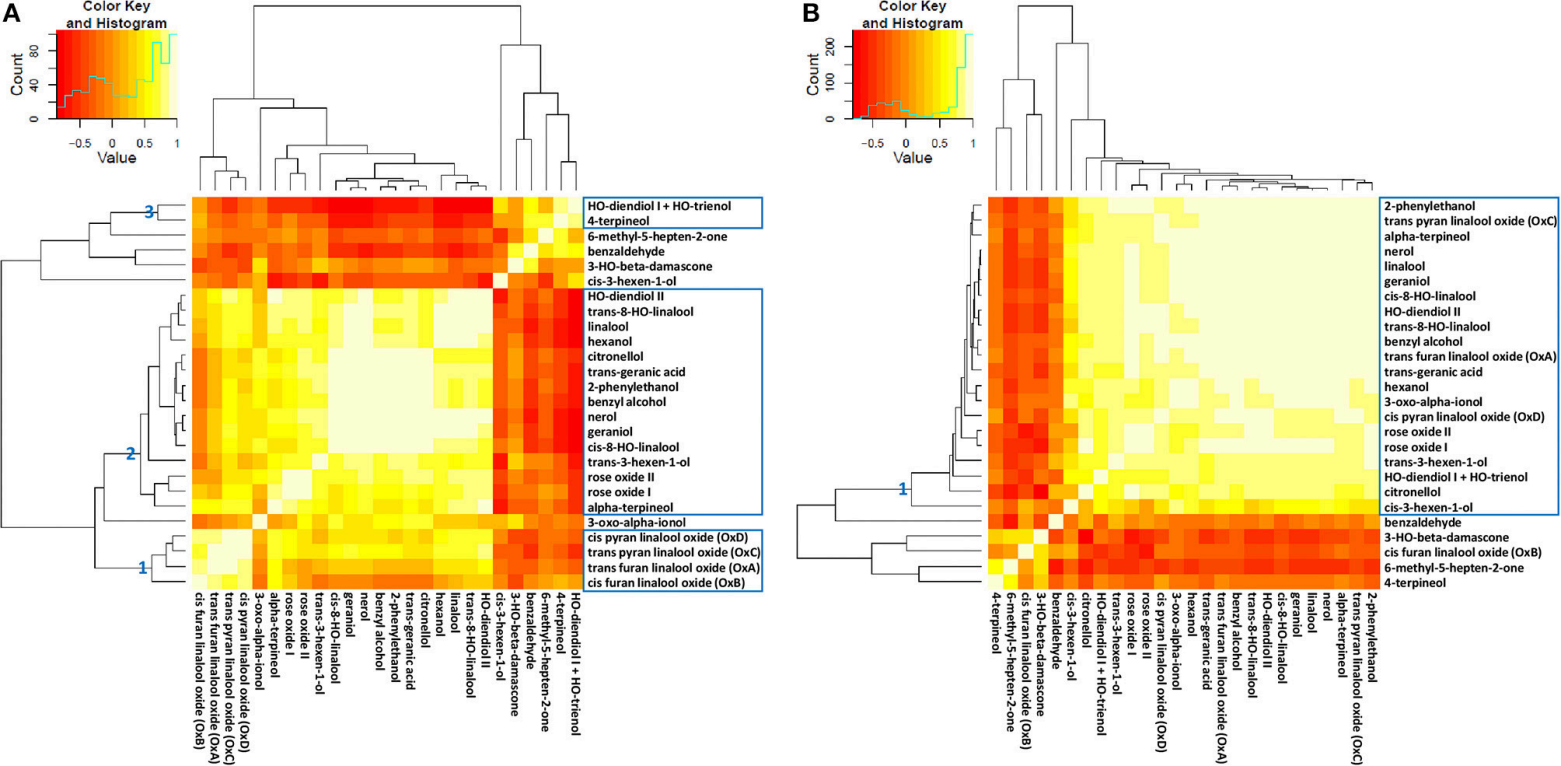
**Correlation heatmap of free (A)** and bound **(B)** log_2_-transformed metabolites analyzed at 13 time points in 2006. The dendrograms are the output of Pvclust clustering (for details see Supplementary Figures S4A,C). The intensity of yellow coloration indicates the strength of relationships between metabolites.

In the attempt of simplification, metabolite network analysis was performed on a total of 52 (26 free and 26 glycosidically bound) compounds. Metabolite grouping was obtained through hierarchical clustering and principal component analysis by using different metabolic data sets (Figure 3 and Supplementary Figures S3, S4). It is clearly evident that most monoterpenes are tightly correlated, which is indicative of their common metabolic origin and in agreement with previous findings (Ilc et al., 2016b). In particular, when considering the absolute amount of free metabolites at 13 time points, three main clusters (AU > 95%) could be identified: (1) *cis*-pyran linalool oxide (OxD), *trans*-pyran linalool oxide (OxC), *trans*-furan linalool oxide (OxA) and *cis*-furan linalool oxide (OxB); (2) geraniol, nerol, *cis*-8-hydroxy-linalool, benzyl alcohol, 2-phenylethanol, *trans*-geranic acid, citronellol, hydroxy-diendiol II, *trans*-8-hydroxy-linalool, linalool, hexanol, *trans*-3-hexen-1-ol, rose oxide I, rose oxide II and α-terpineol; (3) 4-terpineol and hydroxy-diendiol I + hydroxy-trienol. Additionally, within the second cluster a clear separation could be noticed between linalool on one side, geraniol and nerol on the other side (Figure 3A and Supplementary Figure S4A). Oppositely, when considering the absolute amount of bound metabolites at 13 time points a single significant cluster was obtained, which included most of the analyzed compounds. It can be easily noticed that bound *cis*-furan linalool oxide (OxB) has a peculiar behavior with respect to the other three linalool oxides (Figure 3B and Supplementary Figure S4C).

### Microarray analysis

Gene expression in Moscato Bianco berry skin at stages 2–5 was compared to stage 1 (pre-*veraison*). The two biological replicates assayed at each stage were confirmed to perfectly cluster together (Supplementary Figure S5). The total number of differentially expressed probes (DEPs) in at least one comparison was 2,228, which corresponds to 15% of the chip probes (Supplementary Table S5). As expected, the highest number of DEPs was observed in stage 5 vs. stage 1 (616 up-regulated and 1,132 down-regulated probes), whereas the lowest number was recorded in stage 2 vs. stage 1 (452 up-regulated and 506 down-regulated probes). A number of DEPs were common among comparisons (21, 19, and 28% of common DEPs among 2, 3, and 4 comparisons, respectively), whereas 32% of the DEPs were regulated at only one time point (data calculated from Supplementary Table S5).

### Microarray validation via real-time PCR

**Figure 4:**
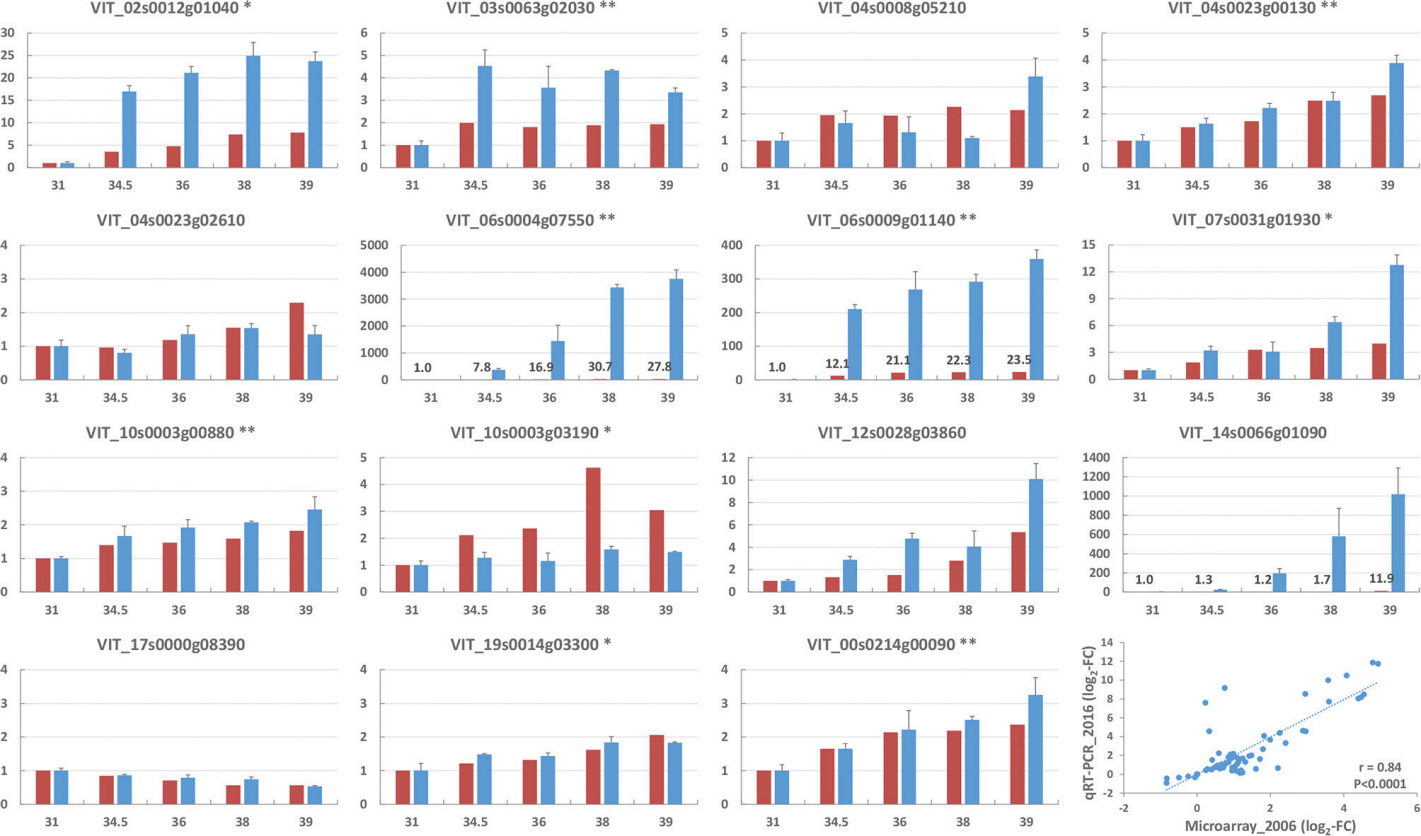
**Relative expression of the 15 genes reported in Supplementary Table S3 as assayed by microarray (red, year 2006) and quantitative real-time RT-PCR (blue, year 2016) analyses in the skin of Moscato Bianco berries sampled at 5 growth stages from the E-L modified system (Coombe, 1995; x-axis)**. The expression of the E-L stage 31 was set up as 1. The y-axis indicates the folds of gene expression relative to the E-L stage 31. The genes showing significant agreement between microarray and qRT-PCR data are marked with stars based on Pearson correlation coefficient in 0.01 (two stars) or 0.05 (one star). Quantitative real-time RT-PCR data are presented as means ± standard errors of three biological and two technical replicates. A scatterplot of the correlation between the fold changes (log_2_) in the expression levels of the 15 genes obtained by microarray and qRT-PCR analyses is shown in the last chart.

Specific primers were designed for 15 candidate genes and the change in their expression during berry development was analyzed in skin tissues by qRT-PCR to validate the microarray dataset (Figure 4). A strong relationship was found between the microarray and qPCR fold changes in the expression levels of the 15 genes (overall Pearson correlation coefficient *r* = 0.84, with individual values ranging from 0.47 to 1), indicating the reliability of the whole transcriptome assay (Figure 4 and Supplementary Table S3).

### Discovery of a link between transcriptome and metabolome in aroma development

Potential links between transcripts and metabolites were established based on correlation and clustering approaches, though they do not necessarily imply causation. To this purpose, 4,450 probes with adjusted *p*-values < 0.05 in all comparisons were considered, which included 1,906 out of the 2,228 DEPs with a cut-off of 2 fold-change and adjusted *p*-value < 0.01.

#### Integration of 2006 transcriptomic and metabolic data

##### Pairwise correlation

Significant (at the 0.05 or 0.01 level) Pearson correlations could be established only in the absence of Benjamini and Hochberg (1995) correction for multiple testing (Supplementary Table S6). Consequently, this result was employed as criterium in the following candidate gene selection only in combination with additional supporting evidence. In the two-step strategy, positive pairwise expression correlations were discovered between 33 enzyme/transporter-coding genes correlated to metabolites and several regulatory genes within the microarray (Supplementary Table S6).

##### Correlation biclustering

Correlation clustering between transcripts and metabolites resulted in the identification of 419 biclusters, that are groups of probes with a common behavior toward a group of metabolites. The clustered probes and metabolites were found to belong to a number of biclusters ranging from 1 to 10 and from 3 to 182, respectively (Supplementary Table S7).

##### Soft clustering

**Figure 5:**
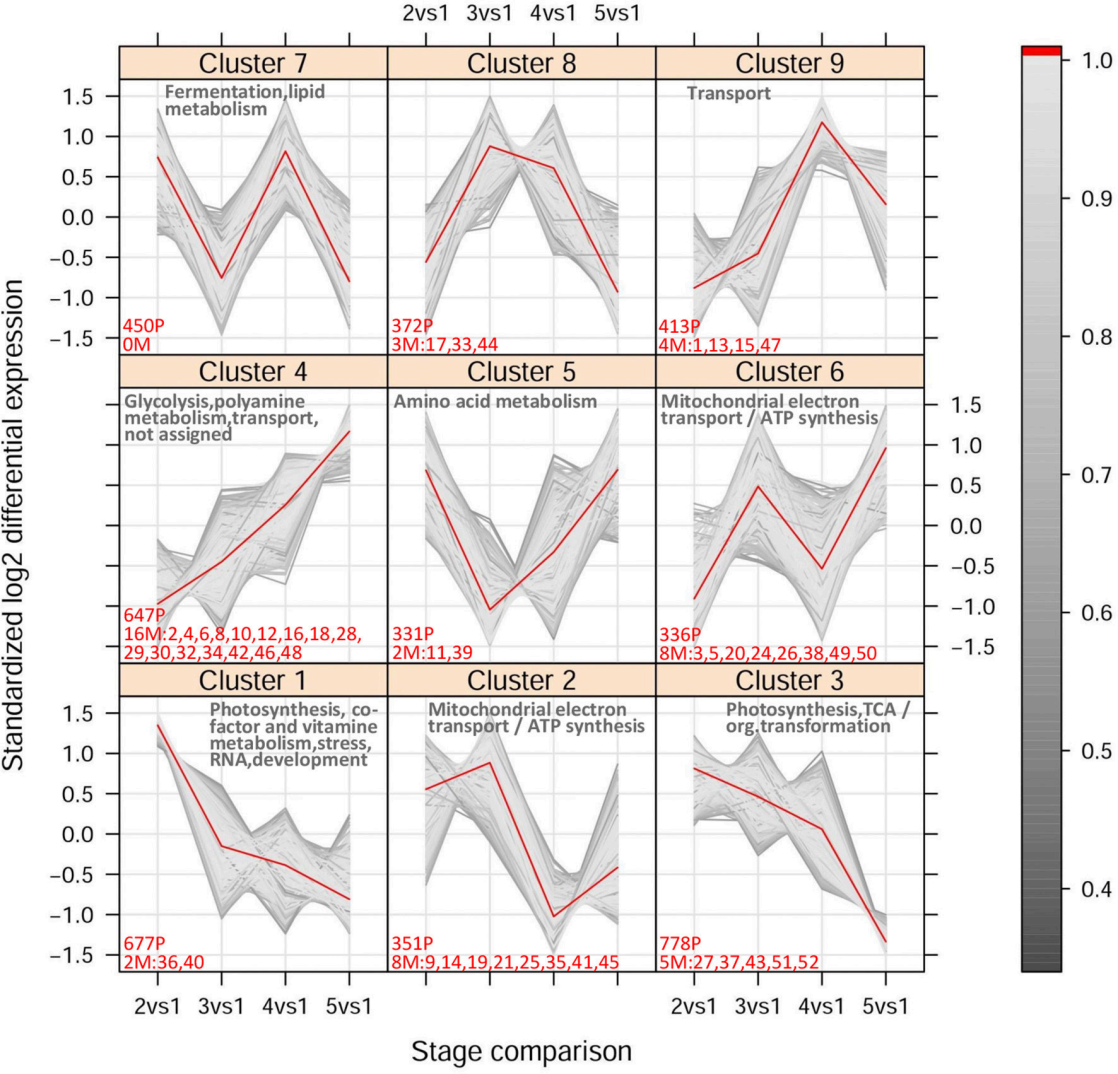
**Fuzzy c-means cluster output**. The expression levels are represented as fold changes relative to stage 1 (pre-*veraison*). The time scale corresponds to the pairwise comparison between stages 2–5 and stage 1 in 2006. The color legend shows the cluster membership values calculated as described in Futschik and Carlisle (2005) and Kumar and Futschik (2007). The number of probes (P) and metabolites (M) included within each cluster (membership > 0.5) is indicated in red. The names of metabolites are abbreviated as follows: 1 and 2 = free and bound linalool, 3 and 4 = free and bound geraniol, 5 and 6 = free and bound nerol, 7 and 8 = free and bound *trans*-8-hydroxy-linalool, 9 and 10 = free and bound *cis*-8-hydroxy-linalool, 11 and 12 = free and bound *trans*-furan linalool oxide (OxA), 13 and 14 = free and bound *cis*-furan linalool oxide (OxB), 15 and 16 = free and bound *trans*-pyran linalool oxide (OxC), 17 and 18 = free and bound *cis*-pyran linalool oxide (OxD), 19 and 20 = free and bound *trans*-geranic acid, 21 and 22 = free and bound citronellol, 23 and 24 = free and bound α-terpineol, 25 and 26 = free and bound 4-terpineol, 27 and 28 = free and bound hydroxy-diendiol I + hydroxy-trienol, 29 and 30 = free and bound hydroxy-diendiol II, 31 and 32 = free and bound rose oxide I (*cis* isomer), 33 and 34 = free and bound rose oxide II (*trans* isomer), 35 and 36 = free and bound 3-hydroxy-β-damascone, 37 and 38 = free and bound 3-oxo-α-ionol, 39 and 40 = free and bound 6-methyl-5-hepten-2-one, 41 and 42 = free and bound benzyl alcohol, 43 and 44 = free and bound benzaldehyde, 45 and 46 = free and bound 2-phenylethanol, 47 and 48 = free and bound hexanol, 49 and 50 = free and bound *trans*-3-hexen-1-ol, 51 and 52 = free and bound *cis*-3-hexen-1-ol. A summary of the Mapman functional categories over-represented within each cluster is also reported.

Based on their expression profile across stages 1–5, the selected 4,450 probes and 52 metabolites were clustered into nine distinct Mfuzz groups (Figure 5 and Supplementary Table S8). The distribution of probes per cluster within each MapMan functional category is shown in Supplementary Figure S6 and the enriched categories within each cluster are indicated in Figure 5. Probes annotated with the Mapman functional category “Secondary metabolism” were not found to be significantly over-represented in any cluster. Free geraniol and nerol were attributed to a distinct cluster (cluster 6) with respect to free linalool (cluster 9), which reflects their Pvclust clustering (Figure 3A and Supplementary Figure S4A). This separation is mainly due to the decrease of free linalool, but not geraniol and nerol, from technological maturity onwards (Figure 2). The highly similar accumulation trend of geraniol and nerol likely reflects a common chemical origin (nerol is a geometrical isomer of geraniol), while their relationship with linalool is less clear. Oppositely, the bound forms of the three monoterpenoids accumulated to a similar extent (cluster 4 in Figure 5), suggesting dynamic changes in the distribution and concentration of these compounds.

#### Selection of candidate genes

Several genes with a potential association to aroma-related compounds were identified from the probes correlated and clustered with those metabolites (Supplementary Table S9, Supplementary Discussion in Supplementary Data1 and Supplementary Table S10). In particular, the contrasting behavior of free linalool and free geraniol/nerol encouraged us to search for genes specifically related to one or the other profile. The existence of linalool-specific metabolic pathways is even more intriguing if we consider that the prevalence of the linalool class on the geraniol one clearly distinguishes Moscato Bianco from other aromatic varieties (D'Onofrio et al., 2016).

From this broad gene set, the most promising candidates for monoterpene biosynthesis and its regulation were further selected (Table 2) based on supporting evidence from the literature, e.g., a relevant role for the homolog gene in other plant species, the co-localization with QTLs for monoterpene content (with a special attention to the linalool-specific QTLs on chromosomes 2 and 10, which were also detected in the Moscato Bianco genetic background by Battilana et al., 2009), or the coexpression from public transcriptomic databases with genes involved in the metabolic pahways under study, which may indicate functional association according to the “guilt-by-association” principle. Hereafter, we discuss the most interesting findings from the present work; obvioulsy, we can't exclude that additional genes not included in this microarray platform may participate in monoterpene biosynthesis, as well as we can't know *a priori* whether our findings will be reproduced in other Muscat varieties with a genetic background different from Moscato Bianco.

**Table 2:** **Summary of the most significant candidate genes for monoterpene biosynthesis**.

**Non-redundant V1 gene prediction ID**	**Functional annotation**	**Evidences from the present work**	**Evidences from the literature**
**Cluster 4 (free HO-diendiol II, bound linalool, geraniol, nerol**, ***trans*****/*****cis*****-8-HO-linalool, OxA, OxC, OxD, HO-diendiol I** + **HO-trienol, HO-diendiol II, rose oxide I and II)**			
VIT_01s0026g01970	RNA-binding region RNP-1 (RNA recognition motif)	Correlated with VIT_02s0012g01630, VIT_05s0062g00430, VIT_05s0062g00520 and additional candidate genes in Supplementary Table S9 Down-regulated at stages 2–5 with respect to stage 1	§ Ruwe et al., 2011 # Battilana et al., 2009
VIT_02s0012g01040^*^	NAC domain-containing protein 71	Correlated with VIT_06s0009g01140 and an additional candidate gene in Supplementary Table S9 Correlated with free OxC, bound linalool, *trans*-8-HO linalool, OxA, HO-diendiol I and II, rose oxide I and II Up-regulated at stages 2-5 with respect to stage 1 (array and qRT-PCR)	# Doligez et al., 2006; Battilana et al., 2009; Lijavetzky et al., 2012; Palumbo et al., 2014
VIT_02s0234g00100	Ubiquitinyl hydrolase 1	Correlated with VIT_08s0007g05210, VIT_10s0003g00880 and additional candidate genes in Supplementary Table S9 Correlated with several bound monoterpenes Up-regulated at stage 5 with respect to stage 1	-
VIT_04s0023g00130^*^	Unknown protein	Positive correlation with free OxC, bound OxA, HO-diendiol I, rose oxide I and II Up-regulated at stages 4 and 5 with respect to stage 1 (array), at stage 5 with respect to stage 1 (qRT-PCR)	# Battilana et al., 2009; Lijavetzky et al., 2012; Palumbo et al., 2014
VIT_04s0023g02950	Zinc finger (CCCH-type) family protein	Correlated with VIT_02s0012g01630, VIT_05s0062g00430, VIT_05s0062g00520 and additional candidate genes in Supplementary Table S9 Correlated with bound OxC Down-regulated at stages 2–4 with respect to stage 1	# Battilana et al., 2009; Lijavetzky et al., 2012
VIT_06s0009g01140^*^	Amino acid permease	Correlated with free linalool, *trans*-8-HO-linalool, α-terpineol, HO-diendiol II, rose oxide I, bound linalool, geraniol, nerol, *cis*-8-HO-linalool, citronellol Up-regulated at stages 2-5 with respect to stage 1 (array and qRT-PCR)	# Battilana et al., 2009; Palumbo et al., 2014; VTCdb
VIT_07s0031g01320	TGA-type basic leucine zipper protein TGA1.1	Correlated with VIT_02s0012g01630, VIT_05s0062g00430, VIT_05s0062g00520, VIT_00s0389g00030, VIT_00s0389g00040 and additional candidate genes in Supplementary Table S9 Correlated with bound *trans*-8-HO-linalool, OxD, HO-diendiol II Down-regulated at stages 2-5 with respect to stage 1	# Cramer et al., 2014; Wen et al., 2015
VIT_07s0031g01930^*^	Myb TKI1 (TSL-kinase interacting protein 1)	Correlated with VIT_06s0009g01140 and additional candidate genes in Supplementary Table S9 Correlated with several monoterpenes (free linalool with high significance) Up-regulated at stages 2-5 with respect to stage 1 (array), at stages 4 and 5 with respect to stage 1 (qRT-PCR)	# Lijavetzky et al., 2012; Palumbo et al., 2014
VIT_08s0040g03040	Glutathione S-transferase GSTO1	Correlated with bound *trans*-8-HO-linalool, OxD, *trans*-geranic acid, α-terpineol, HO-diendiol II Up-regulated at stage 5 with respect to stage 1	# VTCdb
VIT_09s0054g01780	HAC1 (P300/CBP acetyltransferase-related protein 2 gene)	Correlated with VIT_04s0023g02610 Correlated with bound *trans*-8-HO-linalool, rose oxide II Up-regulated at stages 3-5 with respect to stage 1	§ (Shen et al., 2015)
VIT_10s0003g00880^*^	Nudix hydrolase 15	Correlated with bound *trans*-8-HO-linalool and OxC Up-regulated at stage 4 with respect to stage 1 (qRT-PCR)	§ Magnard et al., 2015
VIT_12s0028g03860^*^	Zinc finger (C3HC4-type ring finger) protein (RMA1)	Correlated with VIT_10s0003g00880 Correlated with bound OxC Up-regulated at stages 4 and 5 with respect to stage 1 (array), at stage 2 with respect to stage 1 (qRT-PCR)	# Battilana et al., 2009; Lijavetzky et al., 2012; Palumbo et al., 2014; VTCdb
VIT_14s0066g01090^*^	Myb domain protein 24	Correlated with VIT_18s0001g04280 and VIT_18s0001g04530 Correlated with bound OxC Up-regulated at stage 5 with respect to stage 1 (array)	# Battilana et al., 2009; Carbonell-Bejerano et al., 2014b; Savoi et al., 2016; Wong et al., 2016
VIT_18s0001g13790	Cytochrome P450, family 83, subfamily B, polypeptide 1 (CYP71)	Correlated with several oxidized monoterpenes (bound *cis*/*trans* 8-HO-linalool, OxA, OxD, *trans*-geranic acid, HO-diendiol I and II) In the same biclusters as free *cis*-8-HO-linalool, OxB, *trans*-geranic acid, HO-diendiol I + HO-trienol Up-regulated at stages 3-5 with respect to stage 1	§ Ginglinger et al., 2013 # Ilc et al., 2017
VIT_19s0014g03300^*^	NAC domain containing protein 2	Correlated with VIT_04s0023g02610 and VIT_10s0003g00880 Correlated with bound *trans*-8-HO-linalool, OxC, rose oxide II Up-regulated at stage 5 with respect to stage 1 (array)	§ Giovannoni, 2004; Nieuwenhuizen et al., 2015 # Lijavetzky et al., 2012; Blanco-Ulate et al., 2015
VIT_00s0214g00090^*^	F-box protein PP2-B10 (Protein phloem protein 2-like B10)	Correlated with VIT_06s0009g01140, VIT_15s0048g01490 and additional candidate genes in Supplementary Table S9 Correlated with several monoterpenes Up-regulated at stages 3–5 with respect to stage 1 (array), at stage 4 with respect to stage 1 (qRT-PCR)	# Battilana et al., 2009; Palumbo et al., 2014
**Cluster 6 (free geraniol, nerol, bound** ***trans*****-geranic acid**, α**-terpineol, 4-terpineol)**			
VIT_02s0012g01240	PHD finger transcription factor	Correlated with VIT_06s0009g01140 and VIT_08s0007g05210 Correlated with free geraniol, bound α-terpineol Up-regulated at stage 5 with respect to stage 1	# Doligez et al., 2006; Battilana et al., 2009
VIT_08s0007g05210	Amino acid permease	Correlated with free geraniol and bound α-terpineol Up-regulated at stage 5 with respect to stage 1	# Battilana et al., 2009; VTCdb
VIT_15s0048g02410	Myb CCA1 (circadian clock associated 1)	Down-regulated at stage 4 with respect to stage 1	§ Vranová et al., 2012 # VTCdb
**Cluster 9 (free linalool, OxB, OxC)**			
VIT_02s0012g01630	Transmembrane protein 41B (SNARE associated Golgi protein)	Down-regulated at stages 2-5 with respect to stage 1	§ Ting et al., 2015 # Doligez et al., 2006; Battilana et al., 2009
VIT_04s0008g05210^*^	BZIP protein HY5 (HY5)	Correlated with VIT_06s0009g01140 Up-regulated at stages 4 and 5 with respect to stage 1 (array)	§ Liu et al., 2004; Toledo-Ortiz et al., 2014; Zhou et al., 2015 # Carbonell-Bejerano et al., 2014a,b; Cramer et al., 2014; Liu et al., 2015; Loyola et al., 2016
VIT_04s0023g01250	Brassinosteroid signaling positive regulator (BZR1)	Correlated with VIT_02s0012g01630, VIT_00s0389g00030, VIT_00s0389g00040 and additional candidate genes in Supplementary Table S9 Correlated with free OxB Down-regulated at stage 2, 3 and 5 with respect to stage 1	§ Liu et al., 2014 # Battilana et al., 2009; Fortes et al., 2015
VIT_06s0004g07550^*^	Wound-induced protein WI12	Correlated with VIT_06s0009g01140 and an additional candidate gene in Supplementary Table S9 Correlated with free OxC, HO-diendiol II, bound linalool, geraniol, nerol, OxA, citronellol, HO-diendiol I, rose oxide I Up-regulated at stages 2-5 with respect to stage 1 (array), at stages 2, 4 and 5 with respect to stage 1 (qRT-PCR)	# Battilana et al., 2009; Lijavetzky et al., 2012; Palumbo et al., 2014; VTCdb
VIT_07s0104g01050	Homeobox protein	Correlated with VIT_06s0009g01140 and an additional candidate gene in Supplementary Table S9 Correlated with several monoterpenes Up-regulated at stages 4 and 5 with respect to stage 1	-
VIT_08s0007g05880	Dehydration-induced protein (ERD15)	Correlated with VIT_02s0012g01630, VIT_05s0062g00430, VIT_05s0062g00520, VIT_15s0048g01590, VIT_00s0389g00030, VIT_00s0389g00040 and additional candidate genes in Supplementary Table S9 Down-regulated at stages 2-5 with respect to stage 1	# Koundouras et al., 2006; Battilana et al., 2009; Savoi et al., 2016
**Other clusters**			
VIT_10s0003g03190^*^	RNA recognition motif (RRM)-containing	Correlated with VIT_06s0009g01140 and an additional candidate gene in Supplementary Table S9 Up-regulated at stages 2–5 with respect to stage 1 (array)	# Battilana et al., 2009; Lijavetzky et al., 2012; VTCdb
VIT_16s0100g00400	Ethylene-responsive transcription factor ERF025	Correlated with VIT_02s0012g01630, VIT_05s0062g00430, VIT_05s0062g00520, VIT_00s0389g00030, VIT_00s0389g00040 and additional candidate genes in Supplementary Table S9 Down-regulated at stages 3-5 with respect to stage 1	# Palumbo et al., 2014; VTCdb
VIT_18s0001g05250	DREB sub A-6 of ERF/AP2 transcription factor (RAP2.4)	Correlated with VIT_02s0012g01630, VIT_05s0062g00430, VIT_05s0062g00520, VIT_15s0048g01590, VIT_00s0389g00030, VIT_00s0389g00040 and additional candidate genes in Supplementary Table S9 Down-regulated at stages 2-5 with respect to stage 1	# Wen et al., 2015
VIT_03s0038g02500	SKP1	Correlated with VIT_04s0023g02610 and VIT_10s0003g00880 Up-regulated at stage 5 with respect to stage 1	§ Devoto et al., 2002 # D'Onofrio et al., 2009; Gómez-Plaza et al., 2012; May and Wüst, 2015
VIT_03s0063g02030^*^	4-hydroxy-3-methylbut-2-enyl diphosphate reductase	In the same cluster as free OxA Up-regulated at stages 2, 4 and 5 with respect to stage 1 (qRT-PCR)	§ Botella-Pavía et al., 2004 # Martin et al., 2012; Wen et al., 2015
VIT_03s0180g00200 VIT_03s0180g00320	Limonoid UDP-glucosyltransferase Indole-3-acetate beta-glucosyltransferase	In the same biclusters as bound OxC, 4-terpineol, HO-diendiol I + HO-trienol Down-regulated at stage 3 with respect to stage 1	§ Kita et al., 2000 # Bönisch et al., 2014a; Blanco-Ulate et al., 2015
VIT_04s0023g02610^*^	Epoxide hydrolase 2	Up-regulated at stage 5 with respect to stage 1 (array)	# Battilana et al., 2009; Savoi et al., 2016
VIT_15s0046g01440	BZip transcription factor G- box binding factor 3	Negatively correlated with several monoterpenes Down-regulated at stages 3–5 with respect to stage 1	§ Sibéril et al., 2001 # Cramer et al., 2014
VIT_15s0048g01490	Geraniol 10-hydroxylase (CYP76)	In the same cluster as free OxD and rose oxide II In the same biclusters as free HO-diendiol I+ HO-trienol, bound OxC, HO-diendiol I + HO-trienol Up-regulated at stages 3 and 4 with respect to stage 1	§ Ginglinger et al., 2013; Höfer et al., 2014; Boachon et al., 2015 # Cramer et al., 2014; Ilc et al., 2017
VIT_15s0048g01590	CYP76B1	In the same biclusters as free OxA Down-regulated at stage 3 with respect to stage 1	§ Ginglinger et al., 2013; Höfer et al., 2014; Boachon et al., 2015 # Ilc et al., 2017; VTCdb
VIT_16s0039g00010	ABC transporter G member 7	Up-regulated at stage 5 with respect to stage 1	§ Kang et al., 2011 # Cramer et al., 2014; VTCdb
VIT_16s0050g01580	UDP-glucose: anthocyanidin 5,3-O-glucosyltransferase (*VvGT7*)	In the same biclusters as bound HO-diendiol I + HO-trienol Down-regulated at stages 3–5 with respect to stage 1	§ Caputi et al., 2008 # Doligez et al., 2006; Bönisch et al., 2014a; Friedel et al., 2016
VIT_18s0001g04280 VIT_18s0001g04530	(-)-germacrene D synthase	Up-regulated at stage 5 with respect to stage 1	# Savoi et al., 2016
VIT_18s0001g09230	Salt tolerance zinc finger	Negatively correlated with bound 4-terpineol Down-regulated at stage 5 with respect to stage 1	§ Pauw et al., 2004 # Cramer et al., 2014
VIT_00s0389g00030 VIT_00s0389g00040	CYP72A1	In the same clusters as free OxD, HO-diendiol I + HO-trienol, rose oxide II In the same biclusters as free HO-diendiol I + HO-trienol, bound OxC Down-regulated at stage 5 with respect to stage 1	# Doligez et al., 2006; Battilana et al., 2009; Cramer et al., 2014; VTCdb
VIT_00s0463g00020	Scarecrow transcription factor 5 (SCL5)	Correlated with VIT_02s0012g01630, VIT_00s0389g00030, VIT_00s0389g00040 and additional candidate genes in Supplementary Table S9 Down-regulated at stages 2–5 with respect to stage 1	# Battilana et al., 2009; Wen et al., 2015

*They were selected from the whole list of genes with a potential association to monoterpenes (based on their correlation with metabolites and/or membership to soft clusters/biclusters harboring metabolites) reported in Supplementary Table S9, giving priority to the genes with the biggest expression changes, supporting evidence from the literature (relevant function in other plant species, co-localization with QTLs for monoterpene content, coexpression with genes involved in the terpene pahway), profile consistency between microarray and qRT-PCR analyses (the genes assayed by both techniques are marked with an asterisk). The symbols # and § indicate references for grapevine and other plant species, respectively. For details, including the correspondence between microarray probes and V1 gene predictions, see Supplementary Table S9*.

##### Monoterpene skeleton biosynthesis

*Early terpenoid pathway genes*. The role of *VvDXS* isoforms in the development of aroma was previously investigated by real-time PCR on the same samples of Moscato Bianco analyzed here (Battilana, 2009), for which reason we did not repeat the analysis. In that study a significant up-regulation of *VvDXS1* was found to precede the peak of linalool, geraniol and nerol resulting in a positive correlation between *VvDXS1* expression profile and monoterpenoid accumulation. On the Grape AROS V1.0 array no probe could be found for *VvDXS1*, whereas four probes corresponding to other *DXS* isoforms (VIT_04s0008g04970 and VIT_00s0218g00110) were not differentially expressed during Moscato Bianco berry ripening.

Several pieces of evidence from different plant species suggest that flux control in the MEP pathway does not converge on a single rate-limiting enzyme, such as DXS, but may involve other enzymes like DXR (1-deoxy-D-xylulose 5-phosphate reductoisomerase) and HDR (Vranová et al., 2012; Hemmerlin, 2013). The lack of significant modulation and the decreasing trend during berry ripening observed for *VvDXR* in our study (VIT_17s0000g08390 in Figure 4) do not support a regulatory role, in agreement with Rodríguez-Concepción et al. (2001) and Mendoza-Poudereux et al. (2014). Oppositely, the expression of *VvHDR* (VIT_03s0063g02030 in Figure 4) is consistent with the *veraison*-initiated accumulation of monoterpenes, as reported by Martin et al. (2012) and Wen et al. (2015) (Table 2 and Supplementary Table S9).

*Middle and late terpenoid pathway genes*. In other plant species GPPS works as a heterodimeric complex; in particular, the levels of GPPS small subunit, but not GPPS large subunit, might play a key role in regulating monoterpene biosynthesis (Tholl et al., 2004). Consistently, the AROS probes for GPPS large subunit genes (VIT_04s0023g01210 and VIT_18s0001g12000) were neither differentially expressed during Moscato Bianco berry ripening nor correlated to any monoterpene. No probe could be identified for the GPPS small subunit.

Only three probes for terpene synthases are present on the Grape AROS V.1 array, which are not specific to any single gene prediction. One of them, showing the best match to the sesquiterpene synthases VIT_18s0001g04280 and VIT_18s0001g04530, was up-regulated during Moscato Bianco berry ripening (Table 2 and Supplementary Table S9). It is worth noting that the same genes were reported to correlate with linalool and α-terpineol (Savoi et al., 2016).

An interesting candidate gene for the biosynthesis of monoterpenes is a nudix hydrolase (VIT_10s0003g00880), whose expression increases along berry development (Figure 4). The corresponding probe belongs to cluster 4, which also harbors several monoterpenes (Table 2, Supplementary Tables S8, S9). Recently, a rose nudix hydrolase was reported to convert geranyl diphosphate to geranyl monophosphate, which in turn is hydrolyzed to geraniol by a phosphatase activity (Magnard et al., 2015). This alternative and completely new terpene synthase-independent route for monoterpene production might play a role also in other plants, including grapevine.

##### Secondary monoterpene transformations

Extensive oxidative monoterpene metabolism has been reported in grapes and wine, with a percentage of linalool oxygenation ranging from 52 to 97% (Ilc et al., 2016b). The main linalool oxidation products are *trans*/*cis*-8-hydroxy-linalool (by hydroxylation), *trans*/*cis* pyranoid/furanoid linalool oxides and polyhydroxylated derivatives or polyols like the odorless hydroxy-diendiol I and II (by epoxidation and hydrolysis). Similarly, C-8 oxygenated geraniol and citronellol derivatives can be formed through hydroxylation, whereas the oxidation to geranial and neral (altogether named citral) is supposedly mediated by alcohol dehydrogenases. Geranic acid is another oxidation product of geraniol. Rose oxide is generated from citronellol by allylic hydroxylation and acid-catalyzed cyclization. Citronellol in turn arises from the reduction of geraniol and nerol (hydrogenation).

Members of the cytochrome P450 (CYP) 71 and 76 families were recently shown to metabolize linalool in *Arabidopsis thaliana* (Ginglinger et al., 2013; Höfer et al., 2014; Boachon et al., 2015). Interestingly, the CYP76 gene family has encountered an evident expansion in the grape genome (Nelson et al., 2008). In order to identify genes potentially implicated in grape monoterpenoid metabolism we looked in VTCdb database (http://vtcdb.adelaide.edu.au/Home.aspx) for CYP genes coexpressed with linalool synthases, as in Ginglinger et al. (2013). This information was then added to our transcriptomic and metabolic integrated datasets. On this base, we propose some genes (VIT_15s0048g01490, VIT_15s0048g01590, VIT_18s0001g13790, VIT_00s0389g00030, VIT_00s0389g00040) and eventually additional ones as potential CYPs involved in linalool metabolism (Table 2 and Supplementary Table S9). Most of these candidates have been never reported elsewhere, and thus deserve further attention. Conversely, VIT_15s0048g01490 and VIT_18s0001g13790 were recently characterized by Ilc et al. (2017) but their biochemical activity was only tested on a limited number of compounds. Our findings suggest instead that these genes might play a role in the production of a broader set of hydroxylated and/or epoxidized products as in other species (Meesters et al., 2007; Ginglinger et al., 2013; Höfer et al., 2013, 2014; Boachon et al., 2015) and, even if the need for further oxidoreductases can not be excluded (Ilc et al., 2016a), they encourage to check this hypothesis by analyzing additional substrates (geraniol, nerol, citronellol) and products (e. g. pyranoid/furanoid linalool oxides, hydroxy-diendiols, geranic acid, rose oxides) in CYP enzymatic assays. We also propose an epoxide hydrolase (VIT_04s0023g02610) to be assessed for involvement in monoterpene oxidative metabolism (Table 2 and Supplementary Table S9).

Based on their sequence similarity with terpenoid glucosyltransferases from different plant species and on their membership in biclusters harboring some glucosylated monoterpenes, we propose that VIT_03s0180g00200, VIT_03s0180g00320 (Table 2) and eventually other genes reported in Supplementary Table S9 (VIT_03s0091g00040, VIT_03s0180g00280, VIT_05s0062g00430, VIT_05s0062g00520, VIT_05s0062g00630, VIT_05s0062g00640) might code for enzymes that glucosylate monoterpenes along with additional metabolites. Most of these genes have been investigated in previous works but they were not considered as candidates for monoterpene glucosylation in view of their decreasing expression during berry development (Khater et al., 2011; Bönisch et al., 2014a,b). However, they might be involved in the production of glucosylated monoterpenes with a similar trend, like the high oxidation state monoterpenoids sharing the same biclusters (Table 2 and Supplementary Table S9), which were not quantified in those papers. This hypothesis is not contradicted by the lack of gene annotation referring to the “Monoterpenoid biosynthesis” pathway and of positive correlation between transcript and monoterpenyl glucoside accumulation, as the same holds for the biochemically characterized monoterpenol glucosyltransferase *VvGT7* (Table 2) and may be explained by the broad substrate tolerance and overlapping enzymatic activities of the large GT family. Monoterpenyl glucosides are only intermediates within the glycosylation pathway and post-transcriptional control is additionally involved (Bönisch et al., 2014a).

##### Monoterpene transport

Terpene transport within the cell and into the apoplast is an almost unexplored field. It may engage multiple pathways, e.g., (1) insertion of the hydrophobic terpenes into vesicle membranes followed by transport and fusion to the plasma membrane, (2) carrier proteins (like GSTs, glutathione S-transferases, and ABC, ATP-binding cassette transporters) that conduct these molecules to the (plasma) membrane, and (3) direct diffusion between the endoplasmic reticulum and/or plastidial (stromule) membranes and the plasma membrane (Ting et al., 2015). The fusion of vesicles with target membranes is mediated by a group of proteins called SNAREs (soluble NSF attachment protein receptors). Surprisingly and still without a clear underlying mechanism, both sesquiterpenes and monoterpenes were boosted when vesicle fusion was inhibited in *Nicotiana benthamiana* (Ting et al., 2015). Moreover, two *Arabidopsis* linalool synthases were detected in vesicular structures associated with the plastids (Ginglinger et al., 2013). Based on these findings, we included among our candidates a gene coding for a SNARE associated Golgi protein (VIT_02s0012g01630). Plant ABCG transporters play a role in the flux of secondary metabolites, particularly of terpenoid origin (Kang et al., 2011). Interestingly, we found an ABCG gene (VIT_16s0039g00010) that shows a profile consistent with monoterpene accumulation and is coexpressed with several monoterpene synthases in VTCdb. We also selected a glutathione S-transferase (VIT_08s0040g03040) and two amino acid permeases (VIT_06s0009g01140 and VIT_08s0007g05210), which are coexpressed with monoterpene biosynthetic genes in VTCdb and positively correlated to several monoterpenes in the present study (Table 2 and Supplementary Table S9).

##### Monoterpene biosynthesis transcriptional regulation

Recent works (Cramer et al., 2014; Wen et al., 2015) suggested that a group of ERF6-type transcription factors clustered on chromosome 16 are involved in aroma accumulation, based on the correlation of their transcript abundance and the transcript abundance of several terpenoid pathway genes. For some of these regulatory genes, e.g., the orthologs of *CrORCA2, CrORCA3*, and *AaERF1* (De Geyter et al., 2012), no probe was found among the 4,450 probes used for our integrative analysis. Other *ERF* genes reported in the mentioned papers (VIT_16s0013g00950, VIT_16s0013g00980, VIT_16s0013g00990, VIT_16s0013g01030, VIT_16s0013g01050, VIT_16s0013g01060, not listed in Supplementary Table S9) belonged to clusters 1, 2, 7 and did not show any relevant positive correlation with monoterpenes. However, some of the AROS probes had only a partial match with these genes, as a consequence they might correspond instead to *ERF* gene isoforms not involved in flavor determination. Conversely, the genes VIT_16s0100g00400 and VIT_18s0001g05250 showed an expression profile consistent with the accumulation of monoterpenes in Moscato Bianco ripening berry (Table 2 and Supplementary Table S9).

We also observed an interesting behavior (Figure 4, Table 2, and Supplementary Table S9) for TFs of the MYB (VIT_14s0066g01090) and NAC (VIT_19s0014g03300) families that promote mono- and sequiterpene production in other plant species (Reeves et al., 2012; Nieuwenhuizen et al., 2015). In particular, VIT_14s0066g01090 (*MYB24*) has been proposed as a candidate transcriptional regulator of (mono)terpene biosynthesis also in grapevine (Matus, 2016; Savoi et al., 2016), for which reason it deserves further attention. Finally, based on the negative effect of GBF1 (G-box binding factor 1) and ZCT (zinc-finger *Catharanthus* transcription factor) proteins on the expression of the TIA (terpenoid indole alkaloid) biosynthetic genes *Str* (strictosidine synthase) and *Tdc* (tryptophan decarboxylase) (Sibéril et al., 2001; Pauw et al., 2004), we selected two genes (VIT_15s0046g01440 and VIT_18s0001g09230) negatively correlated with monoterpene accumulation during Moscato Bianco berry ripening (Table 2 and Supplementary Table S9).

One of the signals dramatically impacting isoprenoid biosynthesis in higher plants is light, which activates the MEP pathway at the transcriptional and post-transcriptional level (Rodríguez-Concepción, 2006; Cordoba et al., 2009; Vranová et al., 2012; Mannen et al., 2014). Sunlight exclusion limits the synthesis and accumulation of terpenes also in grape berries (linalool and the bound forms being the most responsive) by especially affecting *DXS* and *TPS* genes (Zhang et al., 2014; Friedel et al., 2016; Joubert et al., 2016; Matus, 2016; Sasaki et al., 2016). Our findings (Figure 4, Table 2, and Supplementary Table S9) are consistent with a role, among others, for *HY5* (*LONG HYPOCOTYL5*, VIT_04s0008g05210) in the regulation of light-induced terpenoid biosynthesis in grapes, in agreement with other evidences (Carbonell-Bejerano et al., 2014a,b; Zhou et al., 2015; Loyola et al., 2016).

The isoprenoid pathway has also been reported to be under the circadian clock control. In particular, the emission of volatile terpenoids follows a diurnal rhythm and genes encoding enzymes involved in IPP biosynthesis (especially those from the MEP pathway) and downstream pathways are coexpressed with circadian clock genes and show typical circadian expression profiles (Cordoba et al., 2009; Vranová et al., 2012; Pokhilko et al., 2015). Some probes on the AROS array correspond to a gene of the circadian oscillator (VIT_15s0048g02410) and fall into clusters harboring several monoterpenes (Table 2 and Supplementary Table S9).

The expression profile of a number of additional transcription factors (including master regulators) and genes potentially involved in the post-transcriptional regulation (Hemmerlin, 2013) overlaps monoterpene accumulation during Moscato Bianco berry ripening, which supports a role in the control of monoterpene biosynthesis for VIT_01s0026g01970, VIT_02s0012g01040, VIT_02s0012g01240, VIT_02s0234g00100, VIT_03s0038g02500, VIT_04s0023g00130, VIT_04s0023g01250, VIT_04s0023g02950, VIT_06s0004g07550, VIT_07s0031g01320, VIT_07s0031g01930, VIT_07s0104g01050, VIT_08s0007g05880, VIT_09s0054g01780, VIT_10s0003g03190, VIT_12s0028g03860, VIT_00s0214g00090, VIT_00s0463g00020 (Figure 4, Table 2, Supplementary Table S9). To our knowledge, these genes represent new regulatory candidates for the production of several (cluster 4) or specific metabolites, like linalool (cluster 9) and geraniol/nerol (cluster 6), as suggested by their co-localization with QTLs and their correlation with enzyme/transporter genes correlated to metabolites.

#### Integration of transcriptomic and metabolic data over multiple seasons to verify a subset of candidate genes

In order to confirm the above links between transcriptome and metabolome in aroma development, the 15 genes assessed by both microarray and real-time analyses were also tested for correlation with the metabolic profile over three seasons, which were considered as three biological replicates (Table 3 and Supplementary Figure S1A). Significant correlations were found for all the genes except *VvDXR* (confirming the results from 2006 data) and *VvHDR*, which probably precedes monoterpene accumulation. Several compounds were affected, especially in their glycosidically bound form. Unsurprisingly, most of the metabolites with no correlation showed an inconsistent profile among seasons (e.g., free OxA and citronellol, bound α-terpineol) or a decreasing trend along berry ripening (e.g., free HO-diendiol I + HO-trienol and bound OxB). Though not ensuring a punctual conformity to the observations from a single year (Table 2), the findings from multiple seasons (Table 3) prove the general consistency of the outcomes of different techniques and years and argue for the reliability of the whole set of results based on the integration of 2006 transcriptomic and metabolic data.

**Table 3:** **Positive correlation between the expression profile of the candidate genes assessed by microarray and qRT-PCR analyses (Figure 4) and the metabolic profile over different seasons (2005, 2006, and 2007, Supplementary Figure S1A) in Moscato Bianco**.

**FREE METABOLITES**			**Linalool**	**Geraniol#**	**Nerol**	***trans*-8-HO-linalool#**	***cis*-8-HO-linalool**	**OxA**	**OxB^§^**	**OxC**	**OxD^§^**	***trans*-geranic acid#**	**Citronellol**	**α-terpineol**	**HO-diendiol I+trienol**	**HO-diendiol II**	**Rose oxide I+II^§^**
VIT_02s0012g01040	P	A	0.95^*^		0.93^*^												
		R	0.94^*^		0.94^*^							0.89^*^					
	S	A														1.00^*^	
		R														0.90^*^	
VIT_04s0008g05210	P	A	0.92^*^		0.92^*^												
		R															
	S	A															
		R															
VIT_04s0023g00130	P	A	0.89^*^													0.90^*^	
		R	0.88^*^													0.90^*^	
	S	A														1.00^*^	
		R														1.00^*^	
VIT_04s0023g02610	P	A															
		R				0.89^*^				0.92^*^				0.92^*^		0.91^*^	0.88^*^
	S	A														0.90^*^	
		R															
VIT_06s0004g07550	P	A	0.98^**^		0.96^*^							0.89^*^				0.89^*^	
		R	0.97^**^		0.96^*^							0.89^*^					
	S	A														0.90^*^	
		R														1.00^*^	
VIT_06s0009g01140	P	A	0.97^**^	0.88^*^	0.96^**^							0.91^*^					
		R	0.92^*^		0.92^*^												
	S	A														1.00^*^	
		R														1.00^*^	
VIT_07s0031g01930	P	A	0.98^**^		0.96^**^	0.90^*^										0.94^*^	
		R															
	S	A														1.00^*^	
		R														0.90^*^	
VIT_10s0003g00880	P	A	0.91^*^														
		R	0.94^*^		0.91^*^												
	S	A														1.00^*^	
		R														1.00^*^	
VIT_10s0003g03190	P	A	0.90^*^														
		R															
	S	A														0.90^*^	
		R															
VIT_12s0028g03860	P	A															
		R	0.89^*^														
	S	A														1.00^*^	
		R														0.90^*^	
VIT_14s0066g01090	P	A															
		R	0.96^**^		0.93^*^											0.94^*^	
	S	A														0.90^*^	
		R														1.00^*^	
VIT_19s0014g03300	P	A															
		R	0.90^*^														
	S	A														1.00^*^	
		R															
VIT_00s0214g00090	P	A	0.98^**^		0.97^**^							0.89^*^				0.89^*^	
		R	0.93^*^		0.90^*^											0.92^*^	
	S	A														1.00^*^	
		R														1.00^*^	
VIT_02s0012g01040	P	A	0.95^*^		0.96^*^		0.92^*^								0.99^**^		
		R			0.88^*^										0.93^*^		
	S	A	1.00^*^	0.90^*^	1.00^*^	1.00^*^	1.00^*^	0.90^*^		1.00^*^	0.90^*^	0.90^*^			1.00^*^	1.00^*^	1.00^*^
		R	0.90^*^		0.90^*^	0.90^*^	0.90^*^			0.90^*^			0.90^*^		0.90^*^	0.90^*^	0.90^*^
VIT_04s0008g05210	P	A													0.93^*^		
		R															
	S	A															
		R															
VIT_04s0023g00130	P	A	0.97^**^		0.94^*^	0.93^*^	0.93^*^								0.96^**^	0.94^*^	0.90^*^
		R	0.96^**^	0.89^*^	0.97^**^	0.96^**^	0.96^*^	0.92^*^		0.94^*^	0.93^*^				0.97^**^	0.95^*^	0.89^*^
	S	A	1.00^*^	0.90^*^	1.00^*^	1.00^*^	1.00^*^	0.90^*^		1.00^*^	0.90^*^	0.90^*^			1.00^*^	1.00^*^	1.00^*^
		R	1.00^*^	0.90^*^	1.00^*^	1.00^*^	1.00^*^	0.90^*^		1.00^*^	0.90^*^	0.90^*^			1.00^*^	1.00^*^	1.00^*^
VIT_04s0023g02610	P	A				0.94^*^		0.92^*^		0.97^**^	0.90^*^					0.94^*^	
		R										0.94^*^	0.97^**^				0.93^*^
	S	A	0.90^*^		0.90^*^	0.90^*^	0.90^*^	1.00^*^		0.90^*^		1.00^*^	0.90^*^		0.90^*^	0.90^*^	0.90^*^
		R											0.90^*^				
VIT_06s0004g07550	P	A	0.96^*^		0.96^**^		0.93^*^								0.99^**^		
		R	0.92^*^		0.94^*^		0.89^*^								0.98^**^		
	S	A	0.90^*^		0.90^*^	0.90^*^	0.90^*^			0.90^*^			0.90^*^		0.90^*^	0.90^*^	0.90^*^
		R	1.00^*^	0.90^*^	1.00^*^	1.00^*^	1.00^*^	0.90^*^		1.00^*^	0.90^*^	0.90^*^			1.00^*^	1.00^*^	1.00^*^
VIT_06s0009g01140	P	A	0.89^*^		0.92^*^										0.96^*^		
		R													0.92^*^		
	S	A	1.00^*^	0.90^*^	1.00^*^	1.00^*^	1.00^*^	0.90^*^		1.00^*^	0.90^*^	0.90^*^			1.00^*^	1.00^*^	1.00^*^
		R	1.00^*^	0.90^*^	1.00^*^	1.00^*^	1.00^*^	0.90^*^		1.00^*^	0.90^*^	0.90^*^			1.00^*^	1.00^*^	1.00^*^
VIT_07s0031g01930	P	A	0.99^**^		1.00^**^	0.92^*^	0.98^**^	0.89^*^			0.89^*^	0.89^*^			0.99^**^	0.92^*^	0.92^*^
		R	0.91^*^		0.91^*^	0.90^*^	0.88^*^			0.88^*^					0.93^*^	0.90^*^	
	S	A	1.00^*^	0.90^*^	1.00^*^	1.00^*^	1.00^*^	0.90^*^		1.00^*^	0.90^*^	0.90^*^			1.00^*^	1.00^*^	1.00^*^
		R	0.90^*^		0.90^*^	0.90^*^	0.90^*^			0.90^*^					0.90^*^	0.90^*^	0.90^*^
VIT_10s0003g00880	P	A	0.94^*^		0.95^*^	0.89^*^	0.92^*^								0.98^**^	0.88^*^	
		R	0.95^*^		0.97^**^	0.89^*^	0.94^*^								0.99^**^	0.88^*^	
	S	A	1.00^*^	0.90^*^	1.00^*^	1.00^*^	1.00^*^	0.90^*^		1.00^*^	0.90^*^	0.90^*^			1.00^*^	1.00^*^	1.00^*^
		R	1.00^*^	0.90^*^	1.00^*^	1.00^*^	1.00^*^	0.90^*^		1.00^*^	0.90^*^	0.90^*^			1.00^*^	1.00^*^	1.00^*^
VIT_10s0003g03190	P	A	0.89^*^												0.91^*^		
		R															
	S	A	0.90^*^		0.90^*^	0.90^*^	0.90^*^			0.90^*^			0.90^*^		0.90^*^	0.90^*^	0.90^*^
		R															
VIT_12s0028g03860	P	A				0.93^*^		0.89^*^		0.95^*^						0.93^*^	
		R	0.93^*^	0.92^*^	0.96^**^	0.91^*^	0.94^*^			0.89^*^	0.90^*^				0.96^**^	0.88^*^	
	S	A	1.00^*^	0.90^*^	1.00^*^	1.00^*^	1.00^*^	0.90^*^		1.00^*^	0.90^*^	0.90^*^			1.00^*^	1.00^*^	1.00^*^
		R	0.90^*^	1.00^*^	0.90^*^	0.90^*^	0.90^*^			0.90^*^	1.00^*^				0.90^*^	0.90^*^	0.90^*^
VIT_14s0066g01090	P	A								0.91^*^							
		R	0.99^**^		0.99^**^	0.93^*^	0.97^**^	0.88^*^				0.88^*^			1.00^**^	0.93^*^	0.92^*^
	S	A	0.90^*^		0.90^*^	0.90^*^	0.90^*^			0.90^*^					0.90^*^	0.90^*^	0.90^*^
		R	1.00^*^	0.90^*^	1.00^*^	1.00^*^	1.00^*^	0.90^*^		1.00^*^	0.90^*^	0.90^*^			1.00^*^	1.00^*^	1.00^*^
VIT_19s0014g03300	P	A	0.93^*^		0.91^*^	0.96^*^	0.91^*^	0.91^*^		0.95^*^	0.89^*^				0.91^*^	0.95^*^	
		R	0.90^*^		0.89^*^										0.94^*^		
	S	A	1.00^*^	0.90^*^	1.00^*^	1.00^*^	1.00^*^	0.90^*^		1.00^*^	0.90^*^	0.90^*^			1.00^*^	1.00^*^	1.00^*^
		R															
VIT_00s0214g00090	P	A	0.96^**^		0.98^**^		0.95^*^								0.99^**^		
		R	0.98^**^		0.99^**^	0.95^*^	0.97^**^	0.91^*^		0.89^*^	0.91^*^				0.99^**^	0.94^*^	0.90^*^
	S	A	1.00^*^	0.90^*^	1.00^*^	1.00^*^	1.00^*^	0.90^*^		1.00^*^	0.90^*^	0.90^*^			1.00^*^	1.00^*^	1.00^*^
		R	1.00^*^	0.90^*^	1.00^*^	1.00^*^	1.00^*^	0.90^*^		1.00^*^	0.90^*^	0.90^*^			1.00^*^	1.00^*^	1.00^*^

*Mean values of the biological replicates available for each set of data were calculated and all the variables were expressed as log_2_ fold changes at the stages E-L 34.5, 36, 38, and 39 relative to the stage E-L 31. For the metabolites showing a discordant profile in one season, the average of the two other seasons was considered if their profiles were correlated or consistent (§ = average 2005–2006, # = average 2006–2007). The same was done in the case of rose oxide, which was analyzed only in 2005 and 2006. Light and dark gray coloring indicate significant correlations between transcriptional and metabolic data at the 0.05 and 0.01 level, respectively. P, Pearson correlation; S, Spearman correlation; A, microarray analysis; R, qRT-PCR analysis*.

## Conclusion

Understanding the origin of grape aromatic compounds is essential in the breeding of new varieties and in the management of high-quality crops in a changing climate. In this work, previously undescribed gene-to-metabolite networks with a possible association to grape flavor were deduced by integrating the expression profiles of 4,450 gene tags and the accumulation profiles of 52 metabolites. Pairwise correlation and clustering methods pointed to several structural and regulatory genes potentially involved in the biosynthesis of monoterpenes, which paves the way for locating candidates for at least some of the missing links in the underlying pathway. Our collective findings contribute toward understanding the regulation of secondary metabolism in Muscat-type grape cultivars through the formulation of testable hypotheses regarding the function of specific genes.

## Author contributions

LC, CK, JB, FE, SD, and MG contributed to the project design; LC, MT, JB, FE, MS, and CC took part in the experimental work; RL provided the metabolic analysis; LC, CK, and MM performed the statistical and bioinformatic analyses; LC and CK were involved in data interpretation; LC wrote the manuscript. All the authors approved the final version of this text.

## Funding

This research was sustained by a Short Term Scientific Mission grant awarded to LC by the Institute of Vine and Wine Sciences (Bordeaux, France) and with the financial support provided by the Autonomous Province of Trento (*Accordo di Programma*).

### Conflict of interest statement

The authors declare that the research was conducted in the absence of any commercial or financial relationships that could be construed as a potential conflict of interest.
