# Supplementary material for: Drawing Links from Transcriptome to Metabolites: The Evolution of Aroma in the Ripening Berry of Moscato Bianco (Vitis vinifera L.)
Source: Front Plant Sci. 2017 May 16;8:780. doi: 10.3389/fpls.2017.00780 (PMC5432621; doi:10.3389/fpls.2017.00780)
Supplement: Supplementary file 1 [file Table1.docx]

**Supplementary Table S1:** Repeatability test of the method adopted for metabolic analysis (see Supplementary Method 1). Mean of six repetitions and standard deviation (SD) are shown for the most abundant and relevant metabolites from Moscato Bianco berries at technological maturity in 2006. Concentrations are expressed as µg/kg of berries.

| **Metabolite** | **Free** | | **Bound** | |
| --- | --- | --- | --- | --- |
|  | **Mean** | **% SD** | **Mean** | **% SD** |
| Linalool | 385.8 | 7.3 | 135.5 | 8.5 |
| Geraniol | 108.8 | 7.8 | 236.4 | 11.8 |
| Nerol | 42.4 | 9.3 | 264.3 | 10.7 |
| *trans*-8-hydroxy-linalool | 32.9 | 26.3 | 99.5 | 14.3 |
| *cis*-8-hydroxy-linalool | 27.1 | 45.4 | 59.9 | 8.2 |
| *trans*-furan linalool oxide | 16.4 | 6.5 | 47.8 | 8.6 |
| *cis*-furan linalool oxide | 16.2 | 6.9 | 18.7 | 6.1 |
| *trans*-pyran linalool oxide | 223.8 | 8.4 | 36.4 | 3.9 |
| *cis*-pyran linalool oxide | 45.6 | 8.3 | 3.2 | 10.1 |
| *trans*-geranic acid | 100.1 | 19.9 | 513.3 | 13.9 |
| 7-hydroxy-geraniol | 20.2 | 35.6 | 17.9 | 11.3 |
| Citronellol | 8.6 | 8.9 | 18.9 | 11.7 |
| α-terpineol | 4.4 | 12.8 | 22.4 | 13.8 |
| Hydroxy-diendiol I + hydroxy-trienol | 636.0 | 23.6 | 120.0 | 14.3 |
| Hydroxy-diendiol II | 119.5 | 16.0 | 8.4 | 21.3 |
| 3-hydroxy-β-damascone | 0.5 | 25.6 | 15.3 | 16.8 |
| 3-oxo-α-ionol | 1.0 | 24.6 | 23.8 | 14.2 |
| Benzyl alcohol | 32.7 | 5.3 | 55.0 | 7.9 |
| 2-phenylethanol | 40.6 | 6.3 | 84.3 | 3.4 |
| Hexanol | 126.2 | 5.2 | 16.2 | 7.2 |
| *trans*-3-hexen-1-ol | 1.4 | 9.2 | 0.5 | 36.4 |
| *cis*-3-hexen-1-ol | 100.2 | 2.1 | 10.4 | 9.8 |
