# Supplementary material for: Drawing Links from Transcriptome to Metabolites: The Evolution of Aroma in the Ripening Berry of Moscato Bianco (Vitis vinifera L.)
Source: Front Plant Sci. 2017 May 16;8:780. doi: 10.3389/fpls.2017.00780 (PMC5432621; doi:10.3389/fpls.2017.00780)
Supplement: Supplementary file 3 [file Table3.docx]

**Supplementary** **Table S3:** List of genes assayed for expression by qPCR. For each, the CRIBI gene name (when available) and identifier, newly designed forward/reverse primers, corresponding probe on the Grape AROS V1.0 array, coefficient of correlation between log_2_-transformed microarray and qPCR data, and relative p-value are shown.

| **CRIBI gene name/ID** | **Forward/reverse primer sequence** | **Grape AROS V1.0 Oligo ID** | **Pearson correlation coefficient** | **P-value** |
| --- | --- | --- | --- | --- |
| *NAC071*  VIT_02s0012g01040 | cggcaaattcaacttccccag/  cagcaaggacaagagactgtatc | Vv_10010907 | 0.96 | 0.0109 |
| *HDR*  VIT_03s0063g02030 | tgcgttatcaccacacaccg/  gcaagaaagccgagatgtgc | Vv_10000446 | 0.98 | 0.0026 |
| *HY5*  VIT_04s0008g05210 | atgggaggaagtttgaggcc/  taatggccttcgttcggtgc | Vv_10007020 | 0.47 | 0.4207 |
| VIT_04s0023g00130 | ttgaatgagggaggtggctc/  tacaaagccccatccagcag | Vv_10009141 | 0.96 | 0.0098 |
| VIT_04s0023g02610 | catggtcgggaacaagtgtc/  ttgcaccgtggcaaggatc | Vv_10006996 | 0.70 | 0.1893 |
| VIT_06s0004g07550 | aggagtcgagcaaaagggtg/  gaggatgatgagccggtgag | Vv_10009860 | 0.99 | 0.0009 |
| VIT_06s0009g01140 | caccactctgatcagtcccc/  cagggctgcaacaatggtg | Vv_10008655 | 0.99 | 0.0010 |
| VIT_07s0031g01930 | caaggtcgcaaagcaactgg/  gaagctgctgacacaatgcc | Vv_10009798  Vv_10010260  Vv_10014254 | 0.83  0.85  0.89 | 0.0797  0.0669  0.0411 |
| *NUDX15*  VIT_10s0003g00880 | tgatagtagttcctgtggttggc/  tcagaagcatttggagctgg | Vv_10000710 | 1.00 | 0.0003 |
| VIT_10s0003g03190 | tgcgctcagggaatcttctc/  tcggttgagtggtcttctgg | Vv_10008513 | 0.93 | 0.0201 |
| *RMA1*  VIT_12s0028g03860 | atgcacctctaacacccacc/  ggtatgtgctggtcgttgtc | Vv_10008596 | 0.86 | 0.0610 |
| *MYB24*  VIT_14s0066g01090 | acattcactgcgcagagttc/  atgaaggacccggaaaagcc | Vv_10006719 | 0.63 | 0.2516 |
| *DXR*  VIT_17s0000g08390 | tgccaaggtatgtggttcattg/  gccattaaaatgactagagactcc | Vv_10005013 | 0.86 | 0.0606 |
| *NAC2*  VIT_19s0014g03300 | attccatcgccactctgctc/  atgccagggagttgaaaggg | Vv_10003977 | 0.90 | 0.0389 |
| *PP2-B1*  VIT_00s0214g00090 | aagcaagtatcccgagtccg/  cgaaaacactgatgccaccg | Vv_10005693  Vv_10008803  Vv_10013342 | 0.97  0.80  0.77 | 0.0048  0.1038  0.1313 |
| **Reference genes** | | | | |
| VIT_06s0004g03220 | gaactgggtgcttgataggc/  aaccaaaatatccggagtaaaaga | Vv_10013333 |  |  |
| *GAPC2*  VIT_17s0000g10430 | ttctcgttgagggctattcca/  ccacagacttcatcggtgaca | Vv_10000156 |  |  |
