# Supplementary material for: Drawing Links from Transcriptome to Metabolites: The Evolution of Aroma in the Ripening Berry of Moscato Bianco (Vitis vinifera L.)
Source: Front Plant Sci. 2017 May 16;8:780. doi: 10.3389/fpls.2017.00780 (PMC5432621; doi:10.3389/fpls.2017.00780)
Supplement: Supplementary file 4 [file Table4.docx]

**Supplementary Table S4:** Differential accumulation of metabolites at stages 2-5 compared to stage 1.

| **Metabolite** | **Ratio 2vs1** | **Ratio 3vs1** | **Ratio 4vs1** | **Ratio 5vs1** |
| --- | --- | --- | --- | --- |
| *Monoterpenoids* |  |  |  |  |
| Free linalool | 6.04 | 51.72 | 71.34 | 44.79 |
| Bound linalool | 4.03 | 41.09 | 125.88 | 197.81 |
| Free geraniol | 3.89 | 5.21 | 3.84 | 6.01 |
| Bound geraniol | 1.65^NS^ | 5.54 | 7.46 | 9.67 |
| Free nerol | 7.55 | 19.90 | 13.55 | 22.88 |
| Bound nerol | 3.65 | 16.11 | 22.52 | 26.47 |
| Free *trans*-8-hydroxy-linalool | 1.29^NS^ | 9.31 | 8.41 | 6.64 |
| Bound *trans*-8-hydroxy-linalool | 0.86^NS^ | 1.58^NS^ | 2.31 | 3.89 |
| Free *cis*-8-hydroxy-linalool | 14.12 | 22.44 | 7.37 | 6.70 |
| Bound *cis*-8-hydroxy-linalool | 1.43^NS^ | 3.05 | 3.24 | 4.12 |
| Free *trans-*furan linalool oxide | 0.77^NS^ | 0.60^NS^ | 0.81^NS^ | 0.79^NS^ |
| Bound *trans-*furan linalool oxide | 1.58^NS^ | 2.31 | 3.17 | 3.62 |
| Free *cis*-furan linalool oxide | 0.33 | 0.54^NS^ | 0.64^NS^ | 0.47 |
| Bound *cis*-furan linalool oxide | 0.89^NS^ | 0.86^NS^ | 0.66^NS^ | 0.66^NS^ |
| Free *trans*-pyran linalool oxide | 0.78^NS^ | 0.92^NS^ | 1.18^NS^ | 1.12^NS^ |
| Bound *trans*-pyran linalool oxide | 1.28^NS^ | 1.31^NS^ | 1.48^NS^ | 2.15 |
| Free *cis*-pyran linalool oxide | 0.45 | 0.93^NS^ | 1.03^NS^ | 0.64^NS^ |
| Bound *cis*-pyran linalool oxide | 0.98^NS^ | 1.31^NS^ | 1.44^NS^ | 1.72^NS^ |
| Free *trans*-geranic acid | 3.46 | 6.24 | 2.99 | 3.39 |
| Bound *trans*-geranic acid | 1.09^NS^ | 1.59^NS^ | 1.49^NS^ | 1.84^NS^ |
| Free 7-hydroxy-geraniol | 2.81 | 5.89 | 3.43 | 2.30 |
| Bound 7-hydroxy-geraniol | 0.93^NS^ | 0.75^NS^ | 0.55^NS^ | 0.83^NS^ |
| Free 7-hydroxy-nerol | 1.24^NS^ | 2.37 | 1.00^NS^ | 1.75^NS^ |
| Bound 7-hydroxy-nerol | 0.24 | 0.79^NS^ | 0.56^NS^ | 1.06^NS^ |
| Free citronellol | 1.94^NS^ | 2.45 | 1.37^NS^ | 1.79^NS^ |
| Bound citronellol | 0.58^NS^ | 1.74^NS^ | 2.16 | 2.06 |
| Free 7-hydroxy-citronellol | 2.10 | 4.79 | 1.81^NS^ | 1.72^NS^ |
| Bound 7-hydroxy-citronellol | 0.84^NS^ | 0.69^NS^ | 0.41 | 0.60^NS^ |
| Free α-terpineol | 0.44 | 1.65^NS^ | 1.84^NS^ | 1.74^NS^ |
| Bound α-terpineol | 1.15^NS^ | 1.59^NS^ | 1.37^NS^ | 2.13 |
| Free 4-terpineol | 0.66^NS^ | 0.69^NS^ | 0.64^NS^ | 0.65^NS^ |
| Bound 4-terpineol | 1.12^NS^ | 1.13^NS^ | 0.77^NS^ | 1.42^NS^ |
| Free hydroxy-diendiol I | 0.35 | 0.34 | 0.28 | 0.20 |
| Bound hydroxy-diendiol I | 2.03 | 3.98 | 6.58 | 8.32 |
| Free hydroxy-diendiol II | 1.17^NS^ | 13.75 | 24.66 | 32.24 |
| Bound hydroxy-diendiol II | 1.42^NS^ | 5.43 | 9.54 | 23.09 |
| Free hydroxy-trienol | 0.24 | 0.37 | 0.20 | 0.12 |
| Bound hydroxy-trienol | 1.97^NS^ | 1.07^NS^ | 0.81^NS^ | 1.53^NS^ |
| Free rose oxide I | 0.64^NS^ | 1.43^NS^ | 1.38^NS^ | 1.31^NS^ |
| Bound rose oxide I | 1.02^NS^ | 1.44^NS^ | 2.27 | 2.30 |
| Free rose oxide II | 1.00^NS^ | 1.39^NS^ | 1.22^NS^ | 1.13^NS^ |
| Bound rose oxide II | 1.00^NS^ | 1.11^NS^ | 1.35^NS^ | 1.43^NS^ |
| *C_13_-norisoprenoids* |  |  |  |  |
| Free 3-hydroxy-β-damascone | 1.64^NS^ | 1.47^NS^ | 1.00^NS^ | 0.99^NS^ |
| Bound 3-hydroxy-β-damascone | 2.62 | 0.68^NS^ | 0.42 | 0.58^NS^ |
| Free 3-oxo-α-ionol | 2.01 | 2.20 | 0.96^NS^ | 0.53^NS^ |
| Bound 3-oxo-α-ionol | 2.80 | 3.88 | 2.82 | 3.16 |
| Free 6-methyl-5-hepten-2-one | 0.62^NS^ | 0.46 | 0.57^NS^ | 0.69^NS^ |
| Bound 6-methyl-5-hepten-2-one | 1.00^NS^ | 0.79^NS^ | 0.70^NS^ | 0.73^NS^ |
| *Phenylpropanoids/Benzenoids* |  |  |  |  |
| Free phenol | 1.19^NS^ | 1.54^NS^ | 0.92^NS^ | 0.81^NS^ |
| Bound phenol | 1.00^NS^ | 1.60^NS^ | 1.09^NS^ | 1.27^NS^ |
| Free benzyl alcohol | 1.95^NS^ | 2.10 | 1.52^NS^ | 1.90^NS^ |
| Bound benzyl alcohol | 1.45^NS^ | 1.62^NS^ | 2.01 | 1.99^NS^ |
| Free benzaldehyde | 0.65^NS^ | 0.79^NS^ | 0.59^NS^ | 0.47 |
| Bound benzaldehyde | 0.88^NS^ | 1.26^NS^ | 1.16^NS^ | 1.07^NS^ |
| Free 2-phenylethanol | 1.80^NS^ | 2.68 | 1.45^NS^ | 2.14 |
| Bound 2-phenylethanol | 1.24^NS^ | 1.37^NS^ | 1.48^NS^ | 1.60^NS^ |
| Free methyl salicylate | 0.82^NS^ | 0.73^NS^ | 0.62^NS^ | 0.57^NS^ |
| Bound methyl salicylate | 3.65 | 2.65 | 4.06 | 2.70 |
| *C_6_ aliphatic compounds* |  |  |  |  |
| Free hexanol | 1.54^NS^ | 1.99^NS^ | 2.23 | 1.83^NS^ |
| Bound hexanol | 3.05 | 4.86 | 6.74 | 6.80 |
| Free *trans*-3-hexen-1-ol | 1.34^NS^ | 2.03 | 1.45^NS^ | 2.00 |
| Bound *trans*-3-hexen-1-ol | 1.13^NS^ | 1.38^NS^ | 1.28^NS^ | 1.38^NS^ |
| Free *cis*-3-hexen-1-ol | 1.47^NS^ | 1.26^NS^ | 0.84^NS^ | 0.55^NS^ |
| Bound *cis*-3-hexen-1-ol | 3.65 | 4.81 | 3.81 | 2.05 |

NS = not significant (the significance is achieved with a 2-fold change between time points).
