# Supplementary material for: Drawing Links from Transcriptome to Metabolites: The Evolution of Aroma in the Ripening Berry of Moscato Bianco (Vitis vinifera L.)
Source: Front Plant Sci. 2017 May 16;8:780. doi: 10.3389/fpls.2017.00780 (PMC5432621; doi:10.3389/fpls.2017.00780)
Supplement: Supplementary file 9 [file Table9.docx]

**Supplementary Table S9:** Genes with a potential involvement in the accumulation of monoterpenes during Moscato Bianco berry ripening. Probes marked with $ were not differentially expressed in the pairwise comparison between time points (with a cut-off of 2-fold change and a false discovery rate < 1%). Functional annotation was derived from Additional file 1 by Grimplet *et al.* (2012). Abbreviation: BC = bicluster.

| **Probe ID** | **V1 gene prediction** | **Functional annotation** | **Evidences from the present work** | **Evidences from the literature** |
| --- | --- | --- | --- | --- |
| **Terpene biosynthesis** | | | | |
| Vv_10000446$ | VIT_03s0063g02030 | 4-hydroxy-3-methylbut-2-enyl diphosphate reductase | Profile: 2vs1,4vs1,5vs1up (qRT-PCR_2016)  Cluster 5 (like free OxA) | HDR plays a major role in controlling the production of MEP-derived precursors for plastid isoprenoid biosynthesis in tomato and *Arabidopsis thaliana* (Botella-Pavía *et al.*, 2004)  The expression of *VvHDR* parallels monoterpene accumulation in Gewürztraminer and Muscat Blanc à petits grains (Martin *et al.*, 2012; Wen *et al.*, 2015) |
| Vv_10000710$ | VIT_10s0003g00880 | Nudix hydrolase 15 | Profile: 4vs1up (qRT-PCR_2016)  Positive correlation with bound *trans*-8-HO-linalool and OxC  Cluster 4 | RhNUDX1 is part of a newly discovered pathway for the biosynthesis of free monoterpene alcohols. It shows geranyl diphosphate diphosphohydrolase activity *in vitro* and supports geraniol biosynthesis *in planta* (Magnard *et al.*, 2015)  VIT_10s0003g00880 is predicted to be localized also in the chloroplast |
| Vv_10004183 | VIT_18s0001g04280 VIT_18s0001g04530  and others | (-)-germacrene D synthase | Profile: 5vs1up | VIT_18s0001g04280 and VIT_18s0001g04530 correlate with linalool and α-terpineol in Tocai Friulano under water deficit (Savoi *et al.*, 2016) |
| **Monoterpene metabolism** | | | | |
| Vv_10009587 | VIT_01s0137g00410 | CYP86A2 | Profile: 3vs1,4vs1down  Positive correlation with free 6-methyl-5-hepten-2-one  Cluster 5 (like free OxA and 6-methyl-5-hepten-2-one)  BCs 209 (like free 6-methyl-5-hepten-2-one), 353, 370 (like free OxA, 6-methyl-5-hepten-2-one, bound OxC, HO-diendiol I + HO-trienol) | In apple 6-methyl-5-hepten-2-one is an oxidation product of α-farnesene (Whitaker and Saftner, 2000)  VIT_01s0137g00410 is predicted to be localized in the chloroplast |
| Vv_10009014 | VIT_00s0389g00040 (70/70)  VIT_00s0389g00030 (67/70)  (chr2) | CYP72A1 | Profile: 5vs1down  Clusters 8+3+7 (like free OxD, HO-diendiol I + HO-trienol, rose oxide II)  BCs 219, 274, 302 (like free HO-diendiol I + HO-trienol, bound OxC) | VIT_00s0389g00030 and VIT_00s0389g00040 fall into the confidence interval of a QTL for linalool, linalool/nerol and nerol/geraniol (Doligez *et al.*, 2006; Battilana *et al.*, 2009)  In VTCdb VIT_00s0389g00030 is coexpressed with genes enriched in the category ‘Chloroplast part’, like *VvDXS1* (VIT_05s0020g02130) and the linalool/nerolidol synthase *VvRiLinNer*/*VvPNLinNer2*/*VvCSLinNer*  VIT_00s0389g00030 is significantly more expressed in the skin than in the pulp of Cabernet Sauvignon berries at 23 °Brix (Cramer *et al.*, 2014); it is predicted to be localized in the chloroplast |
| Vv_10001518  Vv_10001519$ | VIT_15s0048g01490 | Geraniol 10-hydroxylase (CYP76) | Profile: 3vs1,4vs1up  Cluster 8 (like free OxD and rose oxide II)  BCs 197 (like bound OxC, HO-diendiol I + HO-trienol), 253, 267, 270 (like free HO-diendiol I+ HO-trienol, bound OxC, HO-diendiol I + HO-trienol), 323 (like free HO-diendiol I + HO-trienol) | Members of the CYP76 family metabolize linalool in *Arabidopsis thaliana* (Ginglinger *et al.*, 2013; Höfer *et al.*, 2014; Boachon *et al.*, 2015) and grapevine (Ilc *et al.*, 2017)  VIT_15s0048g01490 is significantly more expressed in the skin than in the pulp of Cabernet Sauvignon berries at 23 °Brix (Cramer *et al.*, 2014)  The expression of *CYP76T21* increases along Gewürztraminer berry ripening. CYP76T21 is able to convert linalool to *trans*-8-HO-linalool, to metabolize *trans*-8-HO-linalool and to oxidize *trans*-8-oxo-linalool to *trans*-8-carboxy-linalool *in vitro*. It is also able to metabolize linalool and to produce *trans*-8-carboxy-linalool *in planta* (Ilc *et al.*, 2017)  VIT_15s0048g01490 is predicted to be localized in the chloroplast |
| Vv_10002015 | VIT_15s0048g01590 | CYP76B1 | Profile: 3vs1down  Cluster 7  BCs 340 and 403, like free OxA | Members of the CYP76 family metabolize linalool in *Arabidopsis thaliana* (Ginglinger *et al.*, 2013; Höfer *et al.*, 2014; Boachon *et al.*, 2015) and grapevine (Ilc *et al.*, 2017)  In VTCdb VIT_15s0048g01590 is coexpressed with the linalool/nerolidol/geranyl linalool synthases *VvPNLNGl1*, *VvPNLNGl2*, *VvPNLNGl3* *VvPNLNGl4*, and the 1,8-cineole synthase VIT_00s0572g00020  VIT_15s0048g01490 is predicted to be localized in the chloroplast |
| Vv_10006928$ | VIT_15s0048g01700 | CYP86A1 | Cluster 9 (like free linalool, OxB, OxC)  BC 345 (like bound OxB) | In VTCdb VIT_15s0048g01700 is coexpressed with the linalool/nerolidol/geranyl linalool synthase *VvPNLNGl3*  VIT_15s0048g01700 is significantly more expressed in the skin than in the pulp of Cabernet Sauvignon berries at 23 °Brix (Cramer *et al.*, 2014); it is predicted to be localized in the chloroplast |
| Vv_10006984$ | VIT_16s0039g00880 | CYP89H3 | Positive correlation with free linalool, OxB, α terpineol, bound citronellol  Cluster 9 (like free linalool, OxB, OxC  BCs 4 (like bound OxB), 35 (like bound OxB, OxC), 98 (like bound OxB) | VIT_16s0039g00880 is up-regulated at E-L 36 vs E-L 35 in three aromatic varieties (Agudelo-Romero *et al.*, 2013)  VIT_16s0039g00880 is predicted to be localized also in the chloroplast |
| Vv_10007610 | VIT_18s0001g06230 | No hit (NADPH-cytochrome P450 reductase) | Profile: 2vs1,3vs1,4vs1,5vs1down  Positive correlation with free linalool, *trans*-8-HO-linalool, α-terpineol, HO-diendiol II, rose oxide I, bound nerol, citronellol  Cluster 9, like free linalool, OxB, OxC  BCs 4 (like bound OxB), 15 (like free *cis*-8-HO-linalool, HO-diendiol I + HO-trienol, *trans*-geranic acid, bound OxB), 35 (like bound OxB, OxC) | - |
| Vv_10004784 | VIT_18s0001g13790 | Cytochrome P450, family 83, subfamily B, polypeptide 1 (CYP71) | Profile: 3vs1up,4vs1up,5vs1up  Positive correlation with several oxidized monoterpenes (bound *cis*/*trans* 8-HO-linalool, OxA, OxD, *trans*-geranic acid, HO-diendiol I and II)  Cluster 4  BCs 10, 39 (like free *cis*-8-HO-linalool, *trans*-geranic acid, HO-diendiol I + HO-trienol), 20 (like free *cis*-8-HO-linalool, OxB, *trans*-geranic acid, HO-diendiol I + HO-trienol) | Members of the CYP71 family metabolize linalool in *Arabidopsis thaliana* (Ginglinger *et al.*, 2013)  The expression of *CYP71AT7* increases along Gewürztraminer berry ripening. CYP71AT7 is able to consume low amounts of linalool and *trans*-8-HO-linalool *in vitro* (Ilc *et al.*, 2017)  VIT_18s0001g13790 is predicted to be localized in the chloroplast |
| Vv_10006996 | VIT_04s0023g02610 | Epoxide hydrolase 2 | Profile: 5vs1up (array) | VIT_04s0023g02610 falls within the confidence interval of a QTL for geraniol and nerol (Battilana *et al.*, unpublished data) and is predicted to be localized also in the chloroplast. It correlates with nerol in Tocai Friulano under water deficit (Savoi *et al.*, 2016) |
| Vv_10009847$ | VIT_07s0005g00700 | Epoxide hydrolase | Positive correlation with free linalool, α-terpineol, HO-diendiol II, rose oxide I, bound linalool, geraniol, nerol, *cis*-8-hydroxy-linalool, OxD, citronellol  Clusters 4 (like free HO-diendiol II, bound *cis*/*trans*-8-HO-linalool, OxA, OxC, OxD, HO-diendiol I + HO-trienol, HO-diendiol II, rose oxide I and II) +9 (like free OxB, OxC) +6 (like bound *trans*-geranic acid)  BC 15 (like free *cis*-8-HO-linalool, HO-diendiol I + HO-trienol, *trans*-geranic acid, bound OxB) | VIT_07s0005g00700 is predicted to be localized also in the chloroplast |
| Vv_10003763 | VIT_06s0061g01350 | Cyclase | Profile: 4vs1up  Cluster 8 (like free rose oxide II)  BCs 219 (like bound 4-terpineol), 383, 392 | Rose oxide and 4-terpineol are cyclic monoterpenoids |
| Vv_10010475$  Vv_10004634  Vv_10013426 | VIT_03s0091g00040  (*VvgGT1*-*VvGT11*)  VIT_03s0180g00200  (*VvgGT2*-*VvGT9*)  VIT_03s0180g00320  (*VvgGT3*-*VvGT10*) | Limonoid UDP-glucosyltransferase  Limonoid UDP-glucosyltransferase  Indole-3-acetate beta-glucosyltransferase | Cluster 2 (like bound OxB)  BCs 273, 277, 292, 349, like bound OxB, rose oxide I and II, 3-oxo-α-ionol, benzyl alcohol  Profile: 3vs1down  Cluster 5  BCs 230, 253, 267, 392, like bound OxC, HO-diendiol I + HO-trienol, *cis*-3-hexen-1-ol  Profile: 3vs1down  Cluster 5  BCs 197, 230, 253, 267, 270, like bound OxC, HO-diendiol I + HO-trienol, 4-terpineol, *cis*-3-hexen-1-ol | CitLGT regulates the conversion of limonoid aglycones (triterpenes) to glucosides in citrus fruit (Kita *et al.*, 2000)  The expression levels of VIT_03s0091g00040, VIT_03s0180g00200 and VIT_03s0180g00320 gradually decrease during berry development in 5 different varieties and clones; only in Muscat the expression of VIT_03s0180g00200 rises at late ripening stages (Bönisch *et al.*, 2014a)  In Sémillon VIT_03s0180g00320 is up-regulated by noble rot, along with an increase in terpene biosynthesis (Blanco-Ulate *et al.*, 2015)  VIT_03s0091g00040, VIT_03s0180g00200 and VIT_03s0180g00320 are predicted to be localized in the chloroplast |
| Vv_10010567$ | VIT_03s0180g00280 (*VvGT18*) | Indole-3-acetate β-glucosyltransferase | Cluster 1  BCs 0 (like bound OxB), 13 (like bound OxB), 19 (like bound OxB), 199 (like bound OxB, OxC, α-terpineol) | AtGT84B1 and AtGT84B2 show *in vitro* activity towards model linalool (Caputi *et al.*, 2008)  CsUGT1 is a putative terpenoid UGT from sweet orange (Fan *et al.*, 2010)  The expression of VIT_03s0180g00280 decreases after *veraison* in 5 different varieties and clones (Bönisch *et al.*, 2014b) |
| Vv_10006855$  Vv_10008432$ | VIT_05s0062g00430 (*VvGT20*)  VIT_05s0062g00520 | UDP-glucose:flavonoid 7-O-glucosyltransferase | Cluster 1 (like bound 3-hydroxy-β-damascone, 6-methyl-5-hepten-2-one)  BCs 0 and 119, like bound OxB, 3-hydroxy-β-damascone, 6-methyl-5-hepten-2-one  Clusters 7+1  BCs 317, 355, 377, like bound 3-hydroxy-β-damascone, 6-methyl-5-hepten-2-one, *cis*/*trans*-3-hexen-1-ol | VIT_05s0062g00430 matches to two monoterpene glucosyltransferases from Eucalyptus (Nagashima *et al.*, 2005)  The expression of VIT_05s0062g00430 decreases after *veraison* in 5 different varieties and clones (Bönisch *et al.*, 2014b)  VIT_05s0062g00430 and VIT_05s0062g00520 are predicted to be localized in the chloroplast |
| Vv_10010104$ | VIT_05s0062g00630  (*VvGT8*)  VIT_05s0062g00640 | UDP-glucose transferase (UGT75B2)  UDP-glucose:flavonoid 7-O-glucosyltransferase | Cluster 3 (like bound *cis*-3-hexen-1-ol)  BCs 82, 90, 92, 107, 116, 124, 141, 152, like bound *trans*-8-HO-linalool, OxB, OxC, α-terpineol, HO-diendiol I + HO-trienol, *cis*-3-hexen-1-ol | VIT_05s0062g00640 matches to two monoterpene glucosyltransferases from *Eucalyptus* (Nagashima *et al.*, 2005) and a linalool glucosyltransferase from *Arabidopsis* (Caputi *et al.*, 2008)  The expression level of VIT_05s0062g00630 gradually decreases during berry development in 5 different varieties and clones (Bönisch *et al.*, 2014a)  VIT_05s0062g00640 is predicted to be localized in the chloroplast |
| Vv_10013486$ | VIT_07s0141g00580 | Glycosyl transferase family 8 protein | Positive correlation with free linalool, *trans*-8-HO-linalool, OxB, α-terpineol, rose oxide I, bound citronellol  Cluster 9  BCs 35, 95, like bound OxB, OxC, HO-diendiol I + HO-trienol, 3-hydroxy-β-damascone, 6-methyl-5-hepten-2-one | In VTCdb VIT_07s0141g00580 is coexpressed with *GPPS* (VIT_06s0009g03090, VIT_00s0283g00050) and a CYP76C (VIT_15s0048g01480) |
| Vv_10009861 | VIT_16s0050g01580 (*VvGT7*) | UDP-glucose: anthocyanidin 5,3-O-glucosyltransferase | Profile: 3vs1,4vs1,5vs1down  Cluster 5  BCs 204 (like bound HO-diendiol I + HO-trienol), 209, 283 (like bound HO-diendiol I + HO-trienol) | VIT_16s0050g01580 matches to a *Arabidopsis* citronellol/geraniol glucosyltransferase (Caputi *et al.*, 2008)  VvGT7 may contribute to the production of geranyl and neryl glucoside during grape ripening; it glucosylates a variety of substrates (Bönisch *et al.*, 2014a)  *VvGT7* transcript accumulation correlates significantly with the accumulation of glycosylated monoterpenes in Riesling ripening grapes (Friedel *et al.*, 2016)  VIT_16s0050g01580 falls within the confidence interval of a QTL for geraniol and nerol (Doligez *et al.*, 2006) and is predicted to be localized in the chloroplast |
| Vv_10009347 | VIT_14s0006g00430 | Carboxylesterase | Profile: 3vs1,4vs1,5vs1up  Positive correlation with free OxC, bound rose oxide I, benzylic alcohol  Cluster 9 | Carboxylesterases are involved in the hydrolysis of esters into acids and alcohols and may contribute to flavor development; VIT_14s0006g00430 is up-regulated at E-L 36 vs E-L 35 in three aromatic varieties (Agudelo-Romero *et al.*, 2013) |
| **Monoterpene transport** | | | | |
| Vv_10003051  Vv_10012513  Vv_10001796$ | VIT_02s0012g01630  VIT_00s0287g00080  (chr 2) | Transmembrane protein 41B (SNARE associated Golgi protein)  Vesicle-associated membrane protein | Profile: 2vs1,3vs1,4vs1,5vs1down Cluster 9  Positive correlation with free nerol, bound *trans*-geranic acid, 7-HO-nerol and α-terpineol  Cluster 6 | Inhibition of vesicle fusion was shown to strongly interact with ectopic expression of certain terpenes, including linalool (Ting *et al.*, 2015)  VIT_02s0012g01630 and VIT_00s0287g00080 fall within the confidence interval of a QTL for linalool, linalool/nerol and nerol/geraniol (Doligez *et al.*, 2006; Battilana *et al.*, 2009)  VIT_00s0287g00080 is significantly more expressed in the skin than in the pulp of Cabernet Sauvignon berries at 23 °Brix (Cramer *et al.*, 2014); it is predicted to be localized in the chloroplast |
| Vv_10002561$  Vv_10011974 | VIT_01s0011g04670  VIT_16s0039g00010 | ABC transporter G member 2  ABC transporter G member 7 | Positive correlation with free OxB  Cluster 9  Profile: 5vs1up  Cluster 5 | Plant ABCG transporters have been shown to transport terpenoids (Kang *et al.*, 2011)  In VTCdb VIT_16s0039g00010 is coexpressed with nine monoterpene synthases (including the linalool/nerolidol synthases *VvPNLinNer1* and *VvPNLNGl3*), which determines a significant enrichment in the category ‘S-linalool synthase activity’ for the coexpressed genes  VIT_01s0011g04670 and VIT_16s0039g00010 are significantly more expressed in the skin than in the pulp of Cabernet Sauvignon berries at 23 °Brix (Cramer *et al.*, 2014) |
| Vv_10004737 | VIT_08s0040g03040 | Glutathione S-transferase GSTO1 | Profile: 5vs1up  Positive correlation with bound *trans*-8-HO-linalool, OxD, *trans*-geranic acid, α-terpineol, HO-diendiol II  Cluster 4 | In VTCdb VIT_08s0040g03040 is coexpressed with ABCG/PDR12 transporters (VIT_09s0002g05370, VIT_09s0002g05410, VIT_09s0002g05600), which causes a significant enrichment in the category ‘Isoprenoid transport’  VIT_08s0040g03040 is predicted to be localized also in the chloroplast |
| Vv_10008655 | VIT_06s0009g01140 | Amino acid permease | Profile: 2vs1,3vs1,4vs1,5vs1up (array and qRT-PCR_2016)  Positive correlation with free linalool, *trans*-8-HO-linalool, α-terpineol, HO-diendiol II, rose oxide I, bound linalool, geraniol, nerol, *cis*-8-HO-linalool, citronellol  Clusters 4+6+9 | VIT_06s0009g01140 falls within the confidence interval of a QTL for nerol/geraniol (Battilana *et al.*, 2009) and is a putative ‘switch gene’ in the immature-to-mature transition during grapevine development (Palumbo *et al.*, 2014)  In VTCdb VIT_06s0009g01140 is coexpressed with linalool synthase (VIT_00s0372g00020), 1,8-cineole synthase (VIT_00s0266g00020, VIT_00s0271g00010), and CYP76C4s (VIT_02s0012g02820, VIT_03s0097g00460) |
| Vv_10002691 | VIT_08s0007g05210 | Amino acid permease | Profile: 5vs1up  Positive correlation with free geraniol and bound α-terpineol  Cluster 6 | VIT_08s0007g05210 falls within the confidence interval of a QTL for geraniol and nerol (Battilana *et al.*, unpublished data)  In VTCdb VIT_08s0007g05210 is coexpressed with CYP76Cs (VIT_02s0025g04880, VIT_15s0048g01480 and VIT_15s0048g01490) |
| Vv_10000443  Vv_10011370 | VIT_08s0007g04900 | Unknown protein | Profile: 5vs1up  Positive correlation with several monoterpenes  Cluster 4 | VIT_08s0007g04900 is annotated with the GO term ‘Cytoplasmic membrane-bounded vesicle’; it falls within the confidence interval of a QTL for geraniol and nerol (Battilana *et al.* 2009, unpublished data) and is predicted to be localized in the chloroplast |
| **Transcriptional regulation and signaling** | | | | |
| Vv_10002171 | VIT_16s0100g00400 | Ethylene-responsive transcription factor ERF025 | Profile: 2vs1,3vs1,5vs1down  Clusters 9+7  Correlated with Vv_10003051 (correlation > 0.99 between differential gene expression ratios)  Correlated with con Vv_10003051, Vv_10006855, Vv_10007610, Vv_10009014, Vv_10009587, Vv_10012513 (correlation > 0.90 between microarray channel intensities) | VIT_16s0100g00400 is a candidate master regulator of grape berry maturation (Palumbo *et al.*, 2014)  In VTCdb VIT_16s0100g00400 is coexpressed with CYP76C4s (VIT_02s0012g02810, (VIT_03s0097g00460) and the 9-*cis*-epoxycarotenoid dioxygenase *VvCCD4b* (VIT_02s0087g00930) |
| Vv_10008748 | VIT_18s0001g05250 | DREB sub A-6 of ERF/AP2 transcription factor (RAP2.4) | Profile: 2vs1,3vs1,4vs1,5vs1down  Cluster 9  Correlated with Vv_10002015, Vv_10003051, Vv_10006855, Vv_10007610, Vv_10009014, Vv_10009587, Vv_10012513 (correlation > 0.90 between microarray channel intensities) | In Wen *et al.* (2015) XM_002285766.2 is coexpressed with the linalool/nerolidol synthase *VvCSLinNer* |
| Vv_10006673$ | VIT_10s0003g04100 | Auxin response factor 3 | Positive correlation with free OxA  Cluster 5 | *AtARF6* promotes volatile sesquiterpene production by activating jasmonate biosynthesis and *AtMYB21* expression (Reeves *et al.*, 2012)  In VTCdb VIT_10s0003g04100 is coexpressed with the linalool/nerolidol synthase *VvPNLinNer2*, a myrcene synthase (VIT_00s0271g00030), a putative monoterpene glycosyltransferase (VIT_08s0007g04590), and three CYPs involved in monoterpenoid biosynthesis or coexpressed with linalool synthase genes (VIT_10s0092g00500, VIT_00s0389g00030, VIT_00s0389g00040)  VIT_10s0003g04100 is significantly more expressed in the skin than in the pulp of Cabernet Sauvignon berries at 23 °Brix (Cramer *et al.*, 2014) |
| Vv_10006719  Vv_10007868$ | VIT_14s0066g01090 | Myb domain protein 24 | Profile: 5vs1up (array)  Positive correlation with bound OxC  Cluster 4  Vv_10007868 correlated with Vv_10004183 (correlation > 0.99 between differential gene expression ratios)  Vv_10006719 correlated with Vv_10004183 (correlation > 0.95 between microarray channel intensities) | VIT_14s0066g01090 falls within the confidence interval of a QTL for geraniol and nerol (Battilana *et al.* 2009, unpublished data); it is significantly induced by UV radiation in the berry skin of Tempranillo (Carbonell-Bejerano *et al.*, 2014b); candidate transcriptional regulator of monoterpene biosynthesis (Savoi *et al.*, 2016); it is highly co-expressed with flower and fruit specific *TPS* genes (Wong *et al.*, 2016) |
| Vv_10006438$  Vv_10003977  Vv_10007346$ | VIT_01s0146g00280  VIT_19s0014g03300 | NAC domain-containing protein 83  NAC domain containing protein 2 | Positive correlation with free nerol, bound *trans*-geranic acid, 7-OH-nerol, α-terpineol  Cluster 6  Correlated with Vv_10001796 (correlation > 0.99 between differential gene expression ratios)  Profile: 5vs1up (array)  Positive correlation with bound *trans*-8-HO-linalool, OxC, rose oxide II  Cluster 4  Correlated with Vv_10006996 and Vv_10000710 (correlation > 0.99 between differential gene expression ratios)  Positive correlation with bound rose oxide II  Cluster 4 | Tomato mutant fruit at the *NOR* locus fails to produce climacteric ethylene or ripen (Giovannoni, 2004)  NAC TFs (AaNAC2, AaNAC3 and AaNAC4) are involved in controlling monoterpene production in kiwifruit through transcriptional activation of *TPS1* (Nieuwenhuizen *et al.*, 2015)  VIT_01s0146g00280 and VIT_19s0014g03300 are up-regulated in both flesh and skin throughout Muscat Hamburg ripening (Lijavetzky *et al.*, 2012)  In Sémillon VIT_19s0014g03300 is up-regulated by noble rot, along with an increase in terpene biosynthesis (Blanco-Ulate *et al.*, 2015) |
| Vv_10008821 | VIT_15s0046g01440 | BZip transcription factor G- box binding factor 3 | Profile: 3vs1,4vs1,5vs1down  Negative correlation with several monoterpenes  Cluster 1 | CrGBF1 acts as a transcriptional repressor of the *Str* promoter (Sibéril *et al.*, 2001)  VIT_15s0046g01440 is significantly more expressed in the skin than in the pulp of Cabernet Sauvignon berries at 23 °Brix (Cramer *et al.*, 2014) |
| Vv_10004421 | VIT_18s0001g09230 | Salt tolerance zinc finger | Profile: 5vs1down  Negative correlation with bound 4-terpineol  Cluster 7 | ZCT proteins act as repressors in the regulation of elicitor-induced secondary metabolism in *Catharanthus roseus*. In particular, they repress the *Str* and *Tdc* gene expression (Pauw *et al.*, 2004)  VIT_18s0001g09230 is significantly more expressed in the skin than in the pulp of Cabernet Sauvignon berries at 23 °Brix (Cramer *et al.*, 2014) |
| Vv_10007020 | VIT_04s0008g05210 | BZIP protein HY5 (HY5) | Profile: 4vs1,5vs1up (array)  Cluster 9  Correlated with Vv_10008655 (correlation > 0.90 between microarray channel intensities) | *HY5* is a key gene involved in light perception transduction  LeHY5 is a positive regulator of thylakoid organization and carotenoid accumulation (Liu *et al.*, 2004; Toledo-Ortiz *et al.*, 2014)  AaHY5 interacts with the promoter of a pinene synthase gene in modulating its rhythmic expression (Zhou *et al.*, 2015)  *VvHY5*/*HYH* are coexpressed with genes involved in the biosynthesis of monoterpenes, including four *TPS* (Loyola *et al.*, 2016)  The expression of VIT_04s0008g05210 is induced by UV radiation (Carbonell-Bejerano *et al.*, 2014b; Liu et al., 2015; Loyola *et al.,* 2016) and is higher in Verdejo (aromatic) than in Tempranillo samples (Carbonell-Bejerano *et al.*, 2014a)  VIT_04s0008g05210 is significantly more expressed in the skin than in the pulp of Cabernet Sauvignon berries at 23 °Brix (Cramer *et al.*, 2014) |
| Vv_10002904$  Vv_10001238$ | VIT_06s0061g01140  VIT_10s0003g04490 | Phototropic-responsive NPH3  Phototropic-responsive NPH3 | Cluster 9  Correlated with Vv_10013486 (correlation > 0.99 between differential gene expression ratios)  Positive correlation with free linalool, *trans*-8-HO-linalool, α-terpineol, rose oxide I  Clusters 6+8+9+4  Correlated with Vv_10002691 and Vv_10000710 (correlation > 0.90 between microarray channel intensities) | In VTCdb VIT_10s0003g04490 is coexpressed with genes that determine a significant enrichment in the category ‘Chloroplast part’; it is significantly more expressed in the skin than in the pulp of Cabernet Sauvignon berries at 23 °Brix (Cramer *et al.*, 2014) |
| Vv_10000275$  Vv_10005663$  Vv_10006303$  Vv_10010958 | VIT_15s0048g02410 | Myb CCA1 (circadian clock associated 1) | Cluster 6  Profile: 4vs1down  Clusters 6+5+4 | *Myb CCA1* encodes a protein of the circadian oscillator, which regulates the isoprenoid pathway (Vranová *et al.*, 2012)  In VTCdb VIT_15s0048g02410 is coexpressed with genes that determine a significant enrichment in the category ‘Plastid’ |
| **New candidate genes** | | | | |
| Vv_10000182$ | VIT_01s0010g03900 | SEPALLATA3 | Positive correlation with free OxC  Cluster 9  Correlated with Vv_10000443 and Vv_10004737 (correlation > 0.90 between microarray channel intensities) | *VvMADS4* may have an important role in fruit development besides floral organ formation (Boss *et al.*, 2002) |
| Vv_10011420  Vv_10000707 | VIT_01s0026g01970 | RNA-binding region RNP-1 (RNA recognition motif) | Profile: 2vs1,3vs1,4vs1,5vs1down  Cluster 4  Correlated with Vv_10003051, Vv_10006855, Vv_10007610, Vv_10009587, Vv_10012513 (correlation > 0.90 between microarray channel intensities)  Profile: 2vs1,3vs1,4vs1,5vs1down  Clusters 5+4 | Chloroplast RNA-recognition motif proteins are candidates for regulating chloroplast RNA processing under shifting environmental conditions (Ruwe *et al.*, 2011)  VIT_01s0026g01970 falls within the confidence interval of a QTL for nerol/geraniol (Battilana *et al.*, 2009) and is predicted to be localized also in the chloroplast |
| Vv_10010907 Vv_10013994$ | VIT_02s0012g01040 | NAC domain-containing protein 71 | Profile: 2vs1,3vs1,4vs1,5vs1up (array and qRT-PCR_2016)  Positive correlation with free OxC, bound linalool, *trans*-8-HO linalool, OxA, HO-diendiol I and II, rose oxide I and II  Cluster 4  Vv_10010907 correlated with Vv_10008655 and Vv_10009347 (correlation > 0.90 between microarray channel intensities) | VIT_02s0012g01040 falls within the confidence interval of a QTL for linalool, linalool/nerol and nerol/geraniol (Doligez *et al.*, 2006; Battilana *et al.*, 2009). It is up-regulated in both flesh and skin throughout Muscat Hamburg ripening (Lijavetzky *et al.*, 2012) and is a candidate master regulator of grape berry maturation (Palumbo *et al.*, 2014) |
| Vv_10002682$ Vv_10009852$ Vv_10012211  Vv_10013897$ | VIT_02s0012g01240 (2 gene predictions in V2: Vv_10012211 matches to VIT_202s0012g01235 = ring fyve phd zinc finger-containing protein) | PHD finger transcription factor | Profile: 5vs1up  Positive correlation with free geraniol, bound α-terpineol  Cluster 6  Vv_10012211 correlated with Vv_10002691 (correlation > 0.99 between differential gene expression ratios) and Vv_10008655 (correlation > 0.90 between microarray channel intensities) | VIT_02s0012g01240 falls within the confidence interval of a QTL for linalool, linalool/nerol and nerol/geraniol (Doligez *et al.*, 2006; Battilana *et al.*, 2009) |
| Vv_10006791$ | VIT_02s0087g00770 | ABC transporter E member 1 | Cluster 9 | ABCE is a suppressor of RNA silencing (Kärblane *et al.*, 2015)  VIT_02s0087g00770 falls within the confidence interval of a QTL for linalool/nerol and nerol/geraniol, which is the second QTL on chromosome 2 (Battilana *et al.*, 2009) |
| Vv_10014336$ | VIT_02s0087g00830 | CBF1 interacting corepressor CIR | Positive correlation with bound *trans*-8-HO-linalool, OxA, OxD, HO-diendiol I and II, rose oxide II  Cluster 4  Correlated with Vv_10006996 (correlation > 0.99 between differential gene expression ratios) | VIT_02s0087g00830 falls within the confidence interval of a QTL for linalool/nerol and nerol/geraniol, which is the second QTL on chromosome 2 (Battilana *et al.*, 2009) |
| Vv_10003472 | VIT_02s0234g00100 | Ubiquitinyl hydrolase 1 | Profile: 5vs1up  Positive correlation with several bound monoterpenes  Cluster 4  Correlated with Vv_10000443 and Vv_10011370 (correlation > 0.99 between differential gene expression ratios)  Correlated with Vv_10000443, Vv_10000710 and Vv_10002691 (correlation > 0.90 between microarray channel intensities) | VIT_02s0234g00100 is predicted to be localized also in the chloroplast |
| Vv_10013322$ | VIT_00s0341g00050 (chr2) | ATMYB66/WER/WER1 (WEREWOLF 1) | Clusters 6+5+4 | VIT_00s0341g00050 falls within the confidence interval of a QTL for linalool, linalool/nerol and nerol/geraniol (Doligez *et al.*, 2006; Battilana *et al.*, 2009) and is predicted to be localized also in the chloroplast |
| Vv_10000578 | VIT_03s0038g02500 | SKP1 | Profile: 5vs1up  Correlated with Vv_10000710 and Vv_10006996 (correlation > 0.99 between differential gene expression ratios) | AtASK1 is part of the SCF complex that regulates the expression of jasmonate (JA) responsive genes (Devoto *et al.*, 2002)  JA treatment of grapes induces the expression of genes from the MVA and MEP pathways and the formation of volatile compounds, especially terpenes and norisoprenoids (D’Onofrio *et al.*, 2009; Gómez-Plaza *et al.*, 2012; May and Wüst, 2015)  VIT_03s0038g02500 is predicted to be localized also in the chloroplast |
| Vv_10001186$ Vv_10006289$ | VIT_04s0008g07340 | Constans-like 4 | Vv_10006289 positively correlated with bound *trans*-8-HO-linalool, OxD, HO-diendiol II  Cluster 4  Vv_10006289 correlated with Vv_10000443 and Vv_10000710 (correlation > 0.90 and 0.95 between microarray channel intensities, respectively) | At5g24930 correlates with isoprenoid biosynthetic genes (Mannen *et al.*, 2014)  VIT_04s0008g07340 is annotated with the GO terms ‘Jasmonic acid mediated signaling pathway’ and ‘Red light signaling pathway’ among others; it is significantly more expressed in the skin than in the pulp of Cabernet Sauvignon berries at 23 °Brix (Cramer *et al.*, 2014) and is predicted to be localized in the chloroplast |
| Vv_10009141 | VIT_04s0023g00130 | Unknown protein | Profile: 4vs1,5vs1up (array), 3vs1,4vs1,5vs1up (qRT-PCR_2016)  Positive correlation with free OxC, bound OxA, HO-diendiol I, rose oxide I and II  Cluster 4 | VIT_04s0023g00130 falls within the confidence interval of a QTL for geraniol and nerol (Battilana *et al.* 2009, unpublished data) and is predicted to localize also in the chloroplast. It is up-regulated in both flesh and skin throughout Muscat Hamburg ripening (Lijavetzky *et al.*, 2012) and is a putative ‘switch gene’ in the immature-to-mature transition during grapevine development (Palumbo *et al.*, 2014) |
| Vv_10011003$ | VIT_04s0023g00440 | Zinc finger (C2H2 type) family | Clusters 6+4 | VIT_04s0023g00440 falls within the confidence interval of a QTL for geraniol and nerol (Battilana *et al.* 2009, unpublished data) |
| Vv_10001872 | VIT_04s0023g01250 | Brassinosteroid signaling positive regulator (BZR1) | Profile: 2vs1,3vs1,5vs1down  Positive correlation with free OxB  Cluster 9  Correlated with Vv_10002561 (correlation > 0.99 between differential gene expression ratios)  Correlated with Vv_10003051, Vv_10007610, Vv_10009014 (correlation > 0.90 between microarray channel intensities) | The over-expression of *BZR1-D* in tomato leads to the up-regulation of carotenoid biosynthetic genes (including *DXS*) and increased carotenoid content in ripe fruit (Liu *et al.*, 2014)  Brassinosteroids are involved in grapevine berry development at early fruit development stages or around *veraison* (Fortes *et al.*, 2015)  VIT_04s0023g01250 falls within the confidence interval of a QTL for geraniol and nerol (Battilana *et al.* 2009, unpublished data) |
| Vv_10009982 | VIT_04s0023g01380 | Scarecrow-like | Profile: 2vs1,3vs1down  Positive correlation with bound rose oxide II  Cluster 4 | VIT_04s0023g01380 falls within the confidence interval of a QTL for geraniol and nerol (Battilana *et al.* 2009, unpublished data) and is predicted to be localized in the chloroplast |
| Vv_10002444  Vv_10012072$ | VIT_04s0023g02950 | Zinc finger (CCCH-type) family protein | Profile: 2vs1,3vs1,4vs1down  Clusters 4+5  Correlated with Vv_10003051, Vv_10007610, Vv_10009587, Vv_10012513 (correlation > 0.90 between microarray channel intensities)  Positive correlation with bound OxC  Cluster 4  Correlated with Vv_10003051, Vv_10006855, Vv_10007610, Vv_10009587, Vv_10012513 (correlation > 0.90 between microarray channel intensities) | VIT_04s0023g02950 falls within the confidence interval of a QTL for geraniol and nerol (Battilana *et al.* 2009, unpublished data) and is up-regulated in both flesh and skin throughout Muscat Hamburg ripening (Lijavetzky *et al.*, 2012) |
| Vv_10000933 | VIT_04s0023g03120 | Histone H3 | Profile: 3vs1,4vs1,5vs1up  Positive correlation with bound linalool, *trans*-8-HO-linalool, OxA, OxD, HO-diendiol I and II, rose oxide I and II  Cluster 4 | VIT_04s0023g03120 falls within the confidence interval of a QTL for geraniol and nerol (Battilana *et al.* 2009, unpublished data) |
| Vv_10003073$  Vv_10008371$  Vv_10010478$  Vv_10014421$  Vv_10006882$ | VIT_04s0044g00070  VIT_14s0066g01310 | RAB GDP dissociation inhibitor 1 ATGD1 (rab proteins geranylgeranyltransferase component a 2-like)  CAAX farnesyltransferase beta subunit | Positive correlation with bound linalool, *trans*-8-HO-linalool, OxA, OxC, OxD, HO-diendiol I and II, rose oxide I and II  Cluster 4  Vv_10003073 correlated with Vv_10000710 (correlation > 0.99 between differential gene expression ratios)  Vv_10014421 correlated with Vv_10000710 and Vv_10006996 (correlation > 0.99 between differential gene expression ratios)  Positive correlation with bound *trans*-8-HO-linalool, OxC  Cluster 4  Vv_10006882 correlated with Vv_10000710 and Vv_10006996 (correlation > 0.99 between differential gene expression ratios) | Protein prenylation is implicated in the regulation of TIA terpene mojety biosynthesis through the activation of *ORCA3* and MEP (*DXS*, *DXR*) gene expression in response to jasmonate (Courdavault *et al.*, 2009)  VIT_04s0044g00070 is predicted to be localized also in the chloroplast  VIT_14s0066g01310 falls within the confidence interval of a QTL for geraniol and nerol (Battilana *et al.* 2009, unpublished data) |
| Vv_10005693  Vv_10008803  Vv_10013342 | VIT_00s0214g00090 (chr4) | F-box protein PP2-B10 (Protein phloem protein 2-like B10) | Profile: 3vs1,4vs1,5vs1up (array), 4vs1up (qRT-PCR_2016)  Positive correlation with several monoterpenes  Clusters 4+6  Correlated with Vv_10009847 (correlation > 0.99 between differential gene expression ratios)  Correlated with Vv_10008655 (correlation > 0.90 between microarray channel intensities)  Profile: 3vs1,4vs1up  Positive correlation with free OxD  Cluster 8  Profile: 3vs1,4vs1up  Positive correlation with free OxD  Cluster 8  Correlated with Vv_10000443 and Vv_10001518 (correlation > 0.90 between microarray channel intensities) | VIT_00s0214g00090 falls within the confidence interval of a QTL for geraniol and nerol (Battilana *et al.* 2009, unpublished data) and is a putative ‘switch gene’ in the immature-to-mature transition during grapevine development (Palumbo *et al.*, 2014) |
| Vv_10002798$ | VIT_06s0004g03590 | TOE1 (target of eat1 1) related to apetala2 7 (ethylene signaling) | Cluster 9 | In Wen *et al.* (2015) XM_002284713.2 is coexpressed with the linalool/nerolidol synthase *VvCSLinNer* and the pinene synthase *VvPNaPin*  VIT_06s0004g03590 is significantly more expressed in the skin than in the pulp of Cabernet Sauvignon berries at 23 °Brix (Cramer *et al.*, 2014) |
| Vv_10002670$ | VIT_06s0004g04980 | Scarecrow transcription factor 14 (SCL14) | Cluster 9 | VIT_06s0004g04980 falls within the confidence interval of a QTL for nerol/geraniol (Battilana *et al.*, 2009) |
| Vv_10009860 | VIT_06s0004g07550 | Wound-induced protein WI12 | Profile: 2vs1,3vs1,4vs1,5vs1up (array), 2vs1,4vs1,5vs1up (qRT-PCR_2016)  Positive correlation with free OxC, HO-diendiol II, bound linalool, geraniol, nerol, OxA, citronellol, HO-diendiol I, rose oxide I  Clusters 9+4  Correlated with Vv_10008655 and Vv_10009347 (correlation > 0.95 and 0.90 between microarray channel intensities, respectively) | VIT_06s0004g07550 falls within the confidence interval of a QTL for nerol/geraniol (Battilana *et al.*, 2009) and is predicted to localize in the chloroplast  It is up-regulated in both flesh and skin throughout Muscat Hamburg ripening (Lijavetzky *et al.*, 2012) and is a putative ‘switch gene’ in the immature-to-mature transition during grapevine development (Palumbo *et al.*, 2014)  In VTCdb VIT_06s0004g07550 is coexpressed with the 9-cis-epoxycarotenoid dioxygenase *VvCCD4b* (VIT_02s0087g00930) |
| Vv_10003323$ | VIT_06s0009g01000 | Zinc finger (C3HC4-type ring finger) CIC7E11 | Cluster 9  Correlated with Vv_10003051 (correlation > 0.99 between differential gene expression ratios) | VIT_06s0009g01000 falls within the confidence interval of a QTL for nerol/geraniol (Battilana *et al.*, 2009) |
| Vv_10003411$ | VIT_06s0009g01570 | C2H2 zinc-finger protein SERRATE (SE) | Positive correlation with bound *trans*-8-HO-linalool, OxC  Cluster 4  Correlated with Vv_10000710 and Vv_10006996 (correlation > 0.99 between differential gene expression ratios) | VIT_06s0009g01570 falls within the confidence interval of a QTL for nerol/geraniol (Battilana *et al.*, 2009) |
| Vv_10009328$ | VIT_06s0009g02550 | Chaperonin | Cluster 6 | Involved in protein folding and associated to the GO term ‘Isopentenyl diphosphate biosynthetic process, methylerythritol 4-phosphate pathway’  VIT_06s0009g02550 falls within the confidence interval of a QTL for nerol/geraniol (Battilana *et al.*, 2009) |
| Vv_10012857$ | VIT_07s0005g01560 | PAPA-1 | Positive correlation with free geraniol  Cluster 6 | VIT_07s0005g01560 is annotated with the GO term ‘Chloroplast’ and falls within the confidence interval of a QTL for nerol/geraniol (Battilana *et al.*, 2009) |
| Vv_10014365$ | VIT_07s0005g02570 | WRKY DNA-binding protein 47 | Positive correlation with free OxB, OxD  Cluster 8, like free OxD | In VTCdb VIT_07s0005g02570 is coexpressed with *HMGR* (VIT_03s0038g04100) and a CYP76C (VIT_02s0025g04880)  VIT_07s0005g02570 is more highly up-regulated in the skin than in the flesh throughout Muscat Hamburg ripening (Lijavetzky *et al.*, 2012)  VIT_07s0005g02570 is significantly more expressed in the skin than in the pulp of Cabernet Sauvignon berries at 23 °Brix (Cramer *et al.*, 2014) |
| Vv_10013932 | VIT_07s0031g01320 | TGA-type basic leucine zipper protein TGA1.1 (jasmonate signaling) | Profile: 2vs1,3vs1,4vs1,5vs1down  Positive correlation with bound *trans*-8-HO-linalool, OxD, HO-diendiol II  Cluster 4  Correlated with Vv_10003051, Vv_10006855, Vv_10007610, Vv_10009014, Vv_10009587, Vv_10012513 (correlation > 0.90 between microarray channel intensities) | In Wen *et al.* (2015) XM_002280746.2 is coexpressed with *VvHMGR1* and the pinene synthase *VvPNaPin*  VIT_07s0031g01320 is significantly more expressed in the skin than in the pulp of Cabernet Sauvignon berries at 23 °Brix (Cramer *et al.*, 2014) |
| Vv_10009798  Vv_10010260  Vv_10014254 | VIT_07s0031g01930 | Myb TKI1 (TSL-kinase interacting protein 1) | Profile: 2vs1,3vs1,4vs1,5vs1up (Vv_10009798), 3vs1,4vs1,5vs1up (Vv_10010260 and Vv_10014254), 4vs1,5vs1up (qRT-PCR_2016)  Positive correlation with several monoterpenes (free linalool with high significance)  Clusters 4+6+9  Vv_10009798 correlated with Vv_10007610, Vv_10013486 (correlation > 0.99 between differential gene expression ratios) and Vv_10008655 (correlation > 0.90 between microarray channel intensities)  Vv_10010260 and Vv_10014254 correlated with Vv_10008655 and Vv_10009847 (correlation > 0.99 between differential gene expression ratios)  Vv_10014254 correlated with Vv_10008655 (correlation > 0.90 between microarray channel intensities) | VIT_07s0031g01930 is up-regulated in both flesh and skin throughout Muscat Hamburg ripening (Lijavetzky *et al.*, 2012) and is a candidate master regulator of grape berry maturation (Palumbo *et al.*, 2014) |
| Vv_10010364 | VIT_07s0104g01050 | Homeobox protein | Profile: 4vs1,5vs1up  Positive correlation with several monoterpenes  Clusters 9+4  Correlated with Vv_10008655 and Vv_10009347 (correlation > 0.90 between microarray channel intensities) | - |
| Vv_10004273$ | VIT_07s0191g00180 | Homeobox-leucine zipper protein ATHB-6 (ABA signaling) | Clusters 9+8 | In Wen *et al.* (2015) XM_003632428.1 is coexpressed with *VvHMGR1*, *VvHMGR3* and the pinene synthase *VvPNaPin* |
| Vv_10004635$ | VIT_08s0007g03910 | CTR1 serine/threonine protein kinase | Positive correlation with bound 4-terpineol  Clusters 6+2 | *LeCTR1* is involved in ethylene signaling downstream of ethylene receptor; it is up-regulated during tomato ripening (Leclercq *et al.*, 2002)  VIT_08s0007g03910 is predicted to be localized also in the chloroplast |
| Vv_10001158 | VIT_08s0007g05790 | Calmodulin | Profile: 2vs1,3vs1,4vs1,5vs1down  Cluster 9 | VIT_08s0007g05790 falls within the confidence interval of a QTL for geraniol and nerol (Battilana *et al.* 2009, unpublished data); it is significantly more expressed in the skin than in the pulp of Cabernet Sauvignon berries at 23 °Brix (Cramer *et al.*, 2014) and is predicted to be localized also in the chloroplast |
| Vv_10004111 | VIT_08s0007g05880 | Dehydration-induced protein (ERD15) (ABA signaling) | Profile: 2vs1,3vs1,4vs1,5vs1down  Cluster 9  Correlated with Vv_10002015, Vv_10003051, Vv_10006855, Vv_10007610, Vv_10009014, Vv_10009587, Vv_10012513 (correlation > 0.90 between microarray channel intensities) | Limited water availability promotes the accumulation of monoterpenes such as HO-trienol, linalool, nerol, and α-terpineol (Koundouras *et al.*, 2006; Savoi *et al.*, 2016)  VIT_08s0007g05880 falls within the confidence interval of a QTL for geraniol and nerol (Battilana *et al.* 2009, unpublished data) |
| Vv_10002459$ | VIT_08s0007g06530 | DnaJ homolog, subfamily B, member 6 (dnaj heat shock n-terminal domain-containing protein heat shock protein binding) | Cluster 6 | VIT_08s0007g06530 falls within the confidence interval of a QTL for geraniol and nerol (Battilana *et al.* 2009, unpublished data) and is predicted to be localized also in the chloroplast |
| Vv_10007395$ | VIT_08s0007g07060 | Unknown protein (Ubiquitin carboxyl-terminal hydrolase) | Clusters 4+9 | VIT_08s0007g07060 falls within the confidence interval of a QTL for geraniol and nerol (Battilana *et al.* 2009, unpublished data) and is predicted to be localized in the chloroplast |
| Vv_10000712$ | VIT_08s0007g07160 | Transcriptional co-activator | Positive correlation with several monoterpenes  Cluster 4  Correlated with Vv_10000443 and Vv_10011370 (correlation > 0.90 between microarray channel intensities) | VIT_08s0007g07160 falls within the confidence interval of a QTL for geraniol and nerol (Battilana *et al.* 2009, unpublished data) |
| Vv_10002372$ | VIT_08s0007g07670 | NAC domain containing protein 47 | Positive correlation with free OxB  Clusters 9+8  Correlated with Vv_10013486 (correlation > 0.90 between microarray channel intensities) | VIT_08s0007g07670 falls within the confidence interval of a QTL for geraniol and nerol (Battilana *et al.* 2009, unpublished data) and is a candidate master regulator of grape berry maturation (Palumbo *et al.*, 2014)  VIT_08s0007g07670 is up-regulated in both flesh and skin throughout Muscat Hamburg ripening (Lijavetzky *et al.*, 2012); in Sémillon it is up-regulated by noble rot, along with an increase in terpene biosynthesis (Blanco-Ulate *et al.*, 2015)  In VTCdb VIT_08s0007g07670 is coexpressed with *VvGT14* |
| Vv_10007880 | VIT_08s0007g08160 | Telomere repeat binding factor Like TRFL10 | Profile: 2vs1,3vs1,4vs1,5vs1up  Positive correlation with bound linalool, geraniol, *trans*-8-HO-linalool, OxA, OxD, HO-diendiol I, rose oxide I and II  Cluster 4 | VIT_08s0007g08160 falls within the confidence interval of a QTL for geraniol and nerol (Battilana *et al.* 2009, unpublished data) |
| Vv_10011970$ | VIT_08s0058g00200 | Transcription factor | Positive correlation with free nerol, bound *cis*-8-HO-linalool, OxD, *trans*-geranic acid, 7-HO-nerol, α-terpineol, HO-diendiol II  Clusters 4+6  Correlated with Vv_10004737 (correlation > 0.99 between differential gene expression ratios) | - |
| Vv_10001632  Vv_10007070$ | VIT_09s0054g01780  VIT_11s0016g02620 | HAC1 (P300/CBP acetyltransferase-related protein 2 gene)  Histone acetyltransferase HAG2 | Profile: 3vs1,4vs1,5vs1up  Positive correlation with bound *trans*-8-HO-linalool, rose oxide II  Cluster 4  Correlated with Vv_10006996 (correlation > 0.99 between differential gene expression ratios)  Positive correlation with bound OxC  Cluster 4  Correlated with Vv_10004183 (correlation > 0.99 between differential gene expression ratios) | Histone acetylation by histone acetyltransferases increases the accessibility of chromatin to the transcription machinery. In *Arabidopsis thaliana* many metabolic proteins are acetylated (Shen *et al.*, 2015) |
| Vv_10005633$ | VIT_10s0003g00190 | DNA-binding protein | Clusters 4+5 | VIT_10s0003g00190 falls within the confidence interval of a QTL for linalool, linalool/geraniol and linalool/nerol (Battilana *et al.*, 2009) and is predicted to be localized in the chloroplast |
| Vv_10003356$ | VIT_10s0003g00210 | Unknown | Clusters 6+4  Correlated with Vv_10000710 and Vv_10002691 (correlation > 0.90 between microarray channel intensities) | VIT_10s0003g00210 falls within the confidence interval of a QTL for linalool, linalool/geraniol and linalool/nerol (Battilana *et al.*, 2009) |
| Vv_10012485$ | VIT_10s0003g00270 | Homeobox-1 | Positive correlation with bound OxC  Cluster 4 | VIT_10s0003g00270 falls within the confidence interval of a QTL for linalool, linalool/geraniol and linalool/nerol (Battilana *et al.*, 2009) |
| Vv_10005202$ | VIT_10s0003g01770 | Heat shock transcription factor A4A | Positive correlation with bound *trans*-8-HO-linalool, OxD, *trans*-geranic acid, HO-diendiol II  Cluster 4  Correlated with Vv_10004737 (correlation > 0.99 between differential gene expression ratios) | In Wen *et al.* (2015) XM_002267135.2 is coexpressed with the linalool/nerolidol synthase *VvPNLinNer1* |
| Vv_10008513 | VIT_10s0003g03190 | RNA recognition motif (RRM)-containing | Profile: 2vs1,3vs1,4vs1,5vs1up (array)  Cluster 9  Correlated with Vv_10008655 and Vv_10009347 (correlation > 0.90 between microarray channel intensities) | VIT_10s0003g03190 falls within the confidence interval of a QTL for linalool, linalool/geraniol and linalool/nerol (Battilana *et al.*, 2009)  It is up-regulated in both flesh and skin throughout Muscat Hamburg ripening (Lijavetzky *et al.*, 2012)  In VTCdb VIT_10s0003g03190 is coexpressed with *HDR* (VIT_03s0063g02030) |
| Vv_10001832$ | VIT_10s0003g03710 | Cullin-4 | Positive correlation with free linalool and bound citronellol  Cluster 9  Correlated with Vv_10002561 (correlation > 0.90 between microarray channel intensities) | Cullin is part of the SCF complex that regulates the expression of jasmonate responsive genes (Devoto *et al.*, 2002)  JA treatment of grapes induces the expression of genes from the MVA and MEP pathways and the formation of volatile compounds, especially terpenes and norisoprenoids (D’Onofrio *et al.*, 2009; Gómez-Plaza *et al.*, 2012; May and Wüst, 2015) |
| Vv_10002850$ | VIT_00s0454g00030  (chr10) | Subtilisin protease | Positive correlation with free OxB  Cluster 9 | VIT_00s0454g00030 falls within the confidence interval of a QTL for linalool, linalool/geraniol and linalool/nerol (Battilana *et al.*, 2009); it is significantly more expressed in the skin than in the pulp of Cabernet Sauvignon berries at 23 °Brix (Cramer *et al.*, 2014) and is predicted to be localized in the chloroplast |
| Vv_10002430$ Vv_10012066$ | VIT_00s0463g00010 (chr10) | WRKY DNA-binding protein 20 | Positive correlation with free geraniol  Cluster 6 | VIT_00s0463g00010 falls within the confidence interval of a QTL for linalool, linalool/geraniol and linalool/nerol (Battilana *et al.*, 2009) |
| Vv_10009872 | VIT_00s0463g00020  (chr10) | Scarecrow transcription factor 5 (SCL5) | Profile: 2vs1,3vs1,4vs1,5vs1down  Clusters 5+6+4  Correlated with Vv_10003051, Vv_10007610, Vv_10009014, Vv_10009587, Vv_10012513 (correlation > 0.90 between microarray channel intensities) | VIT_00s0463g00020 falls within the confidence interval of a QTL for linalool, linalool/geraniol and linalool/nerol (Battilana *et al.*, 2009)  In Wen *et al.* (2015) XM_002272365.2 is coexpressed with the linalool/nerolidol synthase *VvCSLinNer* and the nerolidol synthase *VvNerL8* |
| Vv_10013923$ | VIT_00s0475g00040  (chr 10) | Myb family | Positive correlation with bound *trans*-8-HO-linalool, OxC  Cluster 4  Correlated with Vv_10000710 (correlation > 0.99 between differential gene expression ratios) | VIT_00s0475g00040 falls within the confidence interval of a QTL for linalool, linalool/geraniol and linalool/nerol (Battilana *et al.*, 2009) |
| Vv_10001911$ | VIT_11s0016g03290 | SNW domain-containing protein 1 SNW1 | Positive correlation with several monoterpenes  Cluster 4  Correlated with Vv_10011370 (correlation > 0.99 between differential gene expression ratios) | - |
| Vv_10004782$  Vv_10008760$ | VIT_12s0028g03130 | Proteasome 26S regulatory subunit S2 (RPN1) | Positive correlation with free geraniol, nerol, bound α-terpineol  Cluster 6  Correlated with Vv_10002691 and Vv_10000710 (correlation > 0.90 between microarray channel intensities)  Cluster 9  Correlated with Vv_10012513 (correlation > 0.99 between differential gene expression ratios) | VIT_12s0028g03130 falls within the confidence interval of a QTL for geraniol and linalool/geraniol (Battilana *et al.*, 2009) |
| Vv_10008596 | VIT_12s0028g03860 | Zinc finger (C3HC4-type ring finger) protein (RMA1) | Profile: 4vs1,5vs1up (array), 2vs1,3vs1,5vs1up (qRT-PCR_2016)  Positive correlation with bound OxC  Cluster 4  Correlated with Vv_10000710 (correlation > 0.99 between differential gene expression ratios) | VIT_12s0028g03860 falls within the confidence interval of a QTL for geraniol and linalool/geraniol (Battilana *et al.*, 2009) and is predicted to localize also in the chloroplast  VIT_12s0028g03860 is up-regulated in both flesh and skin throughout Muscat Hamburg ripening (Lijavetzky *et al.*, 2012) and is a candidate master regulator of grape berry maturation (Palumbo *et al.*, 2014)  In VTCdb VIT_12s0028g03860 is coexpressed with three CYP71s involved in monoterpenoid biosynthesis (VIT_01s0137g00540, VIT_01s0137g00550, VIT_01s0137g00560) (Ginglinger *et al.*, 2013) |
| Vv_10004541 | VIT_13s0019g00860 | Small heat-shock protein HSP17.5 Cytosolic class I | Profile: 2vs1,3vs1down  Clusters 9+4 | Involved in protein folding |
| Vv_10002554$ | VIT_13s0064g00640 | PHD finger transcription factor | Cluster 9 | VIT_13s0064g00640 falls within the confidence interval of a QTL for geraniol and nerol (Doligez *et al.*, 2006)  In VTCdb VIT_13s0064g00640 is coexpressed with *GPPS* (VIT_06s0009g03090) |
| Vv_10000441$ | VIT_13s0064g00920 | Zinc finger (CCCH-type) family protein | Positive correlation with several bound monoterpenes  Cluster 4  Correlated with Vv_10000443 and Vv_10011370 (correlation > 0.99 between differential gene expression ratios)  Correlated with Vv_10000710 (correlation > 0.90 between microarray channel intensities) | VIT_13s0064g00920 falls within the confidence interval of a QTL for geraniol and nerol (Doligez *et al.*, 2006) |
| Vv_10010154$ | VIT_14s0083g00940 | Auxin-independent growth promoter | Positive correlation with free geraniol, bound α-terpineol  Cluster 6 | VIT_14s0083g00940 is a putative ‘switch gene’ in the immature-to-mature transition during grapevine development (Palumbo *et al.*, 2014) and is predicted to localize also in the chloroplast |
| Vv_10012816$ | VIT_14s0219g00200 | Pentatricopeptide (PPR) repeat-containing protein | Cluster 9  Correlated with Vv_10000443, Vv_10009847 and Vv_10011974 (correlation > 0.90 between microarray channel intensities) | PPR proteins take part in the post-transcriptional regulation of organelle gene expression. For example, the post-transcriptional regulation of mitochondrial RNA by the PPR protein LOI1 is likely involved in isoprenoid biosynthesis *via* both the MVA and MEP pathways (Kobayashi *et al.*, 2007)  VIT_14s0219g00200 falls within the confidence interval of a QTL for geraniol and nerol (Battilana *et al.* 2009, unpublished data); it is significantly more expressed in the skin than in the pulp of Cabernet Sauvignon berries at 23 °Brix (Cramer *et al.*, 2014) and is predicted to be localized in chloroplast |
| Vv_10010750 | VIT_16s0050g01150 | Heat shock protein 90-1 | Profile: 3vs1down  Clusters 9+7 | Involved in protein folding |
| Vv_10011630$ | VIT_16s0148g00420  VIT_00s2365g00010  VIT_00s0258g00040  VIT_16s0148g00260  VIT_16s0050g02740  VIT_16s0039g01210  VIT_00s2248g00010  and others | Rust resistance kinase Lr10  Kinase  Ser/Thr receptor-like kinase1  Ser/Thr receptor-like kinase1  Receptor-like protein kinase  Ser/Thr receptor-like kinase1  Rust resistance kinase Lr10 | Positive correlation with bound 4-terpineol  Clusters 6+5  Correlated with Vv_10002691 (correlation > 0.90 between microarray channel intensities) | Based on the IASMA annotation, Vv_10011630 falls within the confidence interval of a QTL for nerol/geraniol on chromosome 1 (Battilana *et al.*, 2009)  In VTCdb the corresponding V1 gene predictions are coexpressed with genes that determine a significant enrichment in the category ‘Isoprenoid metabolic process’, including ‘ 1-deoxy-D-xylulose-5-phosphate synthase activity’ and ‘S-linalool synthase activity’  VIT_00s2365g00010 has a constant expression in the skin and is repressed in the flesh throughout Muscat Hamburg ripening (Lijavetzky *et al.*, 2012)  VIT_16s0148g00420, VIT_00s2365g00010, VIT_00s0258g00040, VIT_16s0148g00260, VIT_16s0050g02740, VIT_16s0039g01210 and VIT_00s2248g00010 are significantly more expressed in the skin than in the pulp of Cabernet Sauvignon berries at 23 °Brix (Cramer *et al.*, 2014)  VIT_00s2365g00010 and VIT_00s2248g00010 are predicted to be localized also in the chloroplast |
| Vv_10000260 | VIT_18s0001g03580 | Ubiquitin-fold modifier 1 precursor | Profile: 4vs1,5vs1up  Positive correlation with bound linalool, *trans*-8-HO-linalool, OxA, OxD, HO-diendiol I and II, rose oxide I and II  Cluster 4 | VIT_18s0001g03580 is predicted to be localized also in the chloroplast |
| Vv_10003319 | VIT_18s0001g15270 | SCL1 (scarecrow-like 1) | Profile: 2vs1,3vs1,4vs1,5vs1down  Cluster 6  Correlated with Vv_10007610 and Vv_10009014 (correlation > 0.90 between microarray channel intensities) | In Wen *et al.* (2015) XM_002266379.2 is coexpressed with the linalool/nerolidol synthase *VvCSLinNer* and the nerolidol synthase *VvNerL8*  VIT_18s0001g15270 is predicted to be localized in the chloroplast |
| Vv_10002436$ | VIT_19s0014g00860 | Zinc finger (FYVE type) | Positive correlation with free nerol, bound *trans*-geranic acid, 7-HO-nerol, α-terpineol  Cluster 6 | - |
| Vv_10013790$ | VIT_19s0090g01430 | EDM2 | Positive correlation with free nerol, bound *cis*-8-HO-linalool, OxD, *trans*-geranic, 7-HO-nerol  Clusters 6+4 | In VTCdb VIT_19s0090g01430 is coexpressed with three CYP71s involved in monoterpenoid biosynthesis (VIT_01s0137g00540, VIT_01s0137g00550, VIT_01s0137g00560) (Ginglinger *et al.*, 2013) |

**References (not included in the main text)**

Boss, P. K., Sensi, E., Hua, C., Davies, C., and Thomas, M. R. (2002). Cloning and characterisation of grapevine (*Vitis vinifera* L.) MADS-box genes expressed during inflorescence and berry development. *Plant Sci.* 162, 887-895. doi: 10.1016/S0168-9452(02)00034-1

Courdavault, V., Burlat, V., St-Pierre, B., and Giglioli-Guivarc’h, N. (2009). Proteins prenylated by type I protein geranylgeranyltransferase act positively on the jasmonate signalling pathway triggering the biosynthesis of monoterpene indole alkaloids in *Catharanthus roseus*. *Plant Cell Rep.* 28, 83-93. doi: 10.1007/s00299-008-0610-1

Fan, J., Chen, C., Yu, Q., Li, Z. G., and Gmitter, F. G. Jr. (2010). Characterization of three terpenoid glycosyltransferase genes in 'Valencia' sweet orange (*Citrus sinensis* L. Osbeck). *Genome* 53, 816-823. doi: 10.1139/g10-068

Grimplet, J., Van Hemert, J., Carbonell-Bejerano, P., Díaz-Riquelme, J., Dickerson, J., Fennell, A., et al. (2012). Comparative analysis of grapevine whole-genome gene predictions, functional annotation, categorization and integration of the predicted gene sequences. *BMC Res. Notes* 5, 213. doi: 10.1186/1756-0500-5-213

Kärblane, K., Gerassimenko, J., Nigul, L., Piirsoo, A., Smialowska, A., Vinkel, K., et al. (2015). ABCE1 is a highly conserved RNA silencing suppressor. *PLoS ONE* 10, e0116702. doi: 10.1371/journal.pone.0116702

Kobayashi, K., Suzuki, M., Tang, J., Nagata, N., Ohyama K., Seki, H., et al. (2007). LOVASTATIN INSENSITIVE 1, a novel pentatricopeptide repeat protein, is a potential regulatory factor of isoprenoid biosynthesis in *Arabidopsis*. *Plant Cell Physiol.* 48, 322-331. doi: 10.1093/pcp/pcm005

Leclercq, J., Adams-Phillips, L. C., Zegzouti, H., Jones, B., Latché, A., Giovannoni, J. J., et al. (2002). *LeCTR1*, a tomato *CTR1*-like gene, demonstrates ethylene signaling ability in *Arabidopsis* and novel expression patterns in tomato. *Plant Physiol.* 130, 1132-1142. doi: 10.1104/pp.009415

Nagashima S, Tomo S, Yoshikawa T. Data from: cDNA encoding monoterpene glucosyltransferase from suspension culture of *Eucalyptus perrinian*. UniProt Digital Repository (2005) <http://www.uniprot.org/uniprot/Q5CD68> and <http://www.uniprot.org/uniprot/Q5CD69>

Schäfer, E., and Bowler, C. (2002). Phytochrome-mediated photoperception and signal transduction in higher plants. *EMBO Rep.* 3, 1042-1048. doi: 10.1093/embo-reports/kvf222

Whitaker, B. D., and Saftner, R. A. (2000). Temperature-dependent autoxidation of conjugated trienols from apple peel yields 6-methyl-5-hepten-2-one, a volatile implicated in induction of scald. *J. Agric. Food Chem.* 48, 2040-2043. doi: 10.1021/jf991107c
