# Supplementary material for: Drawing Links from Transcriptome to Metabolites: The Evolution of Aroma in the Ripening Berry of Moscato Bianco (Vitis vinifera L.)
Source: Front Plant Sci. 2017 May 16;8:780. doi: 10.3389/fpls.2017.00780 (PMC5432621; doi:10.3389/fpls.2017.00780)
Supplement: Supplementary file 10 [file Table10.docx]

**Supplementary Table S10:** Candidate genes for the biosynthesis of carotenoids/C_13_-norisoprenoids, phenylpropanoids/benzenoids and C_6_ aliphatic compounds (fatty acid derivatives). Only genes with at least one supporting evidence from the present work (differential expression consistent with metabolite accumulation, correlation with specific metabolites, membership to clusters or biclusters (BCs) harboring the metabolites under examination) are listed. Probes marked with $ were not differentially expressed during Moscato Bianco berry ripening (with a cut-off of 2-fold change and a false discovery rate < 1%). Functional annotation was derived from Additional file 1 by Grimplet *et al.* (2012).

| **Probe ID** | **V1 gene prediction** | **Functional annotation** | **Evidences from the present work** | **Evidences from the literature** |
| --- | --- | --- | --- | --- |
| **Carotenoids/C_13_-norisoprenoids** | | | | |
| Vv_10000833 | VIT_12s0028g00960 | Phytoene synthase, chloroplast precursor (PSY) | Profile: 2vs1,3vs1,4vs1,5vs1up  Cluster 2 | This enzyme catalyzes the condensation of GGPP to form phytoene, the basic precursor of all carotenoids. PSY is generally accepted as being the most important regulatory enzyme in the pathway |
| Vv_10009139  Vv_10013477 | VIT_03s0038g02680 | ζ-carotene desaturase | Profile: 3vs1,5vs1down  Clusters 3+1  Profile: 3vs1,5vs1down  Clusters 7+3+1 | Involved in lycopene biosynthesis |
| Vv_10008866 | VIT_14s0030g01740 | ζ-carotene desaturase ZDS1 | Profile: 2vs1,3vs1,4vs1up  Cluster 3 | Involved in lycopene biosynthesis VIT_14s0030g01740 is up-regulated at E-L 36 compared to E-L 35 also in Touriga Nacional (Agudelo-Romero *et al*., 2013) |
| Vv_10002645$ | VIT_11s0016g01880 | Lycopene epsilon cyclase, chloroplast precursor (eLCY) | Cluster 3 | - |
| Vv_10003598 | VIT_16s0050g01090 | β-carotene hydroxylase (BCH) | Cluster 3 | This enzyme catalyzes the hydroxylation reaction from β-carotene to zeaxanthin  VIT_16s0050g01090 is down-regulated at E-L 36 compared to E-L 35 also in Trincadeira, Touriga Nacional and Aragonês (Agudelo-Romero *et al.*, 2013) |
| Vv_10011535  Vv_10000915 | VIT_06s0009g01670  VIT_07s0031g00620 | Forkhead-associated domain-containing protein (zeaxanthin epoxidase)  Zeaxanthin epoxidase (ZEP) (ABA1) | Profile: 4vs1,5vs1down  Cluster 3  Profile: 3vs1,4vs1,5vs1down  Cluster 1 | This enzyme converts zeaxanthin into antheraxanthin and subsequently violaxanthin  VIT_07s0031g00620 is down-regulated at E-L 36 compared to E-L 35 also in Trincadeira, Touriga Nacional and Aragonês (Agudelo-Romero *et al.*, 2013) |
| Vv_10003015 | VIT_02s0087g00910 | 9-*cis-*epoxycarotenoid dioxygenase (*VvCCD4a*) | Profile: 5vs1up | *VvCCD4a* is up-regulated during berry ripening in Cabernet Sauvignon, Chardonnay and Pinotage (Guillaumie *et al.*, 2011; Young *et al.*, 2012; Lashbrooke *et al.*, 2013) |
| Vv_10006805$  Vv_10002841$ | VIT_10s0003g04360  VIT_10s0003g03710 | DDB1A (UV-damaged DNA-binding protein 1A)  Cullin-4 | Cluster 6 (increasing trend), consistent with the decrease of carotenoids during berry ripening  Cluster 4 (increasing trend), consistent with the decrease of carotenoids during berry ripening | DDB1/HP1 and CUL4 are important components of an E3 complex which negatively regulates plastid size and plastid number, and subsequently carotenoid accumulation (Wang *et al.*, 2008) |
| **Phenylpropanoids/benzenoids** | | | | |
| Vv_10000249$  Vv_10005246$ | VIT_16s0022g01440 | Transketolase | Positive correlation with free benzaldehyde  Cluster 3, like free benzaldehyde | This enzyme produces E4P, which is a precursor of the shikimate pathway  VIT_16s0022g01440 is predicted to be localized in the chloroplast |
| Vv_10003556  Vv_10003500$  Vv_10008290$ | VIT_17s0000g08200  VIT_18s0001g05060 | Phosphoglycerate/bisphosphoglycerate mutase  2,3-bisphosphoglycerate-dependent phosphoglycerate mutase | Profile: 3vs1,4vs1,5vs1up  Cluster 9, partially like bound benzyl alcohol  Cluster 9, partially like bound benzyl alcohol  Cluster 5 | This enzyme produces E4P, which is a precursor of the shikimate pathway |
| Vv_10004959  Vv_10001608$ | VIT_02s0025g04920  VIT_05s0020g00740 | Phosphate translocator protein2  Phosphate translocator | Profile: 2vs1,3vs1up  Positive correlation with free methyl salicylate (data not shown)  Cluster 2, like free benzyl alcohol and 2-phenylethanol  Cluster 6, partially like free 2-phenylethanol | This translocator may import cytosolic PEP, which is a precursor of the shikimate pathway |
| Vv_10004275  Vv_10010006$ | VIT_00s0391g00070 | 3-deoxy-D-arabino-heptulosonate 7-phosphate synthase | Profile: 2vs1,3vs1,4vs1up  Cluster 8, like bound benzaldehyde  Cluster 3, like free benzaldehyde | First enzyme of the shikimate pathway, which plays a key role in controlling carbon flux into the pathway (Dudareva *et al.*, 2013) |
| Vv_10005567$ | VIT_04s0023g03820 | 3-dehydroquinate synthase | Cluster 3, like free benzaldehyde | Second enzyme of the shikimate pathway |
| Vv_10008119 | VIT_14s0030g00660 | Shikimate dehydrogenase | Profile: 3vs1,4vs1,5vs1up  Cluster 9, partially like bound benzyl alcohol | Forth enzyme of the shikimate pathway |
| Vv_10007786$  Vv_10009338$ | VIT_14s0108g00010 | Isochorismate synthase 1, chloroplast precursor | Clusters 3+2, like free benzyl alcohol, benzaldehyde and 2-phenylethanol (methyl salicylate was excluded from clustering) | Enzyme involved in the formation of methyl salicylate |
| Vv_10000965 | VIT_04s0023g02290  VIT_04s0023g02280 and others | S-adenosyl-L-methionine:salicylic acid carboxyl methyltransferase | Profile: 2vs1,3vs1,4vs1,5vs1down  Cluster 3, like free benzaldehyde (methyl salicylate was excluded from clustering) | Enzyme involved in the synthesis of methyl salicylate  *CbSAMT* catalyzes the formation of methyl salicylate (Ross *et al.*, 1999)  Expression of *AtBSMT1* correlates with induced methyl salicylate emission (Chen *et al.*, 2003)  VIT_04s0023g02280 is down-regulated at E-L 36 vs E-L 35 in three aromatic varieties (Agudelo-Romero *et al.*, 2013). It may play an important role in specific aroma production at early stages of berry development (Effmert *et al.*, 2005) |
| Vv_10009307  Vv_10013578  Vv_10001306 | VIT_12s0059g00750  VIT_06s0061g01300 | Prephenate dehydratase | Profile: 2vs1,3vs1,4vs1up  Positive correlation with free methyl salicylate (data not shown)  Cluster 3, like free benzaldehyde  Profile: 2vs1,3vs1,4vs1,5vs1down  Cluster 1 | Enzyme of the phenylalanine pathway |
| Vv_10002167 | VIT_19s0014g02190 | Tyrosine aminotransferase | Profile: 2vs1,3vs1,4vs1,5vs1up  Positive correlation with bound 2-phenylethanol  Cluster 4, like bound 2-phenylethanol | CmArAT1 converts phenylalanine to its corresponding α-keto acids, which are precursors for various aromatic compounds, including phenylacetaldehyde (Gonda *et al.*, 2010; Sun *et al.*, 2016) |
| Vv_10000978 | VIT_16s0039g01120 | Phenylalanin ammonia-lyase | Profile: 3vs1down  Cluster 7+9, partially like bound benzyl alcohol | Enzyme for the formation of cinnamic acid, from which benzenoids can be generated |
| Vv_10004786  Vv_10005992$  Vv_10009527 | VIT_02s0025g03660  VIT_16s0039g02040 | 4-coumarate-CoA ligase  4-coumarate-CoA ligase 3 | Profile: 2vs1,3vs1,4vs1,5vs1down  Cluster 7  Positive correlation with bound methyl salicylate (data not shown)  Cluster 7  Profile: 5vs1down  Clusters 3+7, like free benzaldehyde | Enzyme of the CoA-dependent, β-oxidative pathway for the formation of benzenoids |
| Vv_10011089$ | VIT_02s0234g00080 | Enoyl-CoA hydratase/isomerase | Cluster 2, like free benzyl alcohol | Enzyme of the CoA-dependent, β-oxidative pathway for the formation of benzenoids |
| Vv_10006902 | VIT_01s0026g00220 | Aldehyde dehydrogenase 1 precursor | Profile: 2vs1,3vs1,4vs1,5vs1up  Cluster 3, like free benzaldehyde | Enzyme potentially involved in the non-β-oxidative pathway for the formation of benzenoids |
| Vv_10006719  Vv_10007868$ | VIT_14s0066g01090 | Myb domain protein 24 | Profile: 5vs1up  Cluster 4, like bound benzyl alcohol and 2-phenylethanol | VIT_14s0066g01090 shares homology with *NtMyb3O5*, FC0568, *PhEOBI* and *PhEOBII*  *NtMyb3O5* activates *NtPAL2* promoter (Sablowski *et al.*, 1995)  FC0568 is a scent-related candidate gene in rose (Guterman *et al.*, 2002)  *PhEOBI* and *PhEOBII* have a key regulatory role in the biosynthesis of phenylpropanoid volatiles (Spitzer-Rimon *et al.,* 2010 and 2012) |
| **Volatile fatty acid derivatives** | | | | |
| Vv_10004567$ | VIT_14s0128g00780 | Lipoxygenase | Cluster 2 | Putative 9-lipoxygenase (*VvLOXC*) with gradual decrease in expression across Sauvignon Blanc berry development (Podolyan *et al.*, 2010) |
| Vv_10005247  Vv_10009290 | VIT_12s0059g01060 | Hydroperoxide lyase (HPL1) | Cluster 9, like free hexanol  Clusters 2+7, like free and bound *cis*-3-hexen-1-ol | Enzyme of the lipoxygenase pathway  VIT_12s0059g01060 is up-regulated at *veraison* and decreases at full ripen stage, which roughly corresponds to the accumulation of its volatile C_6_ aldehyde products in Trincadeira and Cabernet Sauvignon grapes; it catalyzes the cleavage of 13-hydroperoxides producing C_6_ aldehydes *in vitro* (Fortes *et al.*, 2011; Zhu *et al.*, 2012)  The expression of *VvHPL1* parallels with the levels of C_6_ aldehydes and alcohols in four different varieties (Qian *et al.*, 2016) |
| Vv_10009117 | VIT_07s0005g04600 | Alcohol dehydrogenase class III | Profile: 3vs1,4vs1,5vs1down  Cluster 1  BCs 115 (like bound *cis*-3-hexen-1-ol) and 125 (like free *cis*-3-hexen-1-ol) | Potentially involved in the reduction of C_6_ and C_9_ aldehydes to alcohols |
