# Supplementary material for: Drawing Links from Transcriptome to Metabolites: The Evolution of Aroma in the Ripening Berry of Moscato Bianco (Vitis vinifera L.)
Source: Front Plant Sci. 2017 May 16;8:780. doi: 10.3389/fpls.2017.00780 (PMC5432621; doi:10.3389/fpls.2017.00780)
Supplement: Supplementary file 11 [file DataSheet1.docx]

Supplementary Material

Drawing links from transcriptome to metabolites: the evolution of aroma in the ripening berry of Moscato Bianco (*Vitis vinifera* L.)

**Laura Costantini*, Christian Kappel, Massimiliano Trenti, Juri Battilana, Francesco Emanuelli, Maddalena Sordo, Marco Moretto, Céline Camps, Roberto Larcher, Serge Delrot, Maria Stella Grando**

*** Correspondence:** Laura Costantini: laura.costantini@fmach.it

**1 Supplementary methods**

**1.1 Method S1: Metabolic analysis**

The juice obtained by crushing 100 g of frozen berries was submitted to a hydrophobic cross-linked polystyrene co-polymer (XAD-2 resin with particle size 0.2-0.25 mm, SERVA Electrophoresis GmbH, Heidelberg, Germany). The free fraction was eluted with 100 mL of pentane-dichloromethane (2:1); the eluate was dried over anhydrous sodium sulphate and concentrated to 0.5 mL by evaporation, then it was stored at -20 °C until HRGC-MS analysis. The bound fraction was eluted with 100 mL of methanol-ethyl acetate (9:1) and concentrated to dryness in Rotavapor evaporator (BŰCHI, Flawil, Switzerland) before dissolution in 5 mL of citrate-phosphate buffer (pH 5.0). The bound forms were hydrolyzed to aglycons after reaction with AR2000 enzyme (Gist Brocades, Delft, The Netherlands); the hydrolysates were extracted three times with 10 mL of pentane-dichloromethane (2:1) and the extract was concentrated to 0.5 mL by distillation before HRGC-MS analysis.

HRGC-MS analysis was performed using a PerkinElmer gas chromatograph with a Turbomass Gold mass spectrometer (Perkin Elmer, Norwalk, Connecticut, USA) equipped with a DBWax fused silica column (I. D. 60 m x 0.32 mm, film thickness 0.5 µM, J&W Scientific, Folsom, California, USA). Helium was used as carrier gas with a constant flow rate of 1.2 ml min^-1^. The oven program was as follows: 50 °C for 10 min, 10 °C min^-1^ to 60 °C, 60 °C for 30 s, 2.5 °C min^-1^ to 200 °C, 200 °C for 10 min, 10 °C min^-1^ to 250 °C, 250 °C for 4 min. Detector temperature was set to 220 °C. Mass spectra were scanned in the range m/e 30-300 amu; total ion chromatograms (TIC) profiles were obtained. Metabolite quantification was referred to the internal standard 1-heptanol with response factor (RF) = 1.

For repeatability tests a pool of 1.2 kg berries was taken from Moscato Bianco at technological maturity (18 °Brix) in 2006. To evaluate the variance due to the random sub-sampling of berries, six samples were prepared by collecting 100 g of berries from the homogeneous pool and then applying the analysis method previously described. The remaining 600 g of berries were crushed on N_2_ air conditioning and maintained on ice to avoid metabolite oxidation. The resulting juice was quickly distributed in 20 falcon tubes (about 30 g each) and stored at -20 °C. This set of reference samples was used to assess the repeatability of the method during sample preparation (Supplementary Table S1).

**1.2 Method S2: Microarray analysis**

A dye-swap experimental design was adopted, in view of the technical variability intrinsic to the AROS microarrays and in agreement with several papers employing the same approach (Rizzini *et al.*, 2009; Samuelian *et al.*, 2009; Camps *et al.*, 2010; Guillaumie *et al.*, 2011; Perrone *et al.*, 2012; Marchive *et al.*, 2013).

Fluorescently (Cy3/Cy5) labelled antisense RNA (aRNA) targets were obtained using the Amino Allyl MessageAmp^TM^ II aRNA Amplification Kit (Ambion, Austin, Texas, USA) according to manufacturer’s instructions. Six hundred pmol Cy3- and Cy5-labelled aRNA were pooled together, fragmented and adjusted to 100 µL with the hybridization buffer. Just before hybridization, the oligos were fixed on the chip by exposure to 100 MJ of UV light in a UV Stratalinker 2400 crosslinker (Stratagene, La Jolla, California, USA). The slides were then soaked twice in 0.2% sodium dodecyl sulfate (SDS) for 1 min. The probe solution was denatured at 100 °C for 1 min, cooled on ice for 2 min, stabilized at 37 °C for 5 min and injected into the hybridization chamber of the HS 4800 Hybridization Station (Tecan, Durham, North Carolina, USA). The slides were incubated at 37 °C for 16 h with medium agitation and then washed sequentially at 30 °C in 1X saline-sodium citrate (SSC)/0.1% SDS for 1 min 3 times, in 0.1X SSC/0.1% SDS for 1 min 3 times and finally in 0.1X SSC for 30 s. Washed slides were quickly dried with nitrogen and immediately scanned at 532 (Cy3) and 635 (Cy5) nm with GenePix 4000B fluorescence reader (Molecular Devices, Sunnyvale, California, USA) using GenePix 4.0 software. Parameters were fixed as follows: 400 V at 532 nm and 460 V at 635 nm for PTM (photomultiplier), 100% power, 5 for pixel size and 3 lines to average.

**1.3 Method S3: Real-time analysis**

After digestion with Deoxyribonuclease I (Invitrogen, Carlsbad, California, USA), 1 µg of total RNA from each sample (three biological- and two technical replicates) was reverse transcribed using the Invitrogen SuperScript™ III Reverse Transcriptase followed by the RNase H step according to the manufacturer’s instructions. Each PCR reaction contained 2 µl of diluted cDNA (~ 10 ng of template), 10 µl of 2X LightCycler® 480 SYBR Green I Master Mix (Roche Applied Science, Mannheim, Germany) and 0.6 µM of each primer in a final volume of 20 µl. The two best-performing housekeeping genes (elongation factor 1-α and glyceraldehyde-3-phosphate dehydrogenase) were selected from a set of five genes by using the geNorm software and a gene expression normalization factor based on their geometric average was calculated (Reid *et al.*, 2006). Reaction plates included a non-template negative control and both reference and target genes. PCR cycling conditions were as follows: pre-incubation at 95 °C for 5 min; amplification over 40 cycles at 95 °C for 15 s, primer dependent-annealing temperature (~ 60 °C) for 30 s and 72 °C for 10 s. Finally, a post-PCR melting curve analysis was performed to verify the specificity of cDNA amplification.

Primer efficiencies (E) were obtained by means of serial dilution standard curves for each gene. E was calculated using the equation E=10^(-1/slope)^ and the slopes given by LightCycler 480 software (Roche Applied Science). The relative expression of the target genes at each time point from 2 to 5 with respect to the first time point was established according to the relative quantification model published by Hellemans *et al.* (2007). The expression ratio results were tested for significance (p-value < 0.001) by a Student’s t-test.

**References (not included in the main text)**

Camps, C., Kappel, C., Lecomte, P., Léon, C., Gomès, E., Coutos-Thévenot, P., et al. (2010). A transcriptomic study of grapevine (*Vitis vinifera* cv. Cabernet-Sauvignon) interaction with the vascular ascomycete fungus *Eutypa lata*. *J. Exp. Bot.* 61, 1719-1737. doi: 10.1093/jxb/erq040

Guillaumie, S., Fouquet, R., Kappel, C., Camps, C., Terrier, N., Moncomble, D., et al. (2011). Transcriptional analysis of late ripening stages of grapevine berry. *BMC Plant Biol.* 11, 165. doi: 10.1186/1471-2229-11-165

Hellemans, J., Mortier, G., De Paepe, A., Speleman, F., and Vandesompele, J. (2007). qBase relative quantification framework and software for management and automated analysis of real-time quantitative PCR data. *Genome Biol.* 8, R19. doi: 10.1186/gb-2007-8-2-r19

Marchive, C., Léon, C., Kappel, C., Coutos-Thévenot, P., Corio-Costet, M. F., Delrot, S., et al. (2013). Over-expression of *VvWRKY1* in grapevines induces expression of jasmonic acid pathway-related genes and confers higher tolerance to the downy mildew. *PLoS One* 8, e54185. doi: 10.1371/journal.pone.0054185

Perrone, I., Pagliarani, C., Lovisolo, C., Chitarra, W., Roman, F., and Schubert, A. (2012). Recovery from water stress affects grape leaf petiole transcriptome. *Planta* 235, 1383-1396. doi: 10.1007/s00425-011-1581-y

Reid, K. E., Olsson, N., Schlosser, J., Peng, F., and Lund, S. T. (2006). An optimized grapevine RNA isolation procedure and statistical determination of reference genes for real-time RT-PCR during berry development. *BMC Plant Biol.* 6, 27. doi: 10.1186/1471-2229-6-27

Rizzini, F. M., Bonghi, C., and Tonutti, P. (2009). Postharvest water loss induces marked changes in transcript profiling in skins of wine grape berries. *Postharvest Biol. Technol.* 52, 247-253. doi:10.1016/j.postharvbio.2008.12.004

Samuelian, S. K., Camps, C., Kappel, C., Simova, E. P., Delrot, S., and Colova (Tsolova) V. M. (2009). Differential screening of overexpressed genes involved in flavonoid biosynthesis in North American native grapes: ‘Noble’ *muscadinia* var. and ‘Cynthiana’ *aestivalis* var. *Plant Sci.* 177, 211-221. doi:10.1016/j.plantsci.2009.05.013

**2 Supplementary discussion**

**2.1 Carotenoids/C_13_-norisoprenoids**

C_13_-norisoprenoids contribute complex aroma to wines, including floral and fruity attributes (Ebeler and Thorngate, 2009; Mendes-Pinto, 2009; Robinson *et al.*, 2014; Black *et al.*, 2015). In particular, the compounds quantified in this work, 3-hydroxy-*β*-damascone, 3-oxo-*α*-ionol and 6-methyl-5-hepten-2-one confer tea and tobacco, tobacco, green fruit and pear notes, respectively. 3-hydroxy-β-damascone and 3-oxo-α-ionol have a negligible olfactory impact in wine, however it has been suggested that 3-hydroxy-*β*-damascone acts as a precursor of the potent odor-active β-damascenone. C_13_-norisoprenoids are present in grape leaves and berries mainly under glycoconjugated forms. Therefore, they are non-volatile and odorless until release by enzymatic or acid hydrolysis during fermentation. Glycosylated C_13_-norisoprenoids derive from the degradation of carotenoids. In ripening grape berries carotenoids are synthesized from the first stage of fruit formation to *veraison*, and then their levels decrease while the levels of C_13_-norisoprenoids increase (Baumes *et al.*, 2002). The genes and enzymes of the carotenoid metabolic pathway in *Vitis vinifera* have been described by Young *et al.* (2012). The first committed step in carotenoid biosynthesis is the production of the 40-carbon phytoene from condensation of two geranylgeranyl pyrophosphate (GGPP) molecules, which is catalyzed by the enzyme phytoene synthase (PSY). The production of all *trans*-lycopene from phytoene requires a complex set of four reactions involving phytoene desaturase (PDS), ζ-carotene isomerase (Z-ISO), ζ-carotene desaturase (ZDS) and carotenoid isomerase (CRTISO). Carotenoid biosynthesis bifurcates after lycopene to produce ε- and β-carotenoids by enzymatic activity of the two lycopene cyclases, eLCY and bLCY, and this branch point has a major regulatory role in modulating the ratio of the most abundant carotenoid, lutein, to the β-carotenoids. The three C_13_-norisoprenoids analyzed in this work, 3-hydroxy-β-damascone, 3-oxo-α-ionol and 6-methyl-5-hepten-2-one derive from neoxanthin, lutein and lycopene, respectively (Baumes *et al.*, 2002; Lashbrooke *et al.*, 2013). In agreement with the decrease of carotenoid concentration following *veraison*, we found probes for the carotenoid biosynthetic genes *PSY*, *ZDS*, e*LCY*, *BCH* and *ZEP* in clusters characterized by a decreasing trend during berry ripening (clusters 1, 2, 3 and 7) (Supplementary Table S10). The essential steps for the production of C_13_-norisoprenoids consist of the initial regioselective dioxygenase cleavage, the subsequent enzymatic transformation(s) (by oxidases and reductases) of the primary degradation product, and the formation of the flavorless glycoside (Baumes *et al.*, 2002; Winterhalter and Rouseff, 2002; Mathieu *et al.*, 2009). Four subfamilies of plant carotenoid cleavage dioxygenases have been identified: CCD1, CCD4, CCD7, and CCD8 and the grapevine orthologs have been characterized (Mathieu *et al.*, 2005; Young *et al.*, 2012; Lashbrooke *et al.*, 2013). In particular, *VvCCD1*, *VvCCD4a* and *VvCCD4b* were shown to be capable of *in vitro* catalysis of the cleavage of C_40_ carotenoid substrates at the 9, 10 (9’, 10’) (ζ-carotene, β-carotene and/or ε-carotene) and at the 5, 6 (5’, 6’) (lycopene) double bond position to release the corresponding C_13_ apocarotenoid products. Based on their expression and compartmentalization *in planta*, it was suggested that *VvCCD4a* and *VvCCD4b* are primarily responsible for the cleavage of plastidial carotenoids. Conversely, the substrates of *VvCCD1* may be C_27_ apocarotenoids produced through cleavage by enzymatic action (by CCD4 and/or CCD7) or photo-oxidation, and subsequently transported from the chloroplast to the cytosol (Lashbrooke *et al.*, 2013). On the Grape AROS V1.0 array we found oligo sequences corresponding to *VvCCD1*, *VvCCD4a* and *VvCCD4b*. Unlike previous studies that reported a strong increase in their expression (especially *VvCCD4b*) throughout berry development (Mathieu *et al.*, 2005; Guillaumie *et al.*, 2011; Young *et al.*, 2012; Lashbrooke *et al.*, 2013; Cramer *et al.*, 2014), in the present work we only observed a significant up-regulation of *VvCCD4a* at stage 5 compared to stage 1 (Supplementary Table S10), whereas *VvCCD1* and *VvCCD4b* were not differentially expressed during Moscato Bianco berry maturation nor associated to any C_13_-norisoprenoid. This difference might be cultivar-specific, in the sense that the above works investigated varieties where C_13_-norisoprenoids play a greater role on aroma than in Moscato Bianco, e.g. Chardonnay, Sauvignon Blanc and Cabernet Sauvignon.

**2.2 Phenylpropanoid/benzenoid compounds**

Plant phenylpropanoid and benzenoid compounds originate from the aromatic amino acid phenylalanine (PHE), which in turn is formed through seven enzymatic reactions of the shikimate pathway and three of the arogenate pathway. The immediate precursors of the shikimate pathway, phosphoenolpyruvate (PEP) and D-erythrose 4-phosphate (E4P), derive from glycolysis and pentose phosphate pathways, respectively. The phenylpropanoid-related (C_6_-C_2_) compounds, such as 2-phenylethanol, are synthesized directly from PHE (PHE is decarboxylated to phenethylamine by an aromatic amino acid decarboxylase; phenethylamine is converted to 2-phenylacetaldehyde by removal of the amine group, followed by conversion to 2-phenylethanol by phenylacetaldehyde reductase). However, different pathways to convert PHE to phenylacetaldehyde have been also reported (Sun *et al.*, 2016). The benzenoids (C_6_-C_1_), such as benzaldehyde and benzyl alcohol, have *trans*-cinnamic acid (t-CA) as the precursor, which is made from PHE by the activity of phenylalanine ammonia lyase (PAL). The t-CA side chain is shortened through the action of a β-oxidative, a non-β-oxidative pathway or a combination of these pathways. Methyl salicylate, which is present in numerous floral scents, is formed through methylation of salicylic acid, which in turn is synthesized from PHE through the benzenoid branch of the phenylpropanoid pathway or through an alternative pathway via isochorismate (for a review of phenylpropanoid/benzenoid synthesis see Dudareva *et al.*, 2013). The production and emission of these volatiles is spatially and developmentally regulated and in some cases varies remarkably throughout the photoperiod. Such regulation takes place largely at the level of gene expression, which is not limited to the last biochemical step, but is rather shared by one or multiple intermediate steps. Progress in understanding the regulation of phenylpropanoid/benzenoid production has been made especially in petunia, for which a model describing the hierarchical interrelationship between regulatory factors and their downstream targets has been formulated. It includes transcription factors of the R2R3-MYB family that regulate the expression of multiple genes in the shikimate and phenylpropanoid pathways: *EMISSION OF BENZENOIDS II* (*EOBII*), *EMISSION OF BENZENOIDS I* (*EOBI*) and *ODORANT1* (*ODO1*). EOBII transcriptionally activates *EOBI*, and both EOBI and EOBII activate the expression of *ODO1* (Verdonk *et al.*, 2005; Spitzer-Rimon *et al.*, 2010 and 2012).

The biosynthesis of volatile phenylpropanoids/benzenoids has not been studied extensively in grape, although these compounds deserve significant consideration in view of their abundance in hydrolyzates of glycoside isolates from juices and wines. They significantly contribute to wine aroma, for example with the pleasant fruity, floral notes conferred by benzyl alcohol and 2-phenylethanol at low concentrations and the peppermint characteristics of methyl salicylate (Francis and Newton, 2005; Polášková *et al.*, 2008; Robinson *et al.*, 2014). Supplementary Table S10 reports a selection of genes potentially involved in the biosynthesis of the phenylpropanoid/benzenoid compounds investigated in this work, including both structural and regulatory factors. Interestingly, *PhEOBI* shares high homology with *AtMYB24* (Spitzer-Rimon *et al.*, 2012), and for this reason the same grapevine gene prediction (VIT_14s0066g01090) has been reported as a candidate gene in the regulation of both terpenes and phenylpropanoids/benzenoids in the present work (Supplementary Tables S9 and S10).

**2.3 Fatty acid derivatives (C_6_ aliphatic compounds)**

Fatty acid derivatives such as hexanol and *cis*-3-hexenol arise from C_18_ unsaturated fatty acids via the lipoxygenase and alcohol dehydrogenase pathways (for a review see Dudareva *et al.*, 2013). The derived C_6_/C_9_ aldehydes and alcohols are usually synthesized in green organs of plants in response to wounding, but they also provide fruits and vegetables with a characteristic ‘fresh green’ aroma. These compounds confer positive or negative sensory attributes to wine according to their concentration (Francis and Newton, 2005; Dunlevy *et al.*, 2009; Robinson *et al.*, 2014). C_6_ oxylipins are also considered as the precursors of volatile thiols, which define key aromas in several wines (Baumes, 2009). Some members of the *Vitis vinifera* lipoxygenase gene family and two hydroperoxide lyase genes were previously characterized (Podolyan *et al.*, 2010; Zhu *et al.*, 2012). Our findings are in agreement with the results described in those papers and support a role for *VvHPL1* in the formation of C_6_ volatiles (Supplementary Table S10).

**References (not included in the main text)**

Baumes, R. (2009). “Wine aroma precursors”, in *Wine Chemistry and Biochemistry*, eds. M. V. Moreno-Arribas, M. C. Polo, 251-274. doi: 10.1007/978-0-387-74118-5_11

Baumes, R., Wirth, J., Bureau, S., Gunata, Y., and Razungles, A. (2002). Biogeneration of C_13_-norisoprenoid compounds: experiments supportive for an apo-carotenoid pathway in grapevines. *Anal. Chim. Acta* 458, 3-14. doi: 10.1016/S0003-2670(01)01589-6

Chen, F., D'Auria, J. C., Tholl, D., Ross, J. R., Gershenzon, J., Noel, J. P., et al. (2003). An *Arabidopsis thaliana* gene for methylsalicylate biosynthesis, identified by a biochemical genomics approach, has a role in defense. *Plant J.* 36, 577-588. doi: 10.1046/j.1365-313X.2003.01902.x

Effmert, U., Saschenbrecker, S., Ross, J., Negre, F., Fraser, C. M., Noel, J. P. et al. (2005). Floral benzenoid carboxyl methyltransferases: from in vitro to in planta function. *Phytochemistry* 66, 1211-1230. doi: 10.1016/j.phytochem.2005.03.031

Francis, I. L., and Newton, J. L. (2005). Determining wine aroma from compositional data. *Aust. J. Grape Wine R.* 11, 114-126. doi: 10.1111/j.1755-0238.2005.tb00283.x

Gonda, I., Bar, E., Portnoy, V., Lev, S., Burger, J., Schaffer, A. A., et al. (2010). Branched-chain and aromatic amino acid catabolism into aroma volatiles in *Cucumis melo* L. fruit. *J Exp. Bot.* 61, 1111-1123. doi: 10.1093/jxb/erp390

Guterman, I., Shalit, M., Menda, N., Piestun, D., Yelin, M. D., Shalev, G., et al. (2002). Rose scent: genomics approach to discovering novel floral fragrance-related genes. *Plant Cell* 14, 2325-2338. doi: 10.1105/tpc.005207

Guillaumie, S., Fouquet, R., Kappel, C., Camps, C., Terrier, N., Moncomble, D., et al. (2011). Transcriptional analysis of late ripening stages of grapevine berry. *BMC Plant Biol.* 11, 165. doi: 10.1186/1471-2229-11-165

Lashbrooke, J. G., Young, P. R., Dockrall, S. J., Vasanth, K., and Vivier, M. A. (2013). Functional characterisation of three members of the *Vitis vinifera* L. carotenoid cleavage dioxygenase gene family. *BMC Plant Biol.* 13, 156. doi: 10.1186/1471-2229-13-156

Mathieu, S., Terrier, N., Procureur, J., Bigey, F., and Günata, Z. (2005). Carotenoid cleavage dioxygenase from *Vitis vinifera* L.: functional characterization and expression during grape berry development in relation to C_13_-norisoprenoid accumulation. *J. Exp. Bot.* 56, 2721-2731. doi: 10.1093/jxb/eri265

Mendes-Pinto, M. M. (2009). Carotenoid breakdown products the-norisoprenoids-in wine aroma. *Arch. Biochem. Biophys.* 483, 236-245. doi: 10.1016/j.abb.2009.01.008

Podolyan, A., White, J., Jordan, B., and Winefield, C. (2010). Identification of the lipoxygenase gene family from *Vitis vinifera* and biochemical characterisation of two 13-lipoxygenases expressed in grape berries of Sauvignon Blanc. *Funct. Plant Biol.* 37, 767-784. doi: 10.1071/FP09271

Polášková, P., Herszage, J., Ebeler, S. E. (2008). Wine flavor: chemistry in a glass. *Chem. Soc. Rev.* 37, 2478-2489. doi: 10.1039/b714455p

Qian, X., Xu, X. Q., Yu, K. J., Zhu, B. Q., Lan, Y. B., Duan, C. Q., et al. (2016). Varietal dependence of GLVs accumulation and LOX-HPL pathway gene expression in four *Vitis vinifera* wine grapes. *Int. J. Mol. Sci.* 17, 1924. doi:10.3390/ijms17111924

Ross, J. R., Nam, K. H., D'Auria, J. C., and Pichersky, E. (1999). S-Adenosyl-L-methionine:salicylic acid carboxyl methyltransferase, an enzyme involved in floral scent production and plant defense, represents a new class of plant methyltransferases. *Arch. Biochem. Biophys.* 367, 9-16. doi: 10.1006/abbi.1999.1255

Sablowski, R. W. M., Baulcombe, D. C., and Bevan, M. (1995). Expression of a flower-specific Myb protein in leaf cells using a viral vector causes ectopic activation of a target promoter. *Proc. Natl. Acad. Sci. USA* 92, 6901-6905. doi: 10.1073/pnas.92.15.6901

Spitzer-Rimon, B., Farhi, M., Albo, B., Cna’ani, A., Ben Zvi, M. M., Masci, T. et al. (2012). The R2R3-MYB–like regulatory factor EOBI, acting downstream of EOBII, regulates scent production by activating *ODO1* and structural scent-related genes in Petunia. *Plant Cell* 24, 5089-5105. doi: 10.1105/tpc.112.105247

Spitzer-Rimon, B., Marhevka, E., Barkai, O., Marton, I., Edelbaum, O., Masci, T. et al. (2010). *EOBII*, a gene encoding a flower-specific regulator of phenylpropanoid volatiles’ biosynthesis in petunia. *Plant Cell* 22, 1961-1976. doi: 10.1105/tpc.109.067280

Verdonk, J. C., Haring, M. A., van Tunen, A. J., and Schuurink, R. C. (2005). *ODORANT1* regulates fragrance biosynthesis in petunia flowers. *Plant Cell* 17, 1612-1624. doi: 10.1105/tpc.104.028837

Wang, S., Liu, J., Feng, Y., Niu, X., Giovannoni, J., and Liu, Y. (2008). Altered plastid levels and potential for improved fruit nutrient content by downregulation of the tomato DDB1-interacting protein CUL4. *Plant J.* 55, 89-103. doi: 10.1111/j.1365-313X.2008.03489.x

Winterhalter, P., and Rouseff, R. (2002). “Carotenoid-derived aroma compounds: an introduction”, in *Carotenoid derived aroma compounds*, eds. P. Winterhalter, R. Rouseff, 1-19. doi: 10.1021/bk-2002-0802.ch001

Young, P. R., Lashbrooke, J. G., Alexandersson, E., Jacobson, D., Moser, C., Velasco, R., et al. (2012). The genes and enzymes of the carotenoid metabolic pathway in *Vitis vinifera* L. *BMC Genomics* 13, 243. doi: 10.1186/1471-2164-13-243

Zhu, B. Q., Xu, X. Q., Wu, Y. W., Duan, C. Q., and Pan, Q. H. (2012). Isolation and characterization of two hydroperoxide lyase genes from grape berries. *Mol. Biol. Rep.* 39, 7443-7455. doi: 10.1007/s11033-012-1577-0
