# Supplementary material for: Drawing Links from Transcriptome to Metabolites: The Evolution of Aroma in the Ripening Berry of Moscato Bianco (Vitis vinifera L.)
Source: Front Plant Sci. 2017 May 16;8:780. doi: 10.3389/fpls.2017.00780 (PMC5432621; doi:10.3389/fpls.2017.00780)
Supplement: Supplementary file 12 [file Image1.pdf]

A

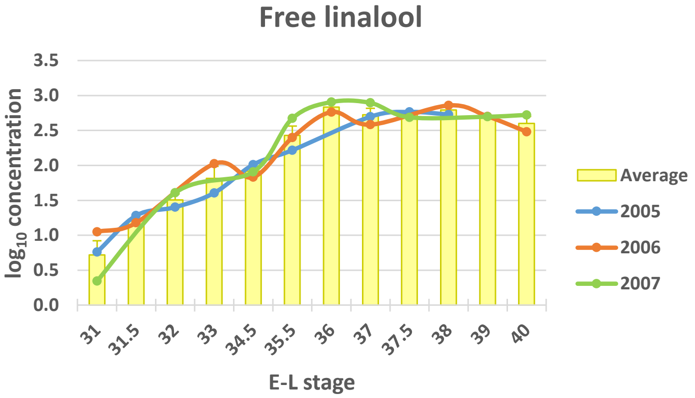

2005-2006	2005-2007	2006-2007
0.95**	0.96**	0.98**

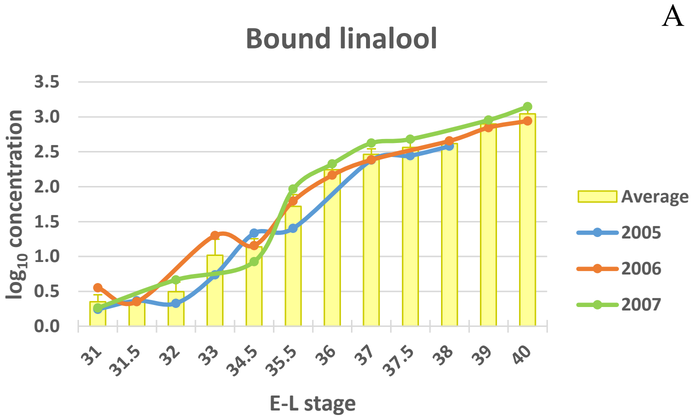

2005-2006	2005-2007	2006-2007
0.96**	0.95**	0.99**

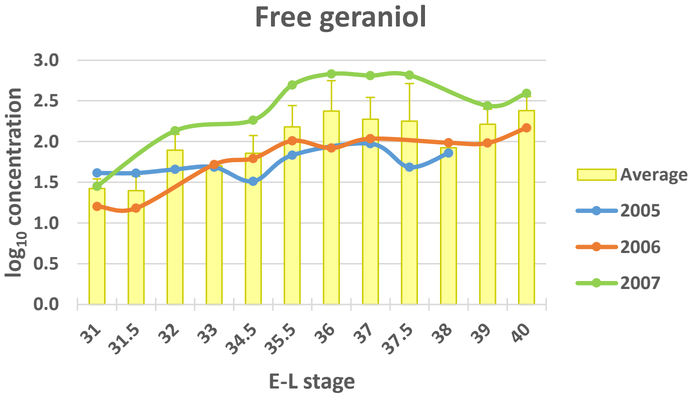

2005-2006	2005-2007	2006-2007
NS	NS	0.91**

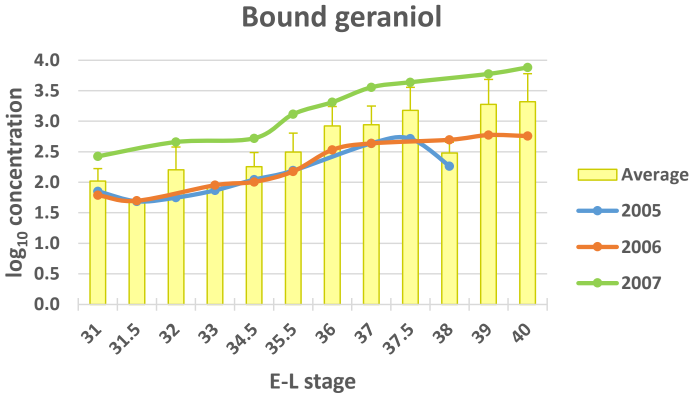

2005-2006	2005-2007	2006-2007
0.91**	0.96**	0.98**

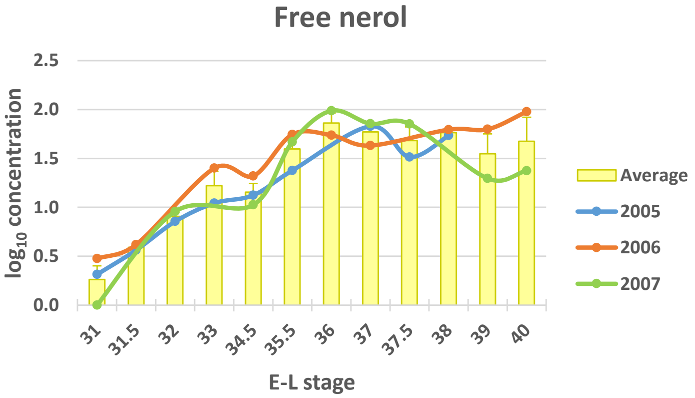

2005-2006	2005-2007	2006-2007
0.94**	0.97**	0.86*

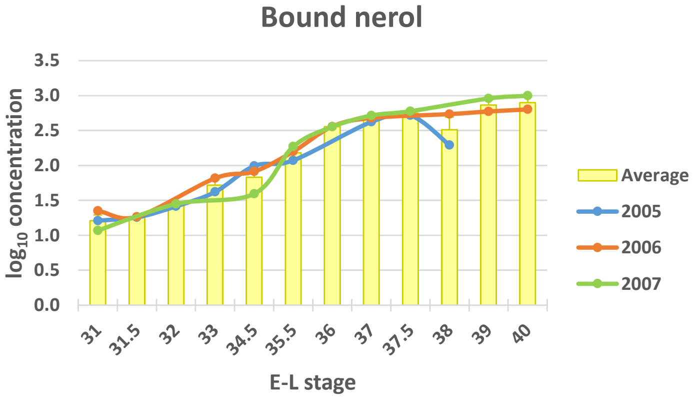

2005-2006	2005-2007	2006-2007
0.96**	0.96**	0.99**

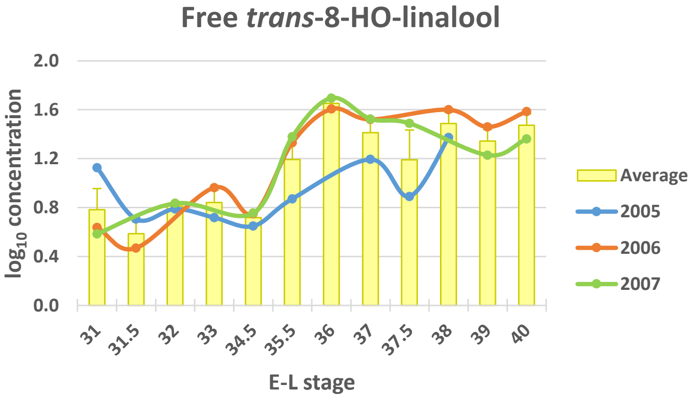

2005-2006	2005-2007	2006-2007
NS	NS	0.95**

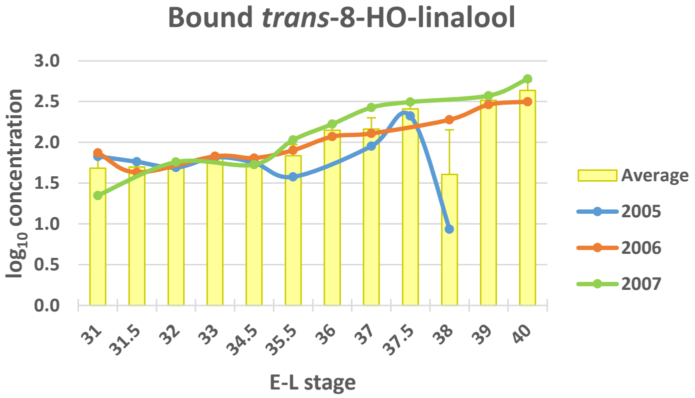

2005-2006	2005-2007	2006-2007
NS	NS	0.88**

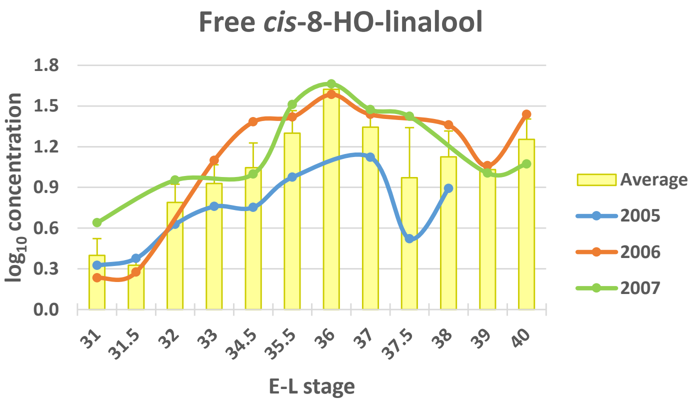

2005-2006	2005-2007	2006-2007
0.94**	NS	0.80*

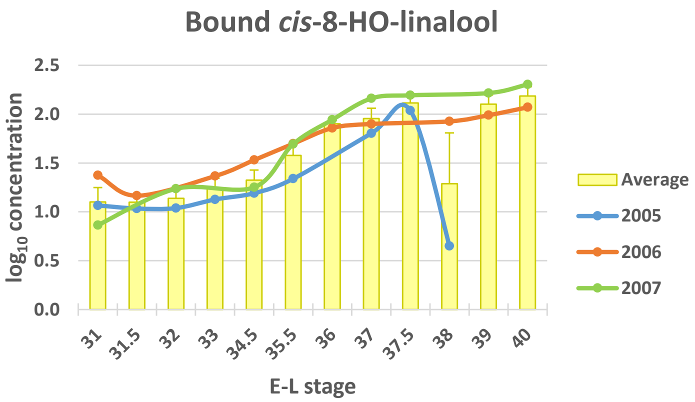

2005-2006	2005-2007	2006-2007
NS	0.94**	0.99**

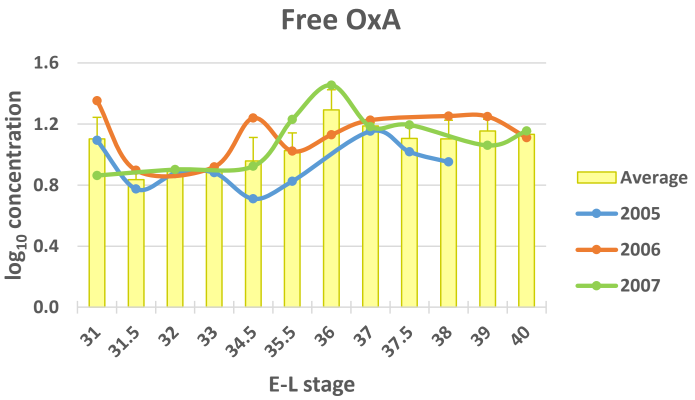

2005-2006	2005-2007	2006-2007
NS	NS	NS

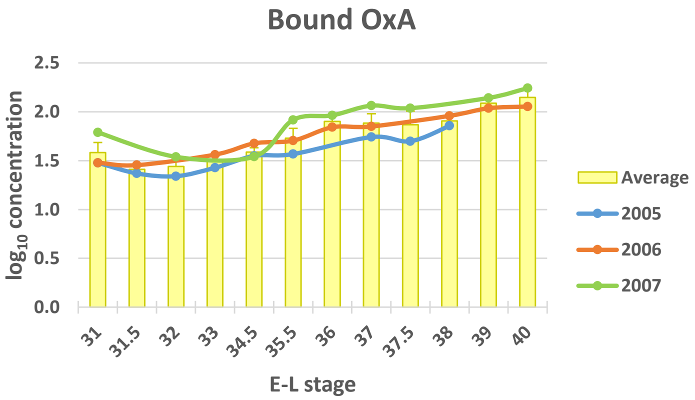

2005-2006	2005-2007	2006-2007
0.97**	0.83*	0.80*

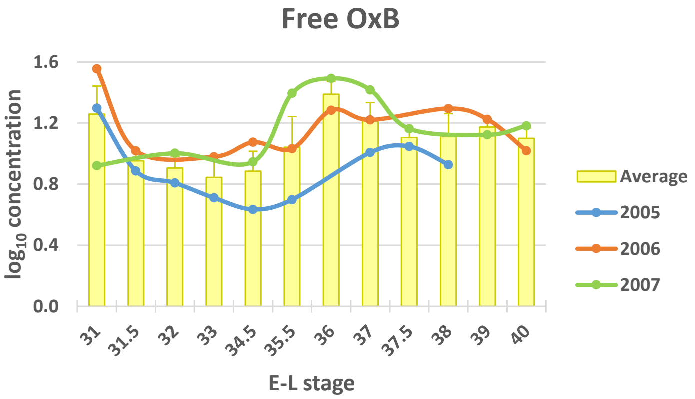

2005-2006	2005-2007	2006-2007
0.89**	NS	NS

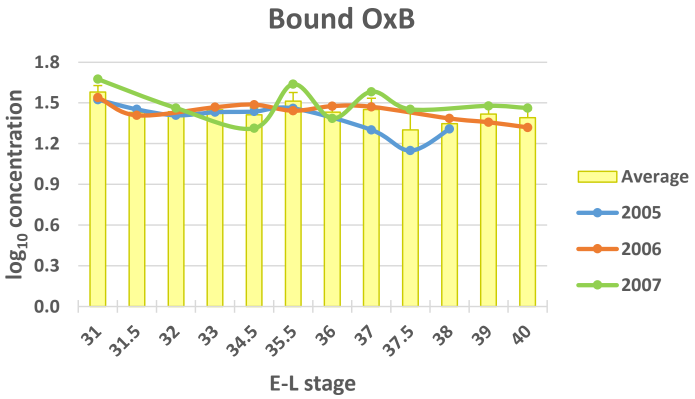

2005-2006	2005-2007	2006-2007
NS	NS	NS

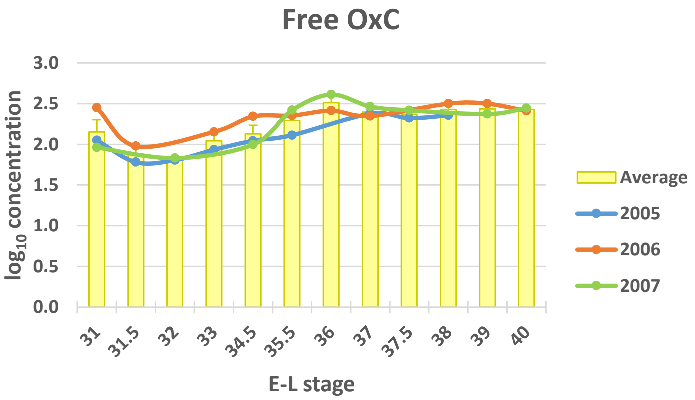

2005-2006	2005-2007	2006-2007
0.79*	0.88*	NS

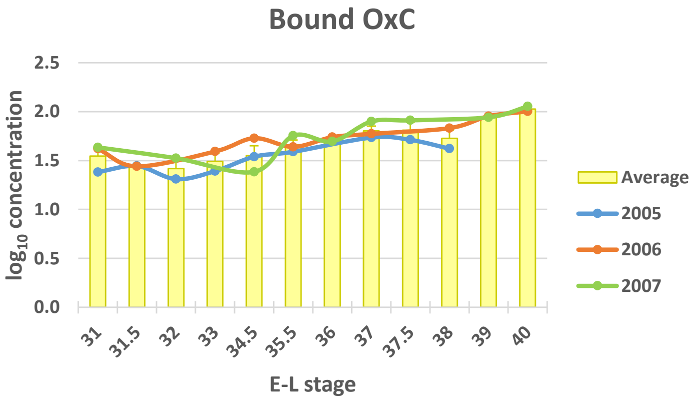

2005-2006	2005-2007	2006-2007
NS	NS	NS

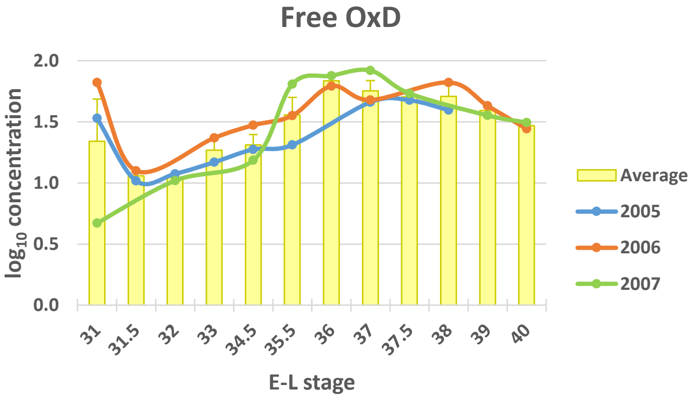

2005-2006	2005-2007	2006-2007
0.93**	NS	NS

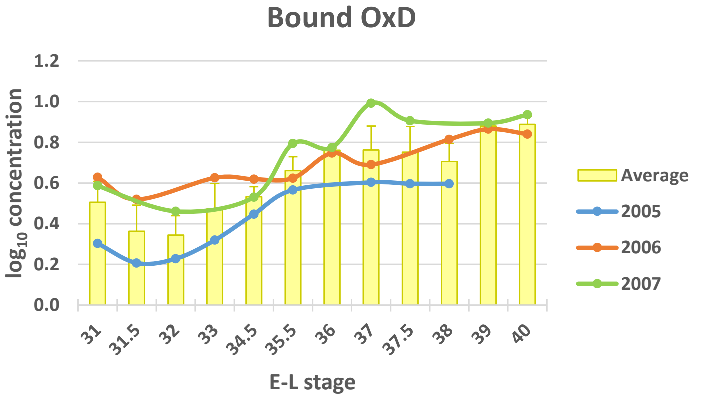

2005-2006	2005-2007	2006-2007
NS	0.89*	NS

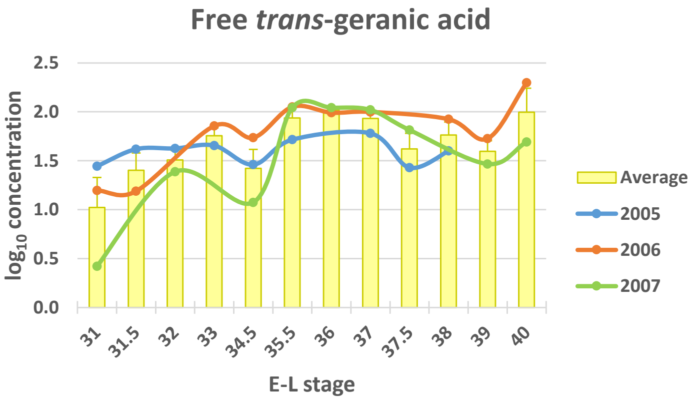

2005-2006	2005-2007	2006-2007
NS	NS	0.86*

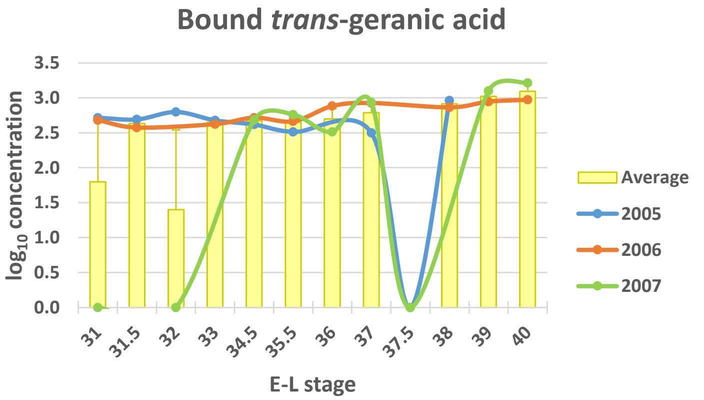

2005-2006	2005-2007	2006-2007
NS	*NS*	*NS*

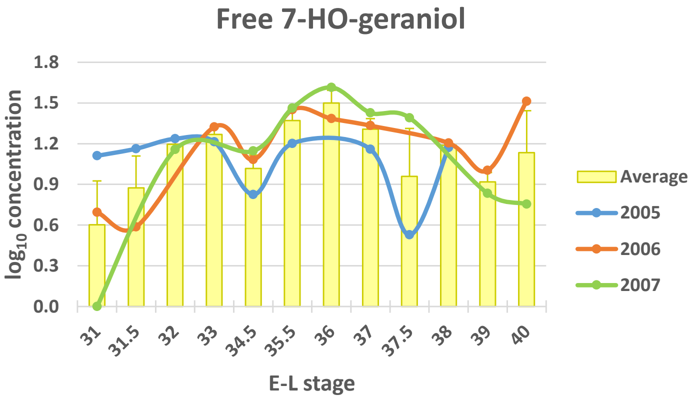

2005-2006	2005-2007	2006-2007
NS	NS	NS

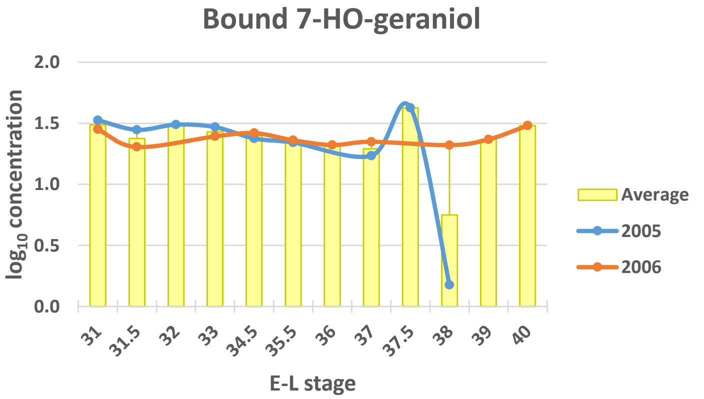

2005-2006	2005-2007	2006-2007
NS	NA	NA

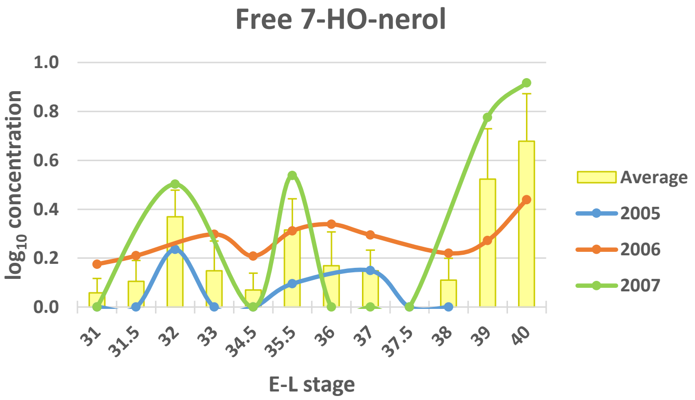

2005-2006	2005-2007	2006-2007
NS	NS	NS

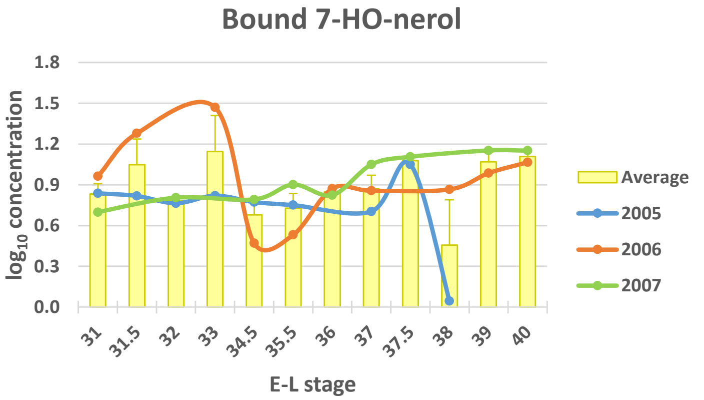

2005-2006	2005-2007	2006-2007
NS	NS	NS

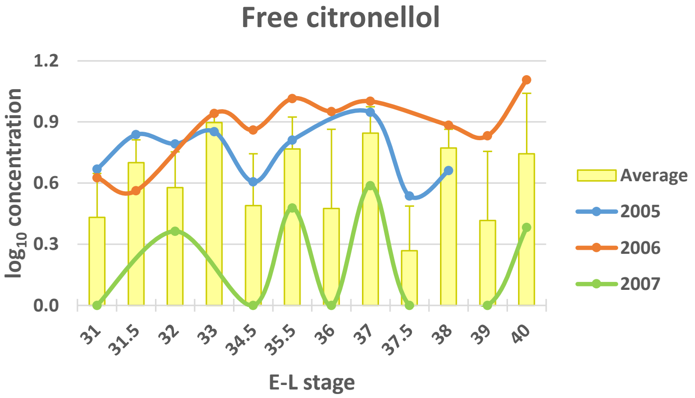

2005-2006	2005-2007	2006-2007
NS	0.95**	NS

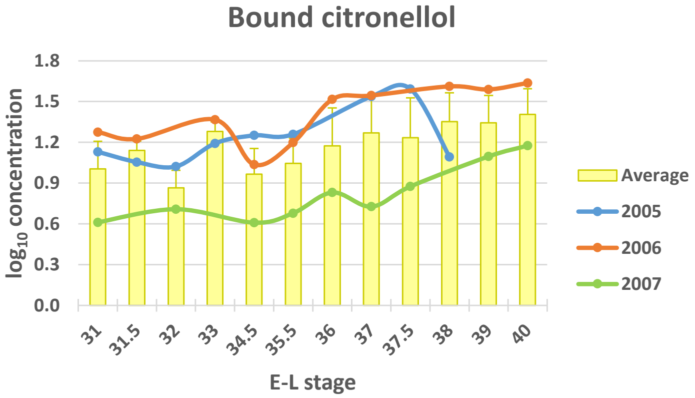

2005-2006	2005-2007	2006-2007
NS	NS	0.81*

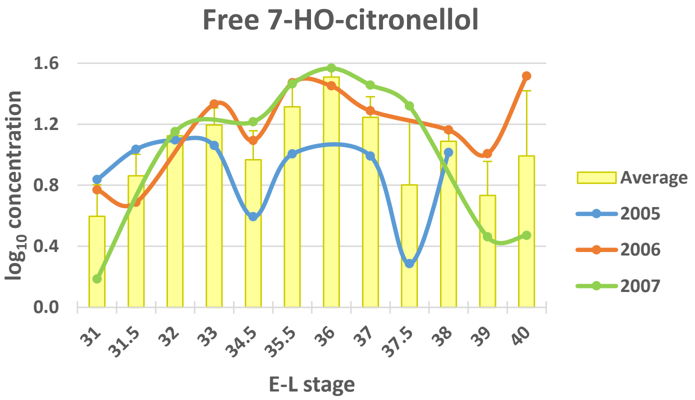

2005-2006	2005-2007	2006-2007
NS	NS	NS

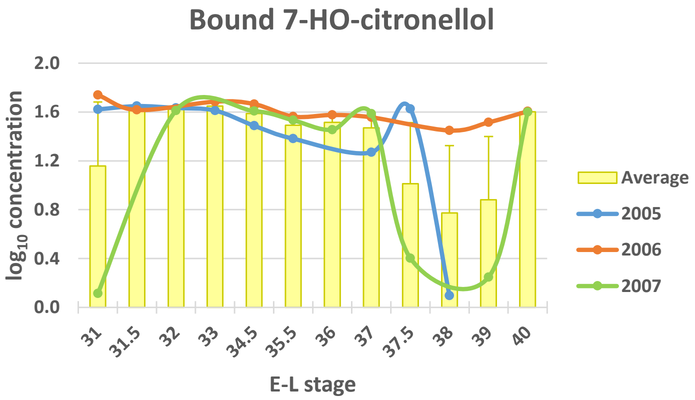

2005-2006	2005-2007	2006-2007
*0.84**	NS	NS

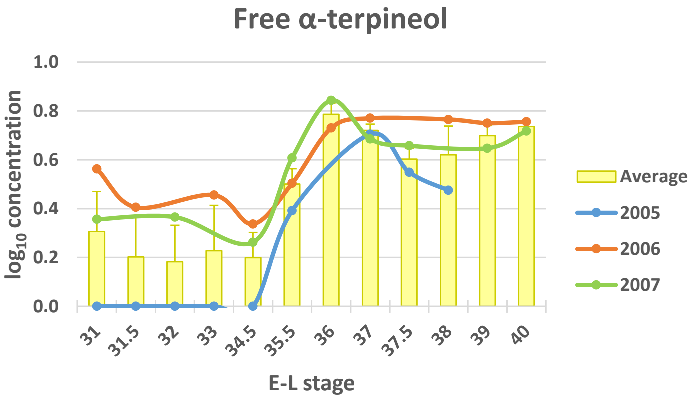

2005-2006	2005-2007	2006-2007
0.84*	0.96**	0.84*

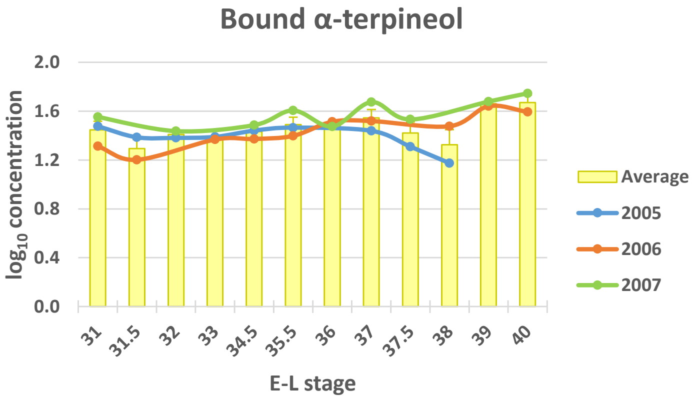

2005-2006	2005-2007	2006-2007
NS	NS	NS

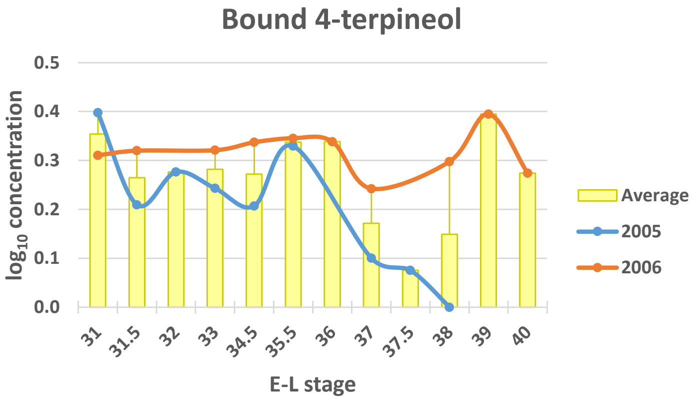

2005-2006	2005-2007	2006-2007
NS	NA	NA

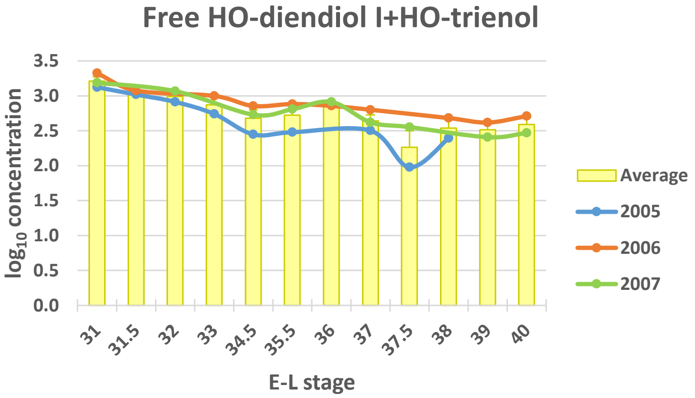

2005-2006	2005-2007	2006-2007
0.94**	0.94**	0.94**

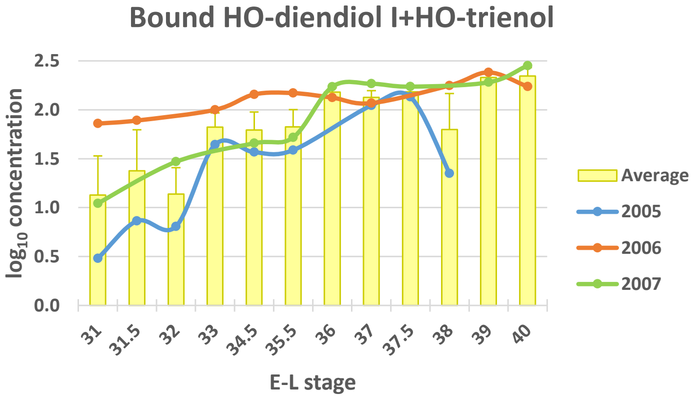

2005-2006	2005-2007	2006-2007
NS	0.96**	NS

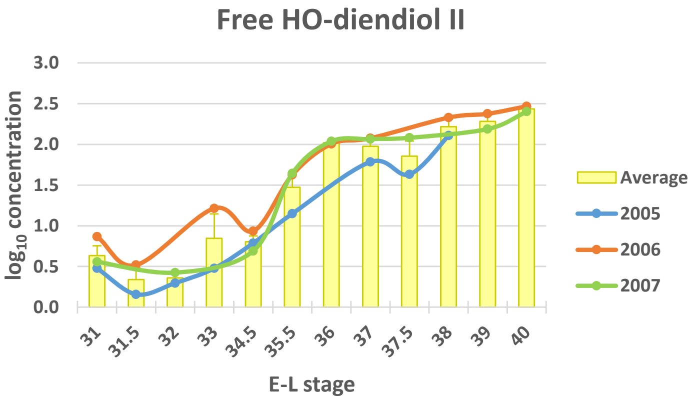

2005-2006	2005-2007	2006-2007
0.97**	0.97**	0.99**

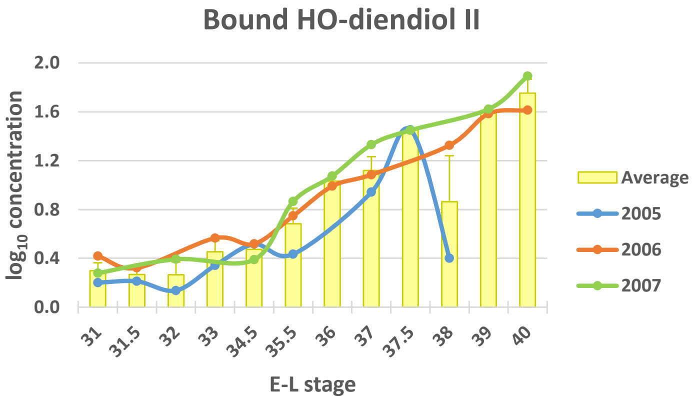

2005-2006	2005-2007	2006-2007
NS	0.90*	0.98**

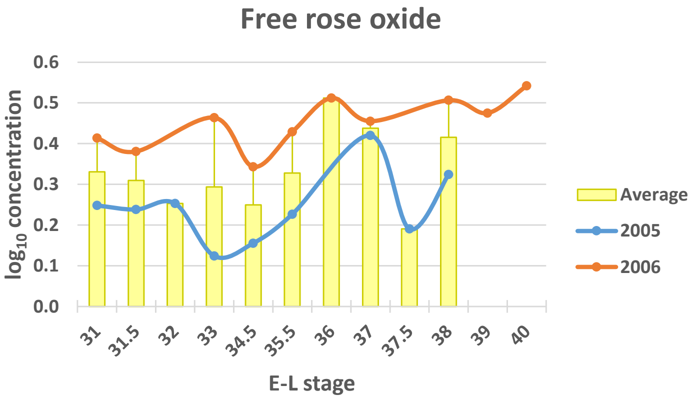

2005-2006	2005-2007	2006-2007
NS	NA	NA

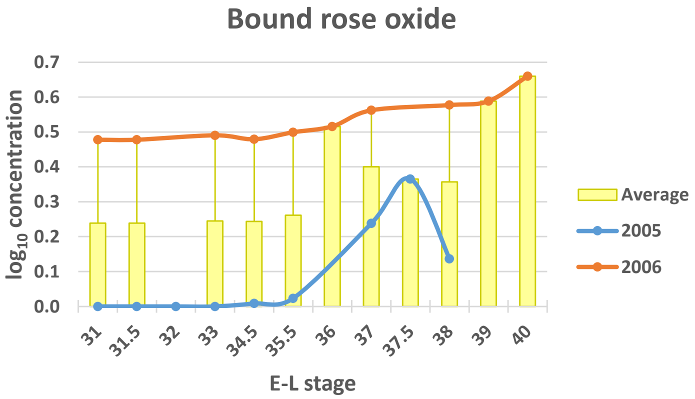

2005-2006	2005-2007	2006-2007
*0.89***	NA	NA

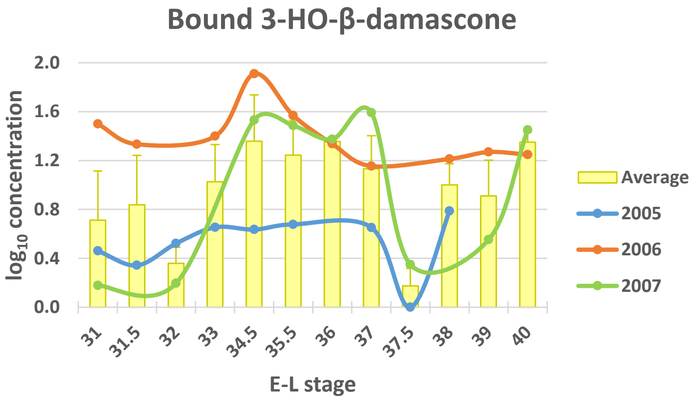

2005-2006	2005-2007	2006-2007
NS	NS	NS

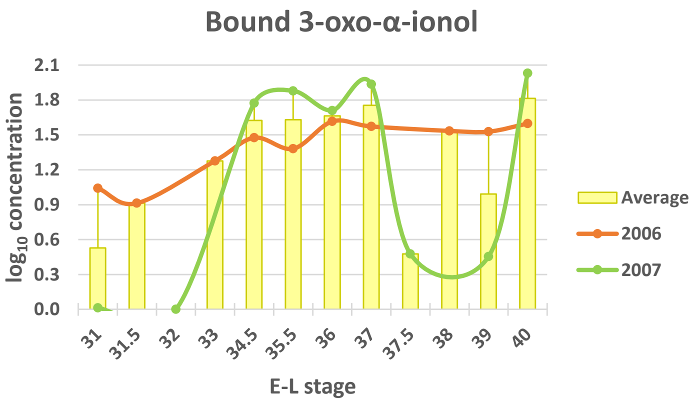

2005-2006	2005-2007	2006-2007
NA	NA	NS

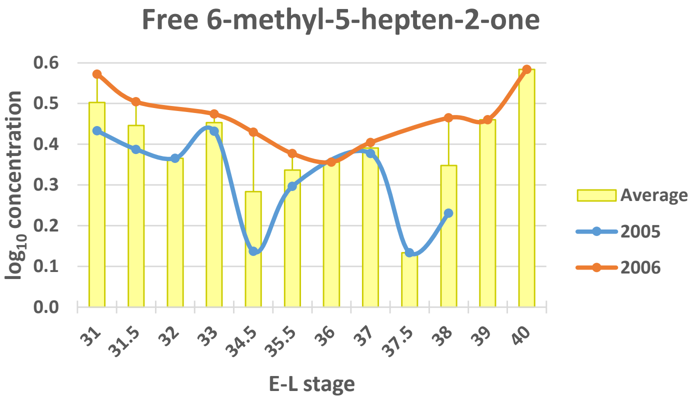

2005-2006	2005-2007	2006-2007
NS	NA	NA

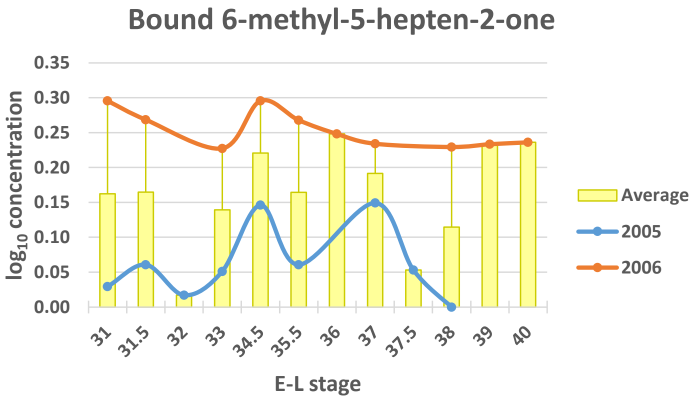

2005-2006	2005-2007	2006-2007
NS	NA	NA

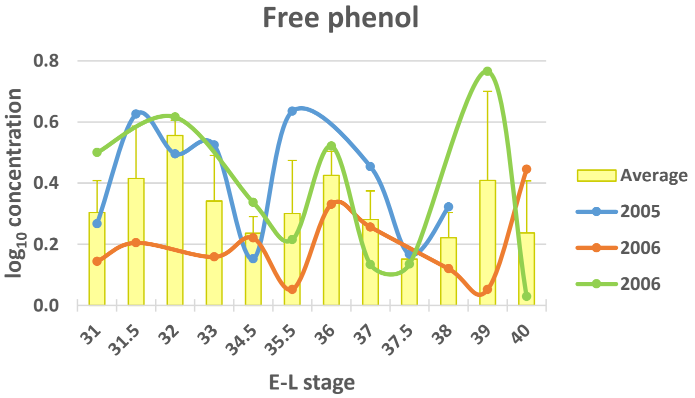

2005-2006	2005-2007	2006-2007
NS	NS	NS

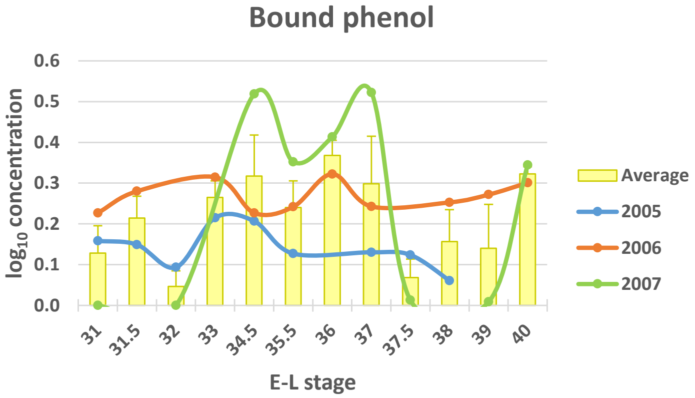

2005-2006	2005-2007	2006-2007
NS	NS	NS

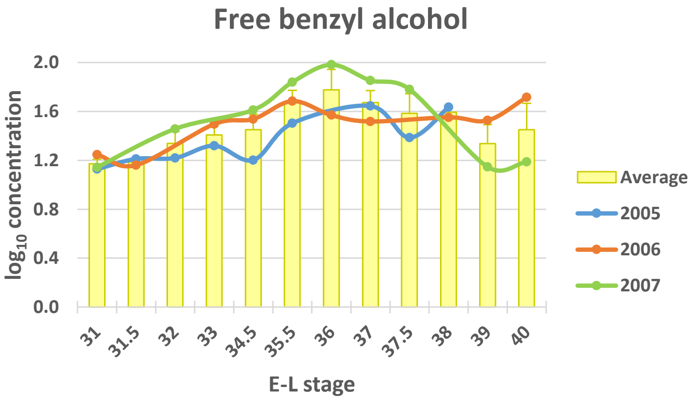

2005-2006	2005-2007	2006-2007
NS	0.85*	NS

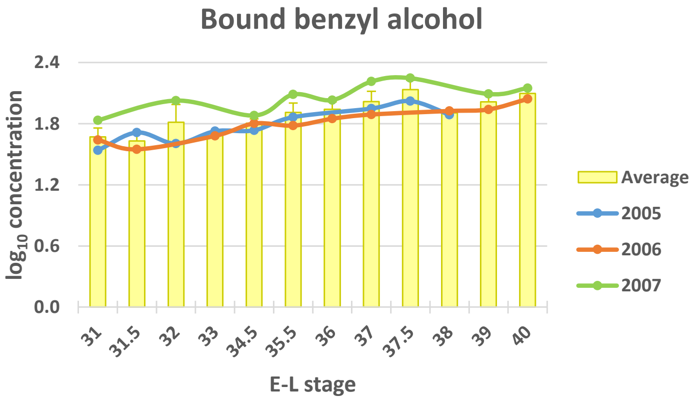

2005-2006	2005-2007	2006-2007
0.77*	0.87*	0.76*

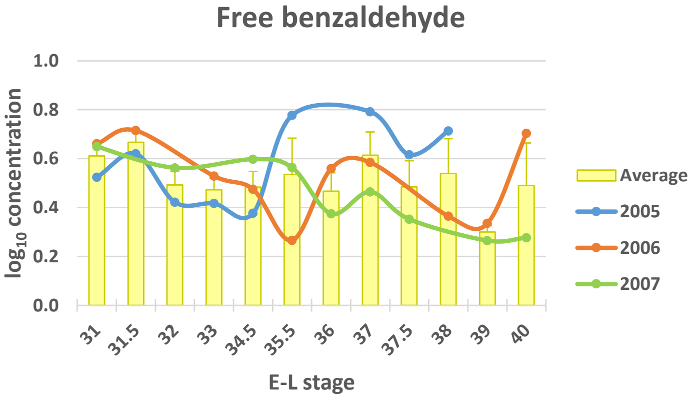

2005-2006	2005-2007	2006-2007
NS	NS	NS

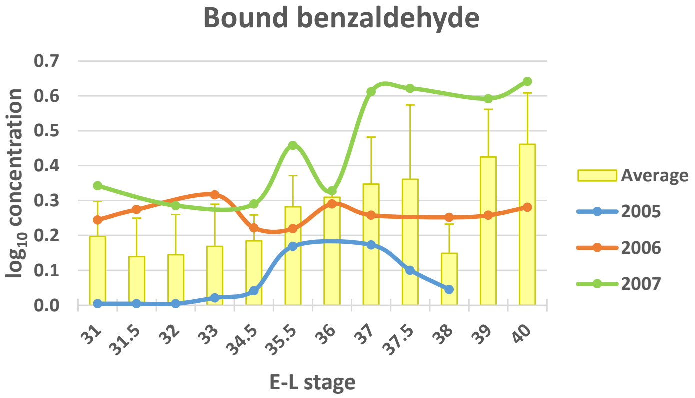

2005-2006	2005-2007	2006-2007
NS	NS	NS

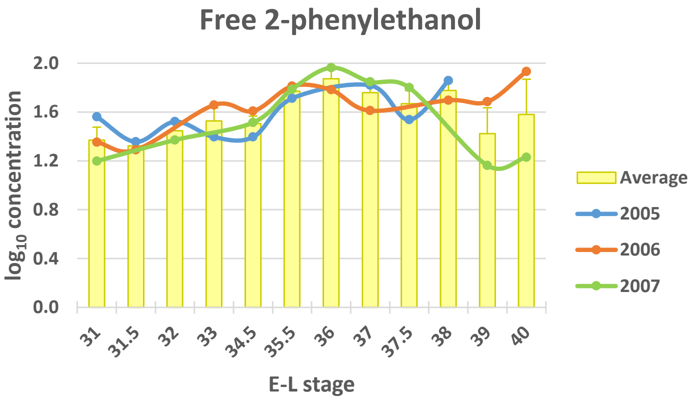

2005-2006	2005-2007	2006-2007
NS	NS	NS

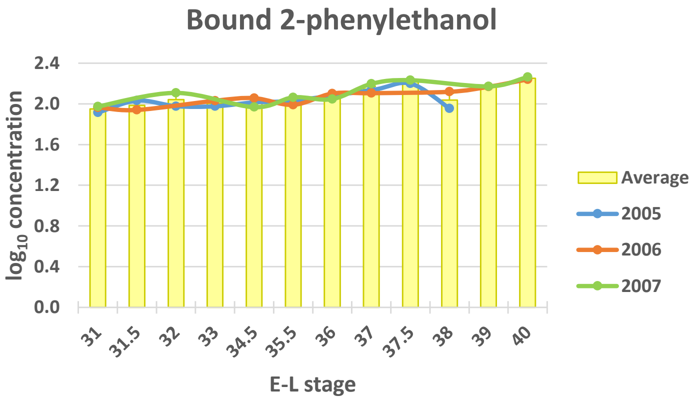

2005-2006	2005-2007	2006-2007
NS	0.87*	0.84*

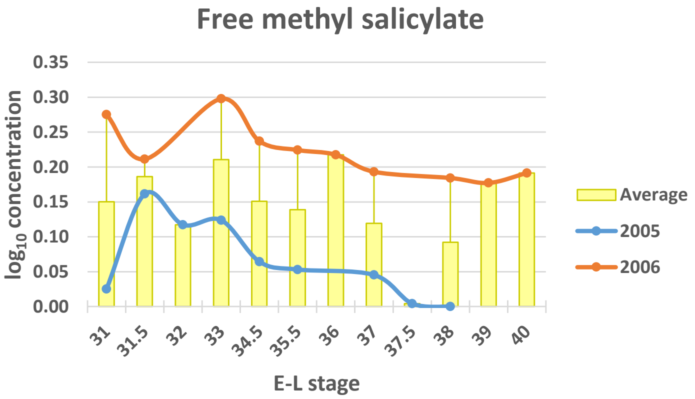

2005-2006	2005-2007	2006-2007
NS	NA	NA

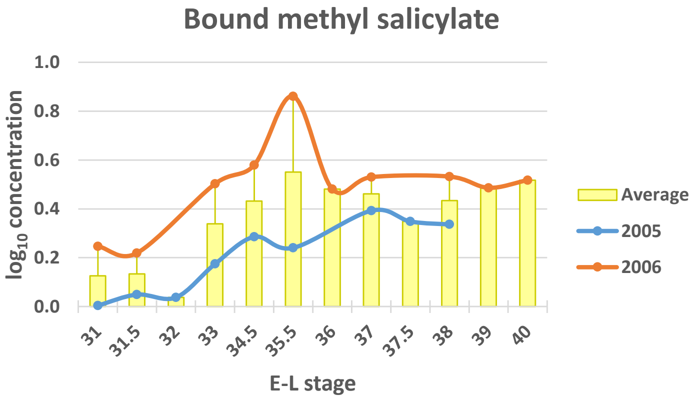

2005-2006	2005-2007	2006-2007
NS	NA	NA

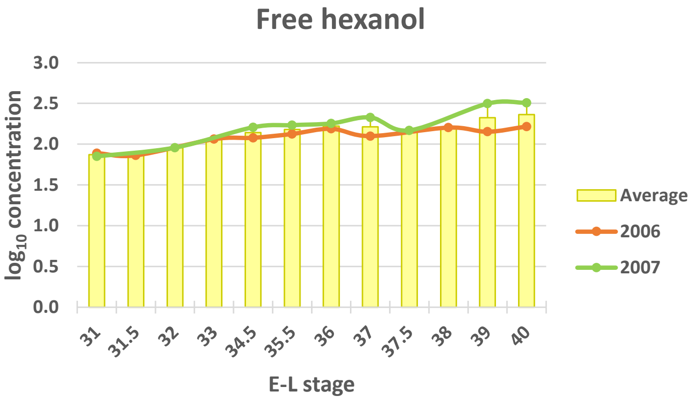

2005-2006	2005-2007	2006-2007
NA	NA	0.88**

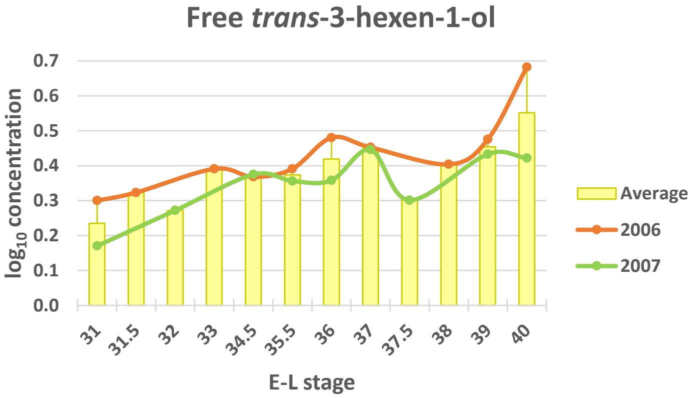

2005-2006	2005-2007	2006-2007
NA	NA	NS

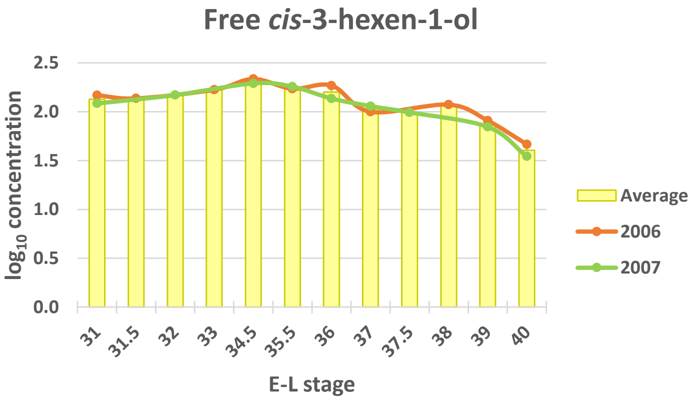

2005-2006	2005-2007	2006-2007
NA	NA	0.96**

B

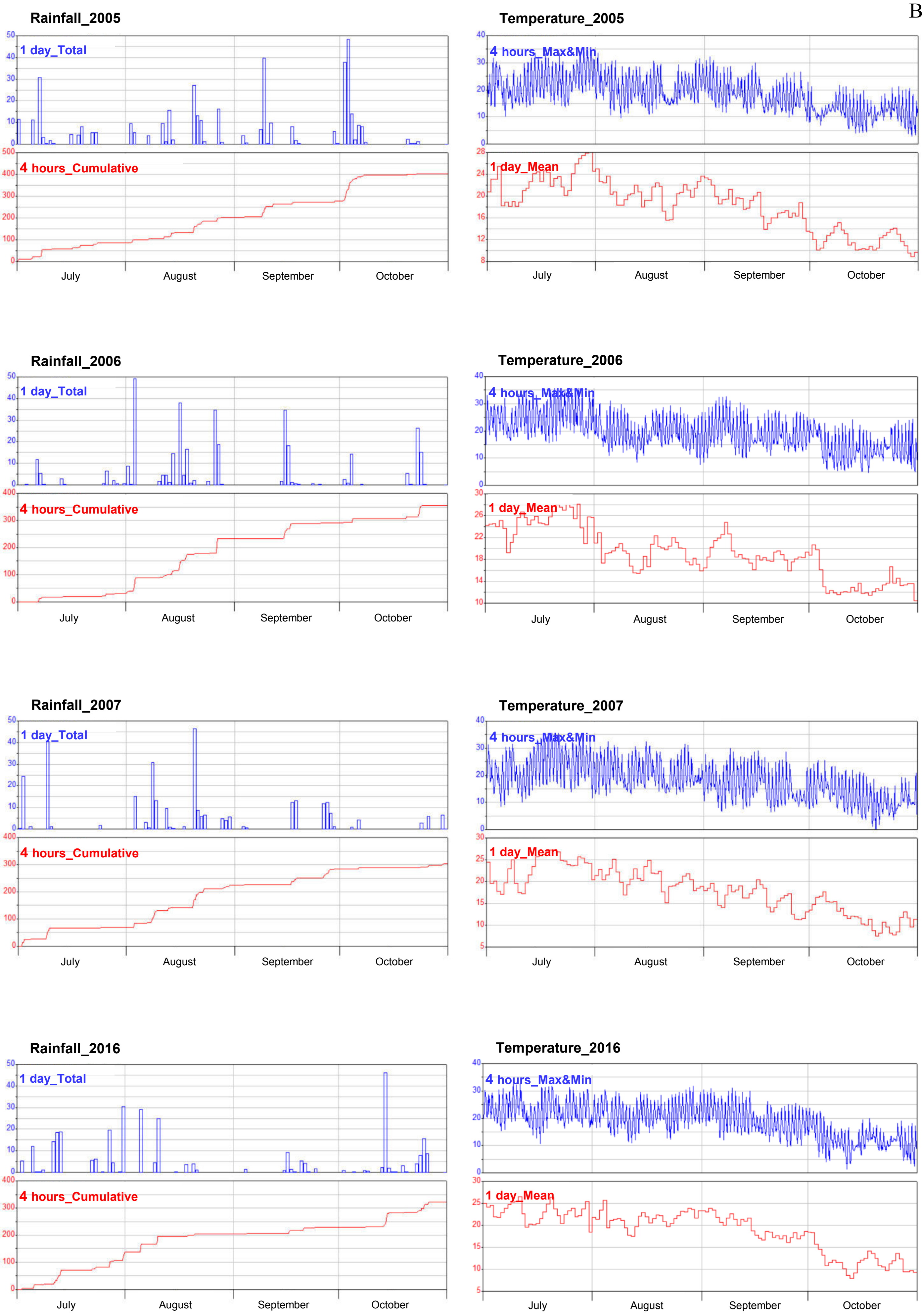

Supplementary Figure S1: A) Aromatic compound content in the Moscato Bianco ripening berry over 3 seasons. The colored lines correspond to the log10-transformed concentration of free and glycosidically bound metabolites in 2005, 2006 and 2007. The yellow columns with bars represent the average ± standard error of the 3 years considered as biological replicates. At the bottom of each figure, Pearson correlations between seasonal quantifications are marked with one or two stars when significant at the 0.05 and 0.01 level, respectively. Abbreviation: E-L stage = growth stage according to the modified Eichhorn-Lorenz scheme (Coombe, 1995). The decimal E-L stages were arbitrarily assigned by the authors of the present study to facilitate the alignment of the sampling dates from the three different years. B) Climatic conditions (rainfall and temperature) from July to October in the 4 seasons under investigation. Historical data were retrieved from Meteotrentino archive (http://www.meteotrentino.it/).
