# Supplementary material for: Drawing Links from Transcriptome to Metabolites: The Evolution of Aroma in the Ripening Berry of Moscato Bianco (Vitis vinifera L.)
Source: Front Plant Sci. 2017 May 16;8:780. doi: 10.3389/fpls.2017.00780 (PMC5432621; doi:10.3389/fpls.2017.00780)
Supplement: Supplementary file 13 [file Image2.pdf]

C_{13}-norisoprenoids

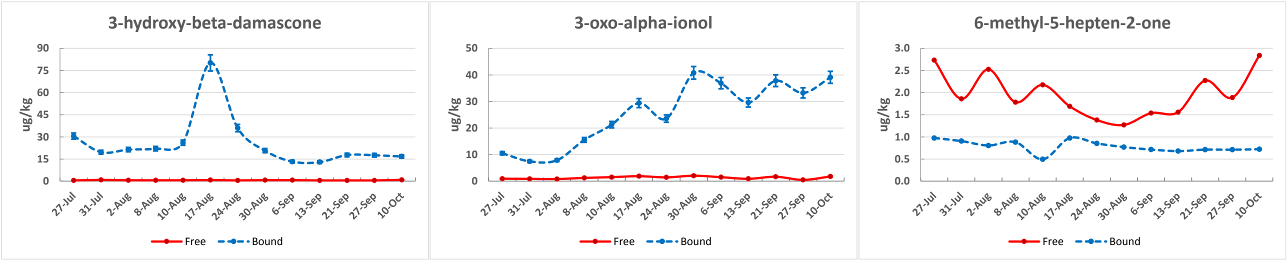

Phenylpropanoids/benzenoids

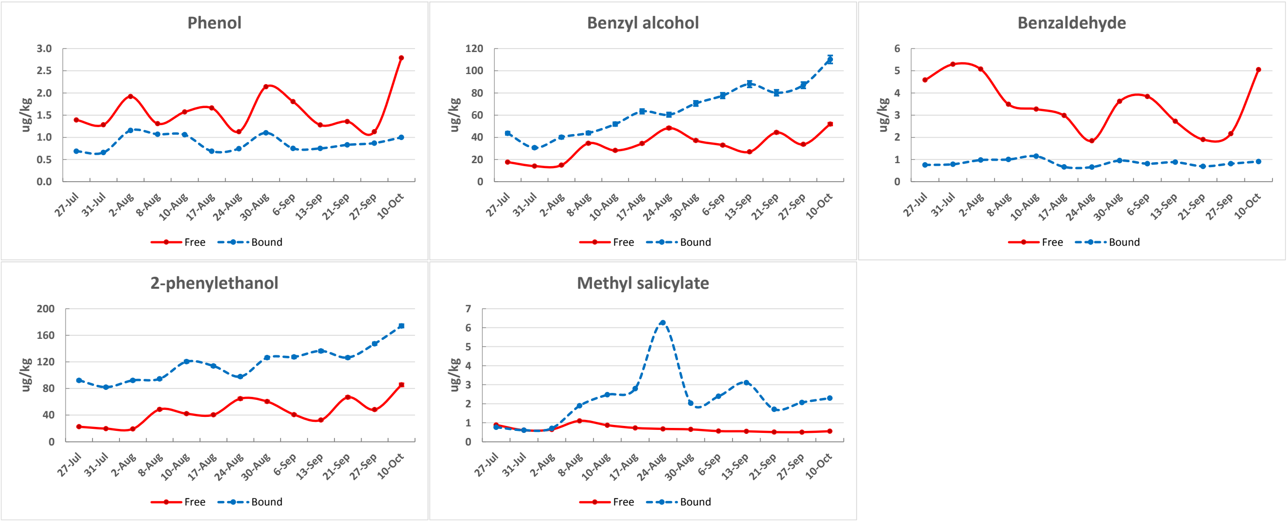

C_6 aliphatic alcohols

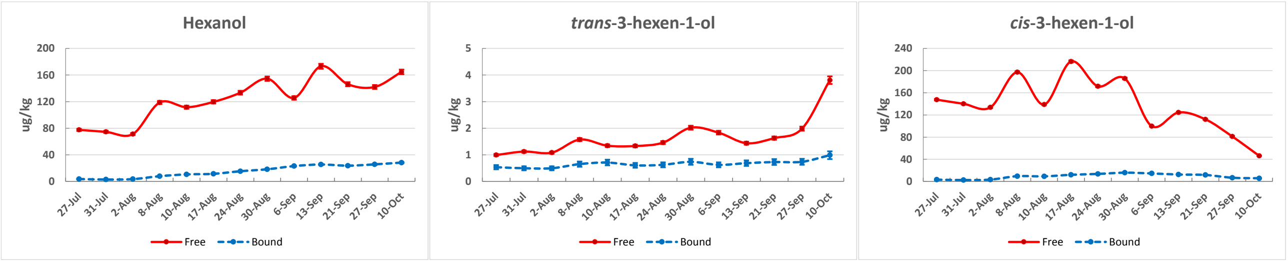

Supplementary Figure S2: Evolution of C_{13}-norisoprenoids, phenylpropanoids/benzenoids and C_6 aliphatic alcohols in their free (solid red line) and glycosidically bound (dashed blue line) form during Moscato Bianco berry ripening in 2006. A single biological replicate was considered at each stage; bars correspond to the standard error calculated from six technical replicates, as described in Supplementary Method S1 (technical replication is not available for 6-methyl-5-hepten-2-one, phenol, benzaldehyde and methyl salicylate). The metabolites were quantified by using solid SPE-HRGC-MS and referring to the internal standard 1-heptanol. The lines connecting data points were smoothed through the specific option provided by Excel.
