# Supplementary material for: Drawing Links from Transcriptome to Metabolites: The Evolution of Aroma in the Ripening Berry of Moscato Bianco (Vitis vinifera L.)
Source: Front Plant Sci. 2017 May 16;8:780. doi: 10.3389/fpls.2017.00780 (PMC5432621; doi:10.3389/fpls.2017.00780)
Supplement: Supplementary file 14 [file Image3.pdf]

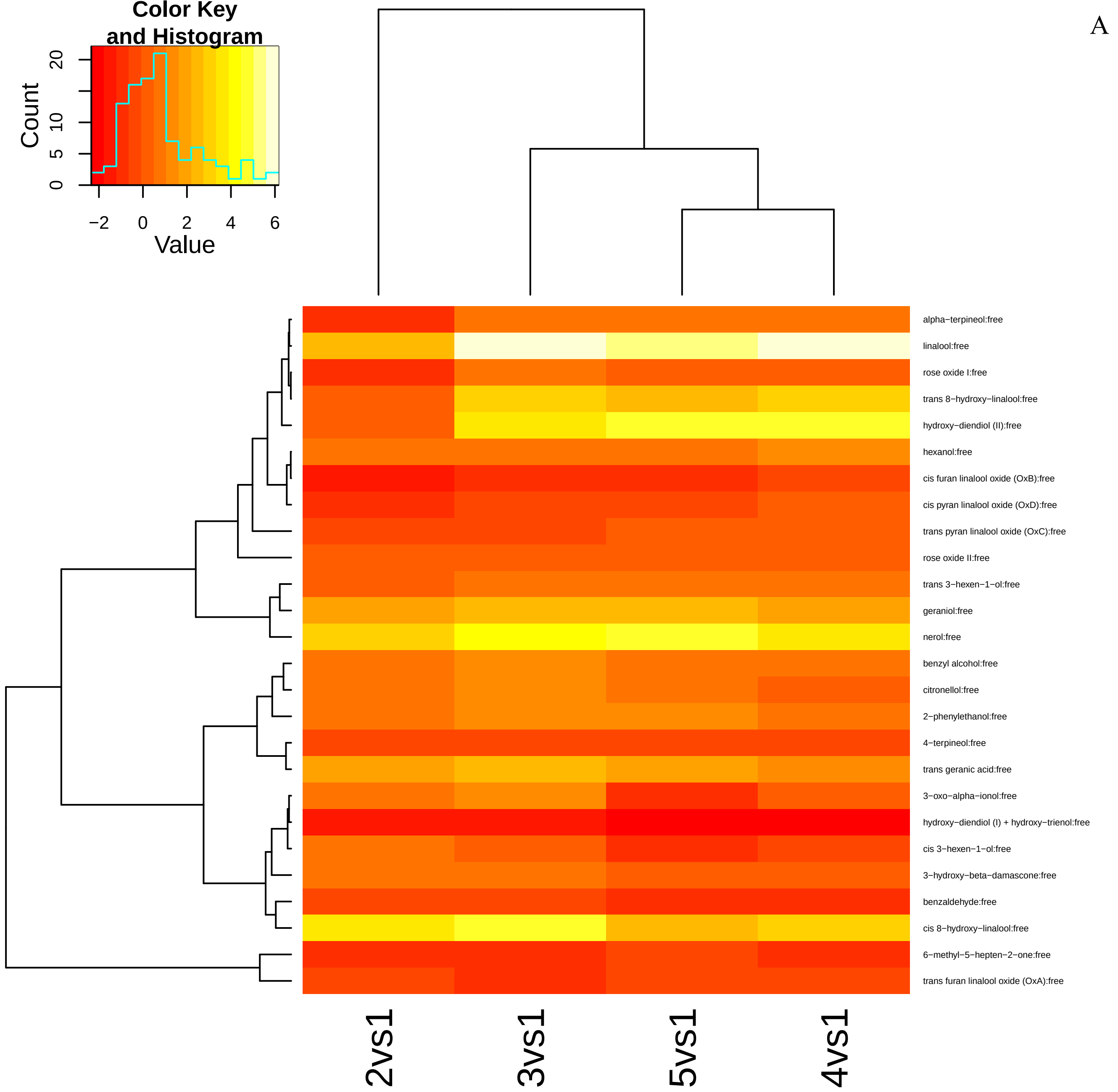
A
Color Key
and Histogram
Count
Value
−2
0
2
4
6
0
5
10
20
alpha−terpineol:free
linalool:free
rose oxide I:free
trans 8−hydroxy−linalool:free
hydroxy−diendiol (II):free
hexanol:free
cis furan linalool oxide (OxB):free
cis pyran linalool oxide (OxD):free
trans pyran linalool oxide (OxC):free
rose oxide II:free
trans 3−hexen−1−ol:free
geraniol:free
nerol:free
benzyl alcohol:free
citronellol:free
2−phenylethanol:free
4−terpineol:free
trans geranic acid:free
3−oxo−alpha−ionol:free
hydroxy−diendiol (I) + hydroxy−trienol:free
cis 3−hexen−1−ol:free
3−hydroxy−beta−damascone:free
benzaldehyde:free
cis 8−hydroxy−linalool:free
6−methyl−5−hepten−2−one:free
trans furan linalool oxide (OxA):free
2vs1
3vs1
5vs1
4vs1

B

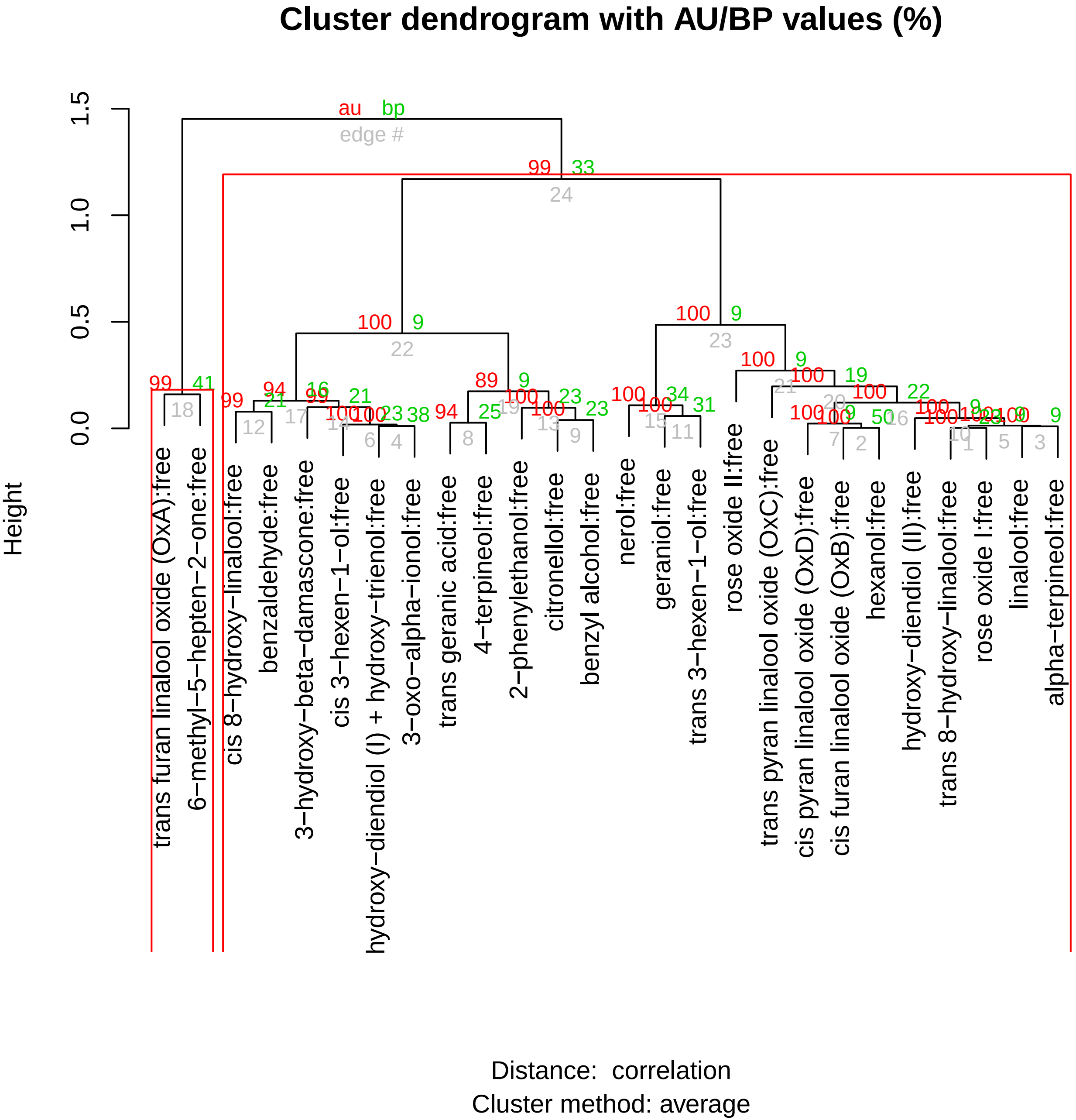

Cluster dendrogram with AU/BP values (%)
au
bp
edge #
Height
0.0
0.5
1.0
1.5
trans furan linalool oxide (OxA):free
6−methyl−5−hepten−2−one:free
cis 8−hydroxy−linalool:free
benzaldehyde:free
3−hydroxy−beta−damascone:free
cis 3−hexen−1−ol:free
hydroxy−diendiol (I) + hydroxy−trienol:free
3−oxo−alpha−ionol:free
trans geranic acid:free
4−terpineol:free
2−phenylethanol:free
citronellol:free
benzyl alcohol:free
nerol:free
geraniol:free
trans 3−hexen−1−ol:free
rose oxide II:free
trans pyran linalool oxide (OxC):free
cis pyran linalool oxide (OxD):free
cis furan linalool oxide (OxB):free
hexanol:free
hydroxy−diendiol (II):free
trans 8−hydroxy−linalool:free
rose oxide I:free
linalool:free
alpha−terpineol:free
Distance: correlation
Cluster method: average

C

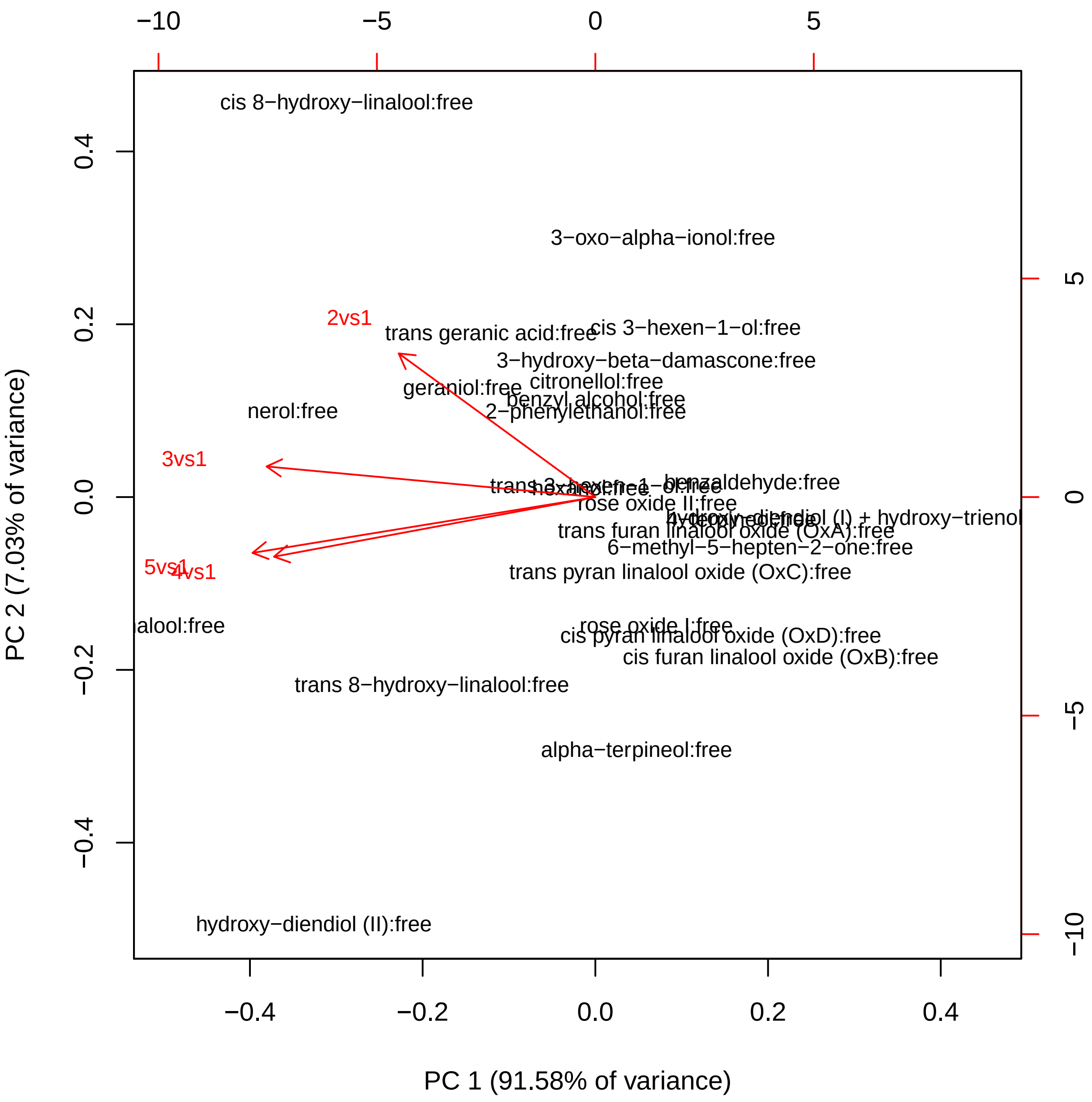

PC 1 (91.58% of variance)
PC 2 (7.03% of variance)
−10
−5
0
5
0.4
0.2
0.0
−0.2
−0.4
2vs1
3vs1
4vs1
5vs1
cis 8−hydroxy−linalool:free
3−oxo−alpha−ionol:free
trans geranic acid:free
cis 3−hexen−1−ol:free
3−hydroxy−beta−damascone:free
citronellol:free
geraniol:free
benzyl alcohol:free
2−phenylethanol:free
nerol:free
trans 3−hexen−1−ol:free
hexanol:free
benzaldehyde:free
rose oxide II:free
hydroxy−diendiol (I) + hydroxy−trienol
trans furan linalool oxide (OxA):free
6−methyl−5−hepten−2−one:free
trans pyran linalool oxide (OxC):free
alool:free
rose oxide I:free
cis pyran linalool oxide (OxD):free
cis furan linalool oxide (OxB):free
trans 8−hydroxy−linalool:free
alpha−terpineol:free
hydroxy−diendiol (II):free

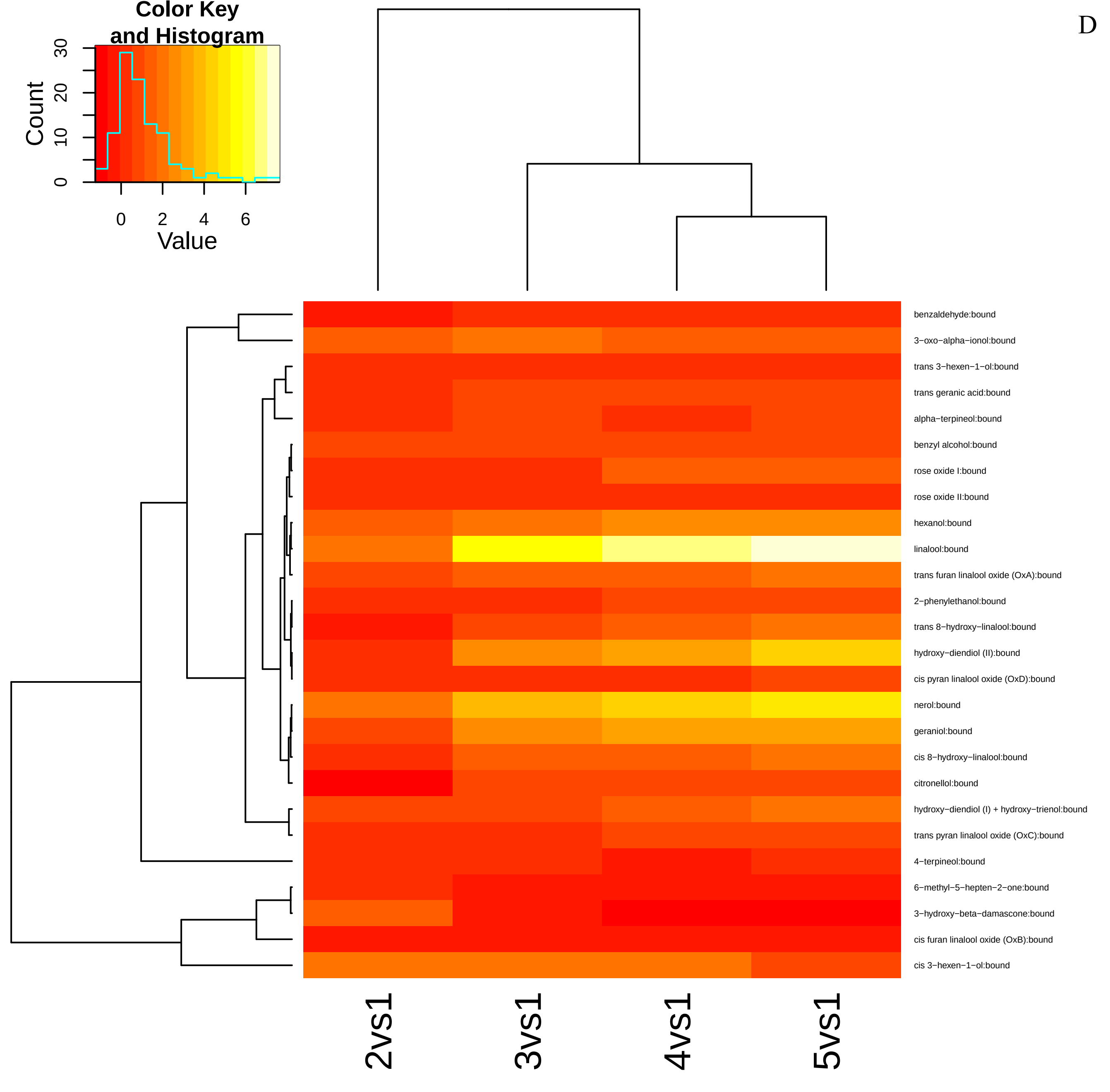

Color Key and Histogram
Count
Value
0
2
4
6
0
10
20
30
D
benzaldehyde:bound
3−oxo−alpha−ionol:bound
trans 3−hexen−1−ol:bound
trans geranic acid:bound
alpha−terpineol:bound
benzyl alcohol:bound
rose oxide I:bound
rose oxide II:bound
hexanol:bound
linalool:bound
trans furan linalool oxide (OxA):bound
2−phenylethanol:bound
trans 8−hydroxy−linalool:bound
hydroxy−diendiol (II):bound
cis pyran linalool oxide (OxD):bound
nerol:bound
geraniol:bound
cis 8−hydroxy−linalool:bound
citronellol:bound
hydroxy−diendiol (I) + hydroxy−trienol:bound
trans pyran linalool oxide (OxC):bound
4−terpineol:bound
6−methyl−5−hepten−2−one:bound
3−hydroxy−beta−damascone:bound
cis furan linalool oxide (OxB):bound
cis 3−hexen−1−ol:bound
2vs1
3vs1
4vs1
5vs1

E

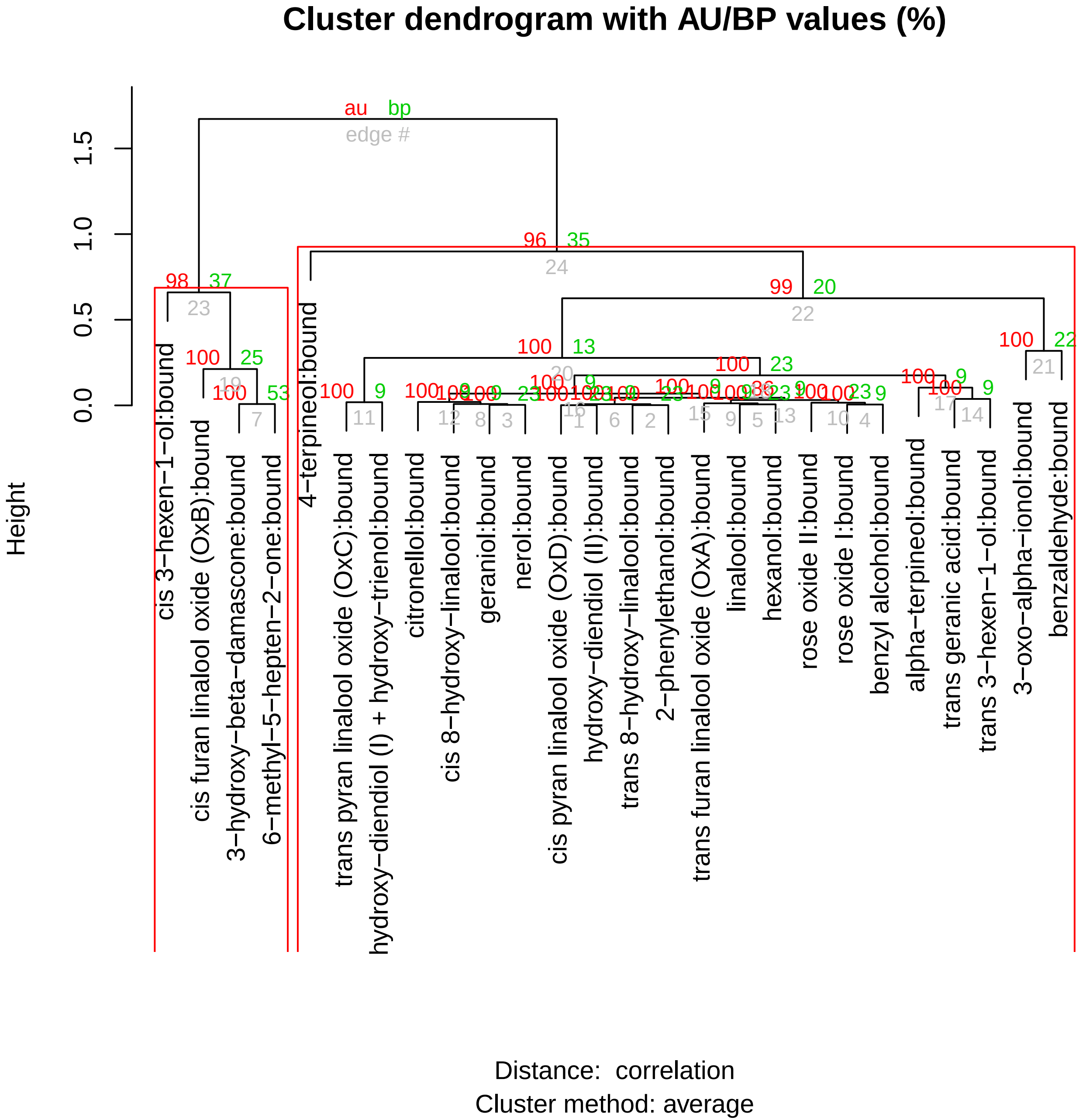

Cluster dendrogram with AU/BP values (%)
au
bp
edge #
Height
0.0
0.5
1.0
1.5
cis 3−hexen−1−ol:bound
cis furan linalool oxide (OxB):bound
3−hydroxy−beta−damascone:bound
6−methyl−5−hepten−2−one:bound
4−terpineol:bound
trans pyran linalool oxide (OxC):bound
hydroxy−diendiol (I) + hydroxy−trienol:bound
citronellol:bound
cis 8−hydroxy−linalool:bound
geraniol:bound
nerol:bound
cis pyran linalool oxide (OxD):bound
hydroxy−diendiol (II):bound
trans 8−hydroxy−linalool:bound
2−phenylethanol:bound
trans furan linalool oxide (OxA):bound
linalool:bound
hexanol:bound
rose oxide II:bound
rose oxide I:bound
benzyl alcohol:bound
alpha−terpineol:bound
trans geranic acid:bound
trans 3−hexen−1−ol:bound
3−oxo−alpha−ionol:bound
benzaldehyde:bound
Distance: correlation
Cluster method: average

F

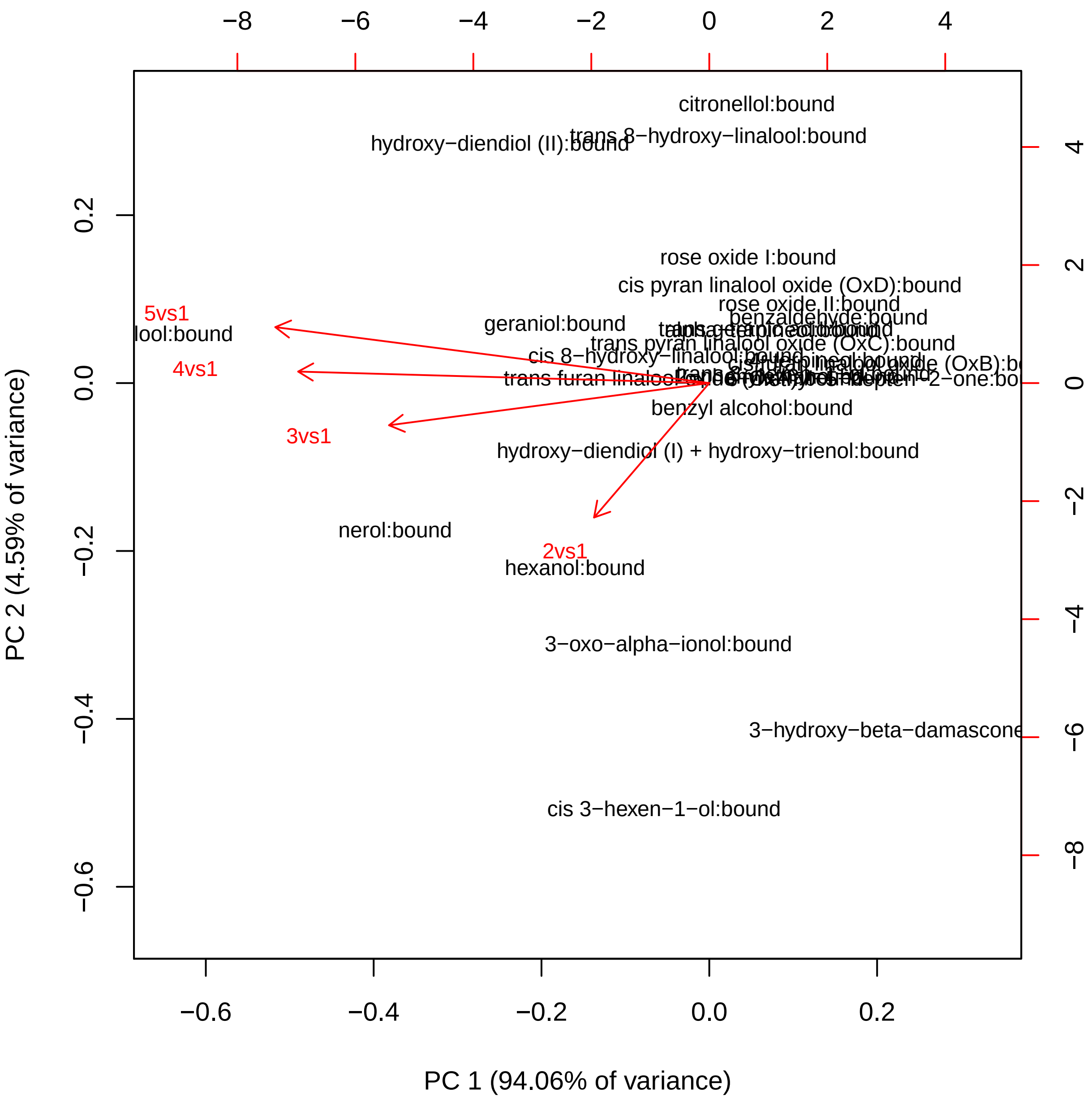

PC 1 (94.06% of variance)
PC 2 (4.59% of variance)
−0.6
−0.4
−0.2
0.0
0.2
−8
−6
−4
−2
0
2
4
citronellol:bound
trans 8−hydroxy−linalool:bound
hydroxy−diendiol (II):bound
rose oxide I:bound
cis pyran linalool oxide (OxD):bound
rose oxide II:bound
benzaldehyde:bound
geraniol:bound
trans pyran linalool oxide (OxC):bound
cis 8−hydroxy−linalool:bound
benzyl alcohol:bound
hydroxy−diendiol (I) + hydroxy−trienol:bound
nerol:bound
hexanol:bound
3−oxo−alpha−ionol:bound
3−hydroxy−beta−damascone
cis 3−hexen−1−ol:bound
5vs1
4vs1
3vs1
2vs1

Supplementary Figure S3: Metabolite grouping based on log2-transformed differentials at stages 2-5 compared to stage 1. Correlation heatmaps are shown in A (free metabolites) and D (bound metabolites) along with Pvclust dendrograms. The outputs of hierarchical clustering with bootstrapping are shown in B (free metabolites) and E (bound metabolites), for which the R/package Pvclust (Suzuki and Shimodaira, 2006) was used with 10,000 resamplings. Two types of p-values are reported: approximately unbiased p-value (AU, calculated with multiscale bootstrap resampling, superior in bias) and bootstrap probability value (BP, calculated by the ordinary bootstrap resampling). Clusters with AU $\geq$ 95 are indicated by red rectangles. Grey numbers represent the number of edges. The outputs of PCA are shown in C (free metabolites) and F (bound metabolites).
