# Supplementary material for: Drawing Links from Transcriptome to Metabolites: The Evolution of Aroma in the Ripening Berry of Moscato Bianco (Vitis vinifera L.)
Source: Front Plant Sci. 2017 May 16;8:780. doi: 10.3389/fpls.2017.00780 (PMC5432621; doi:10.3389/fpls.2017.00780)
Supplement: Supplementary file 15 [file Image4.pdf]

A

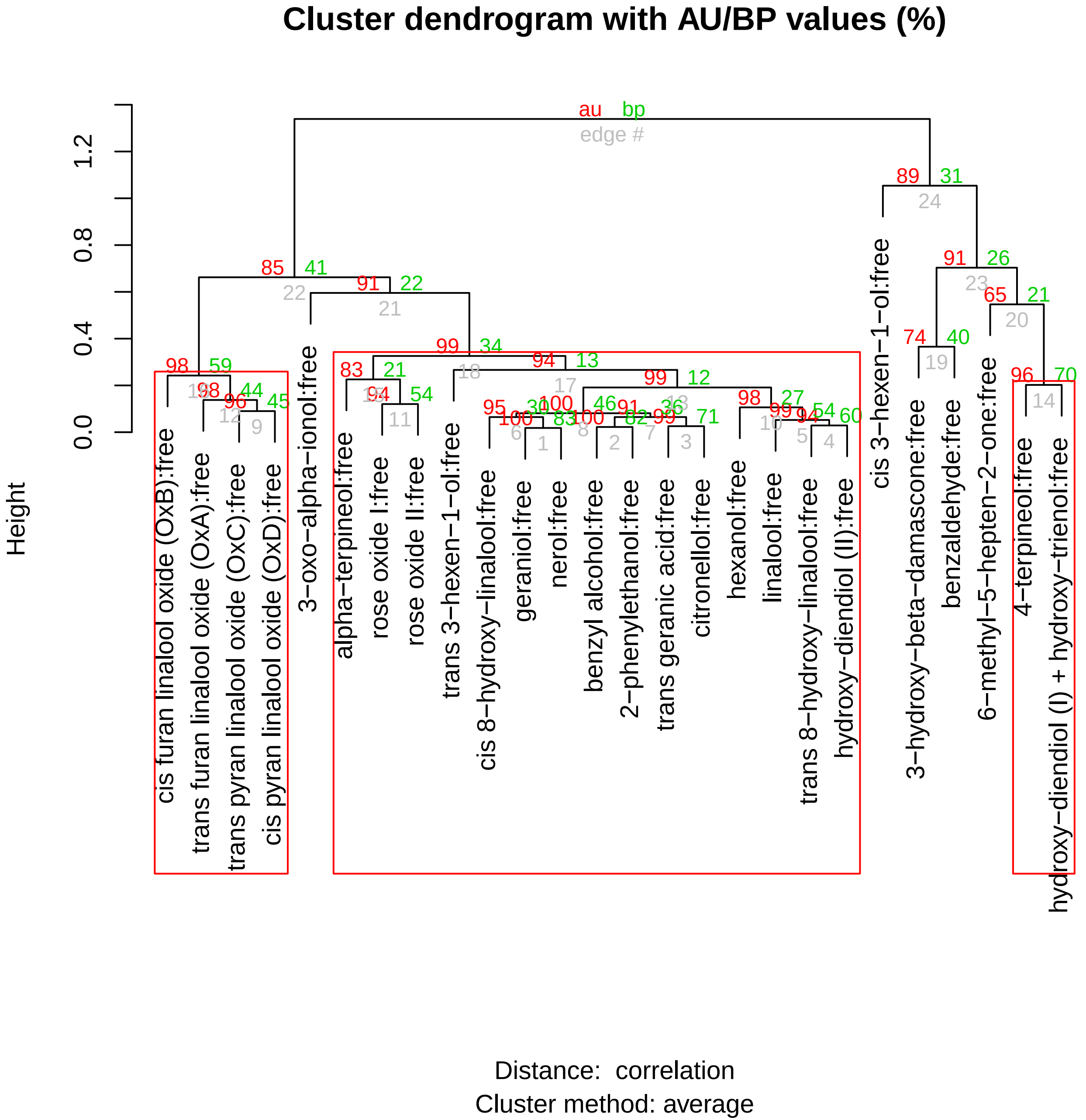

Cluster dendrogram with AU/BP values (%)
au
bp
edge #
Height
0.0
0.4
0.8
1.2
cis furan linalool oxide (OxB):free
trans furan linalool oxide (OxA):free
trans pyran linalool oxide (OxC):free
cis pyran linalool oxide (OxD):free
3−oxo−alpha−ionol:free
alpha−terpineol:free
rose oxide I:free
rose oxide II:free
trans 3−hexen−1−ol:free
cis 8−hydroxy−linalool:free
geraniol:free
nerol:free
benzyl alcohol:free
2−phenylethanol:free
trans geranic acid:free
citronellol:free
hexanol:free
linalool:free
trans 8−hydroxy−linalool:free
hydroxy−diendiol (II):free
cis 3−hexen−1−ol:free
3−hydroxy−beta−damascone:free
benzaldehyde:free
6−methyl−5−hepten−2−one:free
4−terpineol:free
hydroxy−diendiol (I) + hydroxy−trienol:free
Distance: correlation
Cluster method: average

B

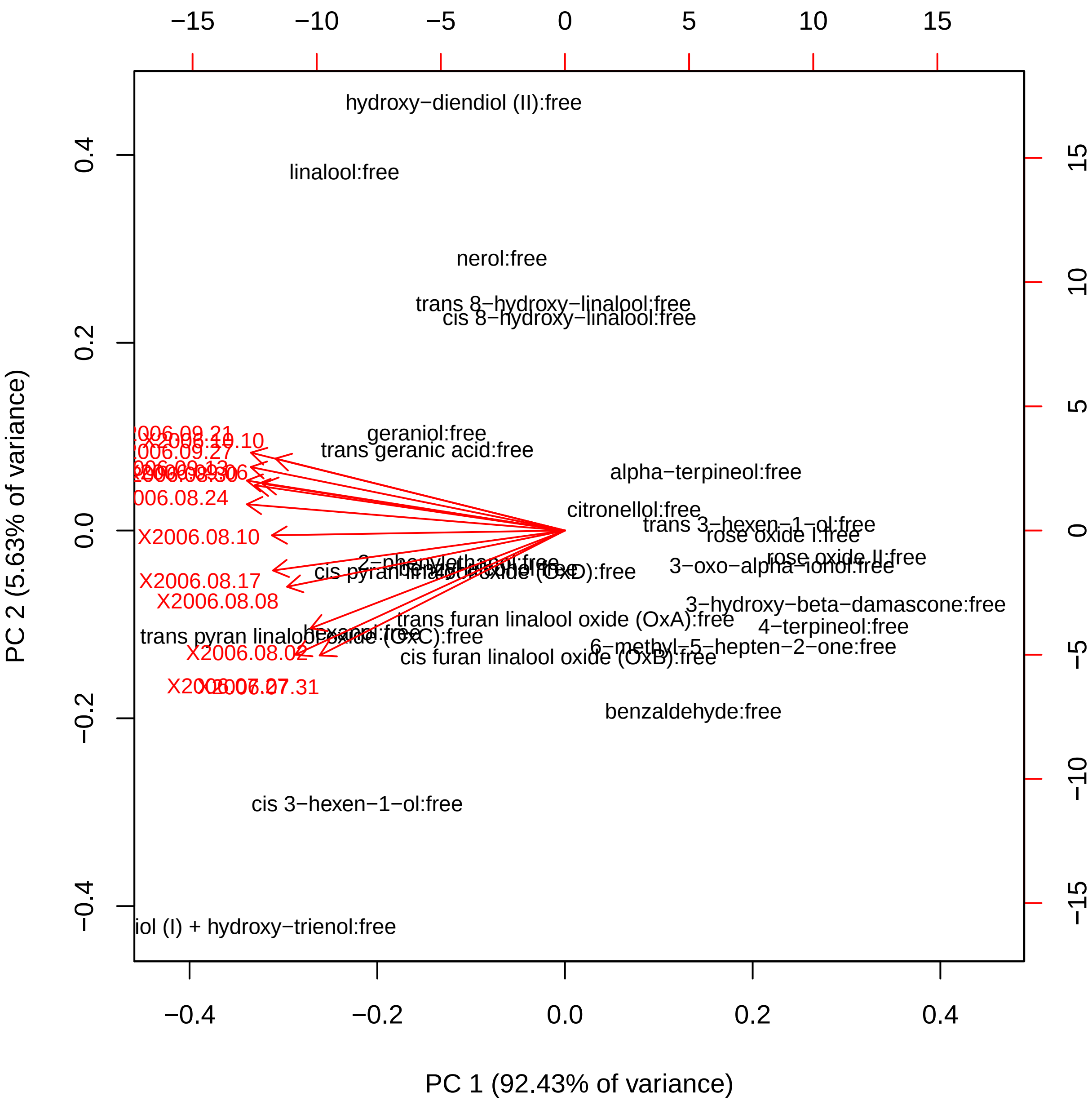
hydroxy−diendiol (II):free
linalool:free
nerol:free
trans 8−hydroxy−linalool:free
cis 8−hydroxy−linalool:free
geraniol:free
trans geranic acid:free
alpha−terpineol:free
citronellol:free
trans 3−hexen−1−ol:free
rose oxide I:free
rose oxide II:free
3−oxo−alpha−ionol:free
2−phenylethanol:free
3−hydroxy−beta−damascone:free
trans furan linalool oxide (OxA):free
4−terpineol:free
trans pyran linalool oxide (OxC):free
6−methyl−5−hepten−2−one:free
cis furan linalool oxide (OxB):free
benzaldehyde:free
cis 3−hexen−1−ol:free
iol (I) + hydroxy−trienol:free
X2006.10.10
006.08.24
X2006.08.10
X2006.08.17
X2006.08.08
X2006.08.02
PC 1 (92.43% of variance)
PC 2 (5.63% of variance)

C

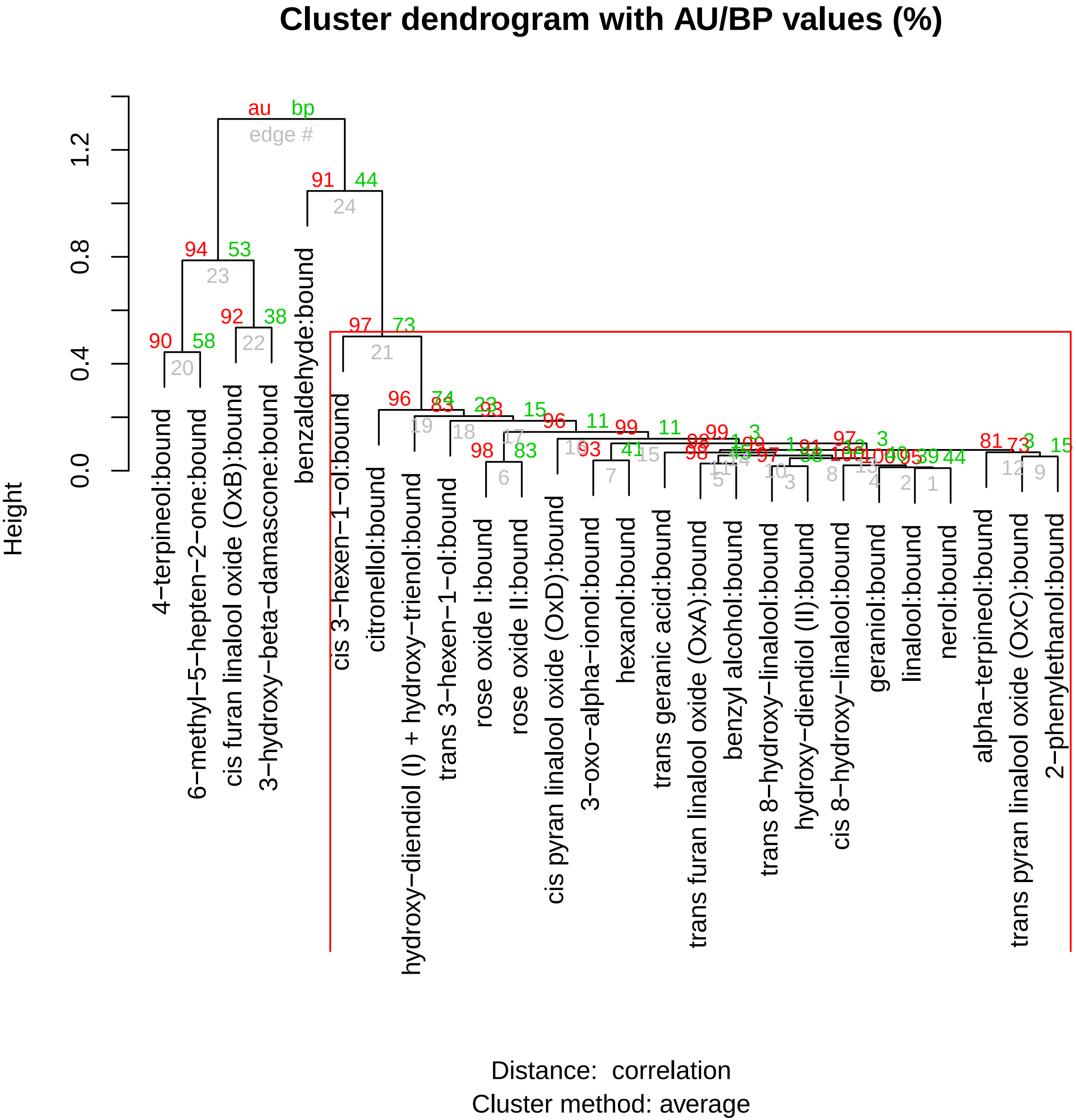
Cluster dendrogram with AU/BP values (%)
au
bp
edge #
Height
0.0
0.4
0.8
1.2
4−terpineol:bound
6−methyl−5−hepten−2−one:bound
cis furan linalool oxide (OxB):bound
3−hydroxy−beta−damascone:bound
benzaldehyde:bound
cis 3−hexen−1−ol:bound
citronellol:bound
hydroxy−diendiol (I) + hydroxy−trienol:bound
trans 3−hexen−1−ol:bound
rose oxide I:bound
rose oxide II:bound
cis pyran linalool oxide (OxD):bound
3−oxo−alpha−ionol:bound
hexanol:bound
trans geranic acid:bound
trans furan linalool oxide (OxA):bound
benzyl alcohol:bound
trans 8−hydroxy−linalool:bound
hydroxy−diendiol (II):bound
cis 8−hydroxy−linalool:bound
geraniol:bound
linalool:bound
nerol:bound
alpha−terpineol:bound
trans pyran linalool oxide (OxC):bound
2−phenylethanol:bound
Distance: correlation
Cluster method: average

D

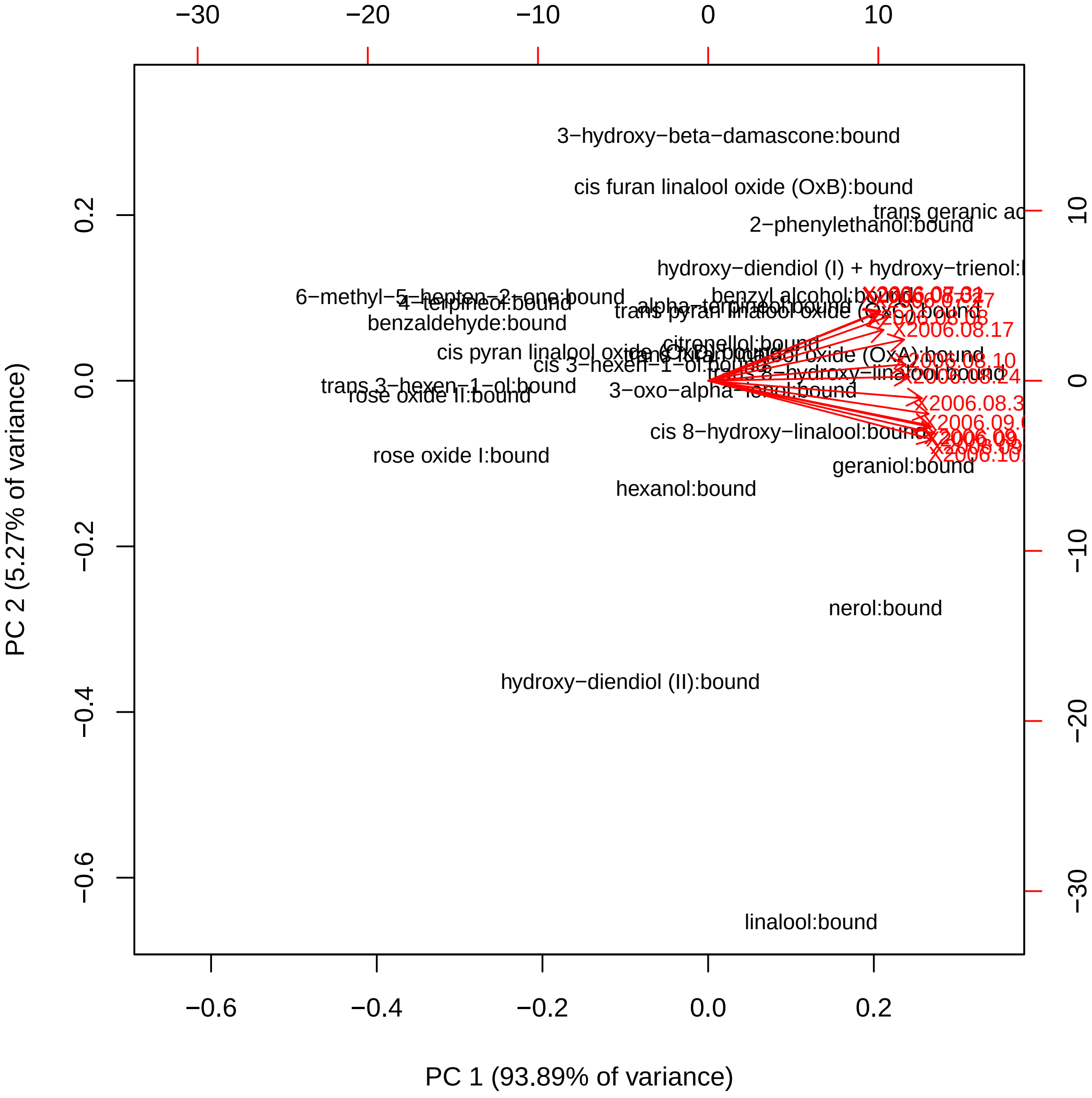

PC 1 (93.89% of variance)
PC 2 (5.27% of variance)
−30
−20
−10
0
10
−0.6
−0.4
−0.2
0.0
0.2
3−hydroxy−beta−damascone:bound
cis furan linalool oxide (OxB):bound
2−phenylethanol:bound
hydroxy−diendiol (I) + hydroxy−trienol:
benzyl alcohol:bound
6−methyl−5−hepten−2−one:bound
4−terpineol:bound
alpha−terpineol:bound
benzaldehyde:bound
citronellol:bound
cis pyran linalool oxide (OxC):bound
cis 3−hexen−1−ol:bound
trans 3−hexen−1−ol:bound
rose oxide II:bound
3−oxo−alpha−ionol:bound
cis 8−hydroxy−linalool:bound
rose oxide I:bound
geraniol:bound
hexanol:bound
nerol:bound
hydroxy−diendiol (II):bound
linalool:bound
X2006.08.17
X2006.08.10
X2006.08.24

Supplementary Figure S4: Grouping of log2-transformed metabolites analyzed at 13 time points. The outputs of hierarchical clustering with bootstrapping are shown in A (free metabolites) and C (bound metabolites), for which the R/package Pvclust (Suzuki and Shimodaira, 2006) was used with 10,000 resamplings. Two types of p-values are reported: approximately unbiased p-value (AU, calculated with multiscale bootstrap resampling, superior in bias) and bootstrap probability value (BP, calculated by the ordinary bootstrap resampling). Clusters with AU ≥ 95 are indicated by red rectangles. Grey numbers represent the number of edges. The outputs of PCA are shown in B (free metabolites) and D (bound metabolites).
