# Supplementary material for: Drawing Links from Transcriptome to Metabolites: The Evolution of Aroma in the Ripening Berry of Moscato Bianco (Vitis vinifera L.)
Source: Front Plant Sci. 2017 May 16;8:780. doi: 10.3389/fpls.2017.00780 (PMC5432621; doi:10.3389/fpls.2017.00780)
Supplement: Supplementary file 16 [file Image5.pdf]

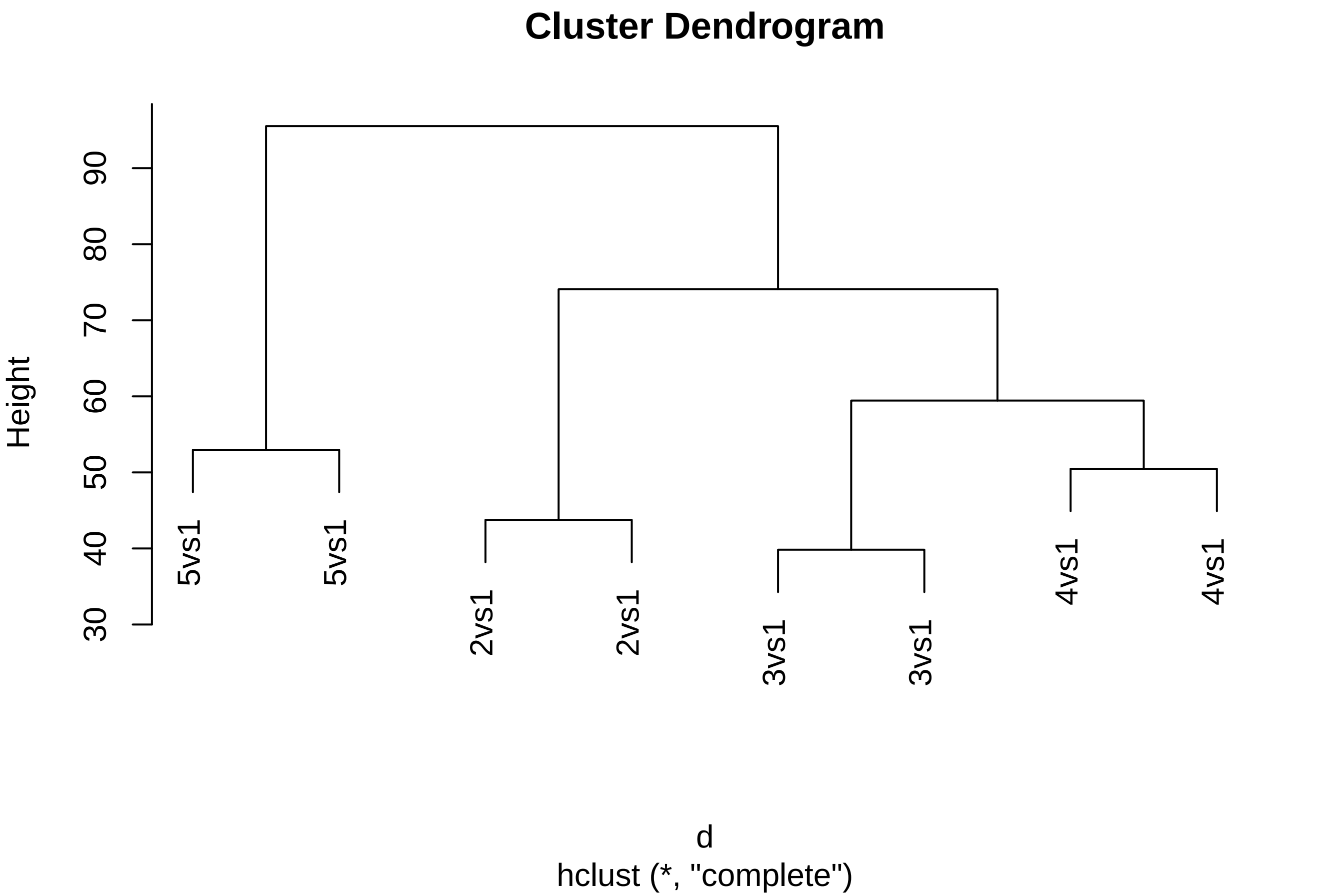
Cluster Dendrogram
Height
30
40
50
60
70
80
90
5vs1
5vs1
2vs1
2vs1
3vs1
3vs1
4vs1
4vs1
d
hclust (*, "complete")

Supplementary Figure S5: Hierarchical clustering of biological replicates. The M-values of the two dye-swaps per sample (normalized log2 fold changes between Cy5 and Cy3) are shown concatenated. 2vs1, 3vs1, 4vs1 and 5vs1 correspond to the pairwise comparison between stages 2-5 and stage 1 from Table 1.
