# Supplementary material for: Drawing Links from Transcriptome to Metabolites: The Evolution of Aroma in the Ripening Berry of Moscato Bianco (Vitis vinifera L.)
Source: Front Plant Sci. 2017 May 16;8:780. doi: 10.3389/fpls.2017.00780 (PMC5432621; doi:10.3389/fpls.2017.00780)
Supplement: Supplementary file 17 [file Image6.pdf]

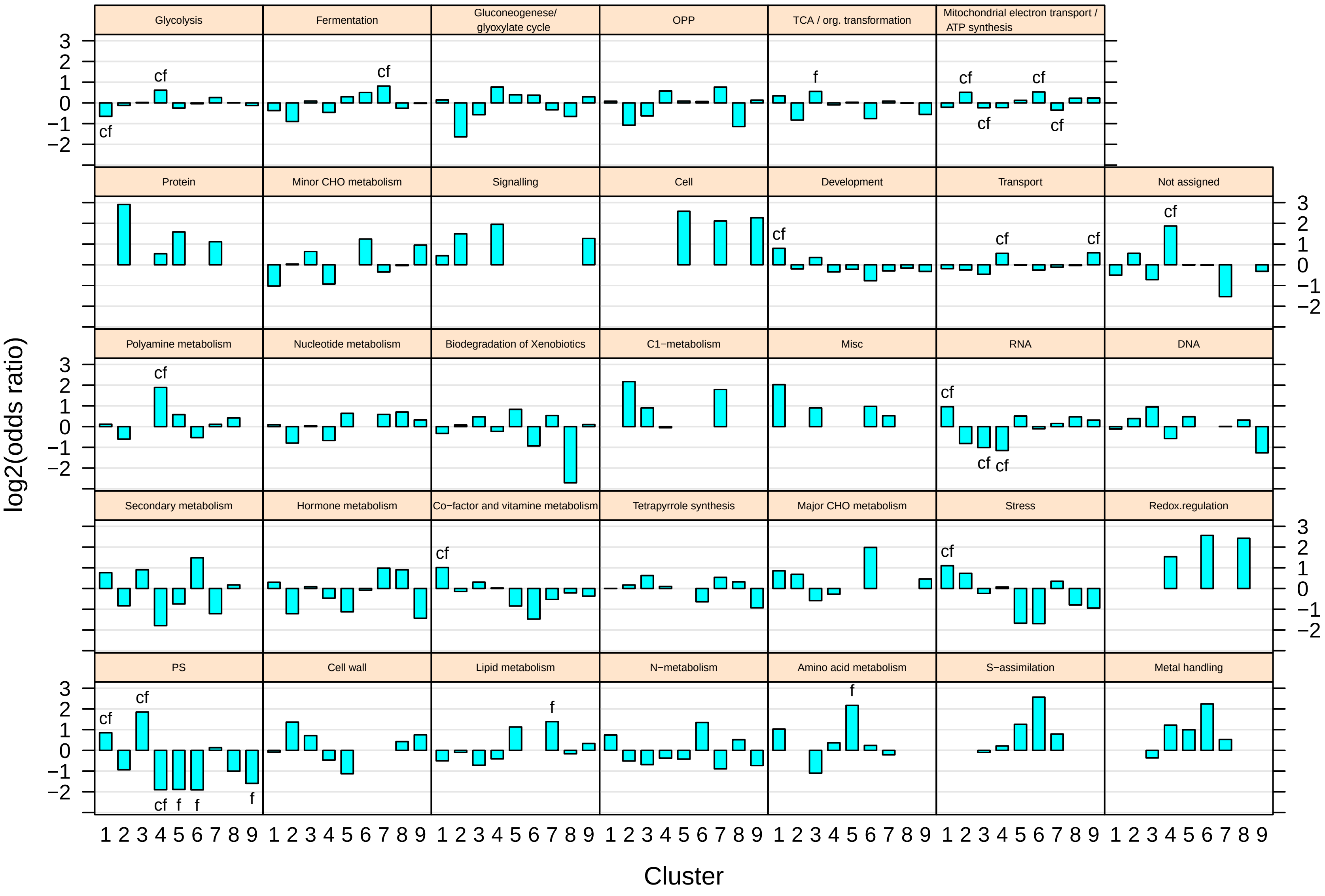

Glycolysis
Fermentation
Gluconeogenese/ glyoxylate cycle
OPP
TCA / org. transformation
Mitochondrial electron transport / ATP synthesis
Protein
Minor CHO metabolism
Signalling
Cell
Development
Transport
Not assigned
Polyamine metabolism
Nucleotide metabolism
Biodegradation of Xenobiotics
C1−metabolism
Misc
RNA
DNA
Secondary metabolism
Hormone metabolism
Co−factor and vitamine metabolism
Tetrapyrrole synthesis
Major CHO metabolism
Stress
Redox.regulation
PS
Cell wall
Lipid metabolism
N−metabolism
Amino acid metabolism
S−assimilation
Metal handling
log2(odds ratio)
Cluster

Supplementary Figure S6: Log2 probe count odds ratios in the nine Mfuzz clusters for MapMan main categories. Significant enrichments or depletions (p-value < 0.05) as determined by Chi square and Fisher tests (without correction for multiple testing) are indicated by the letters c and f respectively.
